# Growth productivity as a determinant of the inoculum effect for bactericidal antibiotics

**DOI:** 10.1126/sciadv.add0924

**Published:** 2022-12-14

**Authors:** Gabriela Diaz-Tang, Estefania Marin Meneses, Kavish Patel, Sophia Mirkin, Laura García-Diéguez, Camryn Pajon, Ivana Barraza, Vijay Patel, Helana Ghali, Angelica P. Tracey, Christopher A. Blanar, Allison J. Lopatkin, Robert P. Smith

**Affiliations:** ^1^ Department of Biological Sciences, Halmos College of Arts and Science, Nova Southeastern University, Fort Lauderdale, FL 33314, USA.; ^2^ Department of Biology, Barnard College, Columbia University, New York, NY10025, USA.; ^3^ Data Science Institute, Columbia University, New York, NY10025, USA.; ^4^ Department of Ecology, Evolution, and Environmental Biology, Columbia University, New York, NY10025, USA.; ^5^ Cell Therapy Institute, Kiran Patel College of Allopathic Medicine, Nova Southeastern University, Fort Lauderdale, FL 33314, USA.

## Abstract

Understanding the mechanisms by which populations of bacteria resist antibiotics has implications in evolution, microbial ecology, and public health. The inoculum effect (IE), where antibiotic efficacy declines as the density of a bacterial population increases, has been observed for multiple bacterial species and antibiotics. Several mechanisms to account for IE have been proposed, but most lack experimental evidence or cannot explain IE for multiple antibiotics. We show that growth productivity, the combined effect of growth and metabolism, can account for IE for multiple bactericidal antibiotics and bacterial species. Guided by flux balance analysis and whole-genome modeling, we show that the carbon source supplied in the growth medium determines growth productivity. If growth productivity is sufficiently high, IE is eliminated. Our results may lead to approaches to reduce IE in the clinic, help standardize the analysis of antibiotics, and further our understanding of how bacteria evolve resistance.

## Teaser

Interactions between growth rate and metabolism can explain inoculum effect for multiple bactericidal antibiotics.

## INTRODUCTION

Antibiotics have fallen victim to their own success as the emergence of antibiotic-resistant bacteria threatens global public health (*1*). Not only has this led to increased mortality (*2*), but it has also led to increased health care costs (*3*). Further confounding the issue is the slow pace at which previously unidentified antibiotic and nonantibioic approaches to treat infections are being developed (*4*). Accordingly, there is a need to understand the mechanisms by which bacteria resist existing antibiotics toward extending their functional shelf life. Most of the research toward understanding antibiotic resistance has focused on the behavior of the individual bacterium (*5*). However, there is growing awareness that bacterial population dynamics can confer resistance (*6*). These include the dissemination of conjugative plasmids conferring antibiotic resistance (*7*), antibiotic-mediated altruistic death (*8*), and inactivation of antibiotics through collective degradation (*9*). A general understanding of how bacterial populations resist antibiotics has bearing on the evolution of resistance itself (*10*), how resistance mechanisms shape microbial ecology in nonclinical settings (*6*), and in the treatment of infectious diseases.

One widely observed mechanism that a population of bacteria can use to tolerate antibiotics is the inoculum effect (IE). Here, the initial density of a bacterial population determines its minimum inhibitory concentration (MIC) (*11*). For a given concentration of antibiotic, a population initiated from a sufficiently high density will grow and survive. Otherwise, for the same concentration of antibiotic, if the initial density of the population is too low, the population will not grow. IE has been observed for nearly all bacteria and antibiotics [e.g., (*12*), SM (Supplementary Materials) Results], can occur with only small differences in the initial population density (*13*), and can arise in the absence of genetically encoded resistance mechanisms (*14*). Resistance to antibiotics owing to IE has been reported in the clinical setting (tables S1 and S2). Increasing antibiotic concentrations to combat IE may not be plausible as high concentrations of antibiotics can have negative health consequences (*15*). Dosing high-density infections with standard antibiotic concentrations has the potential to drive additional resistance mechanisms (*16*). Developing approaches to treat high-density bacterial infections is required.

Most of the mechanisms that have been used to explain IE rely on growth dynamics (*17*) or the ratio of antibiotic to antibiotic target (*18*). Several antibiotic specific mechanisms have also been proposed for IE including induced degradation of the antibiotic target by bacterial proteases (*14*), differential growth rates (*19*), and collective inactivation of antibiotics (*20*). However, these mechanisms cannot explain IE for different antibiotic classes (*14*, *19*), many have yet to be verified experimentally (*17*, *18*), and they generally do not inform of strategies to rationally reduce, or eliminate, IE. To develop strategies to reduce or eliminate IE, it is important to discover a mechanism that can explain IE across antibiotic classes.

While bacterial growth rate was initially thought to be a predictor of antibiotic efficacy, it has been recently shown that bacterial metabolic state better predicts antibiotic efficacy; when growth rate is constant, increasing the concentration of adenosine 5′-triphosphate (ATP) in the cell increases bactericidal antibiotic lethality (*21*). This finding is directly relevant to IE as bacterial density and the concentration of ATP are interrelated. In a closed system, highly dense populations, such as those in stationary phase, have reduced concentrations of ATP due to nutrient depletion (*22*). Populations with faster growth rates, such as those in log phase, have increased ATP concentrations (*23*). Within the context of IE, the average concentration of ATP of a population would be largely determined by its initial density. Bacteria initiated at a high density would experience a short period of growth before entering stationary phase (*24*), which would reduce the concentration of ATP, and thus reduce antibiotic efficacy. For the same carrying capacity, bacteria initiated from a lower density have a longer period of log phase growth before stationary phase. This increases the time over which the concentration of ATP in the cell is high, which would potentiate antibiotic lethality. Although growth and energy utilization can be positively correlated (*25*), they are not strictly coupled and their relationship depends on the growth environment (e.g., nutrients, temperature, etc.). For example, fast growth can coincide with reduced ATP production (*26*), and slow growth can coincide with increased ATP production (*21*). While interactions between cell density and ATP production could explain IE for bactericidal antibiotics, most of the current mechanisms that explain IE do not account for changes in the energy status of the bacteria (summary in SM Results). Accordingly, we ask whether the interaction between growth rate and the concentration of ATP can explain IE for bactericidal antibiotics. Addressing this question may allow for the development of protocols to treat high-density bacterial infections more effectively.

## RESULTS

### Theoretical basis of growth productivity

**Fig. 1.**
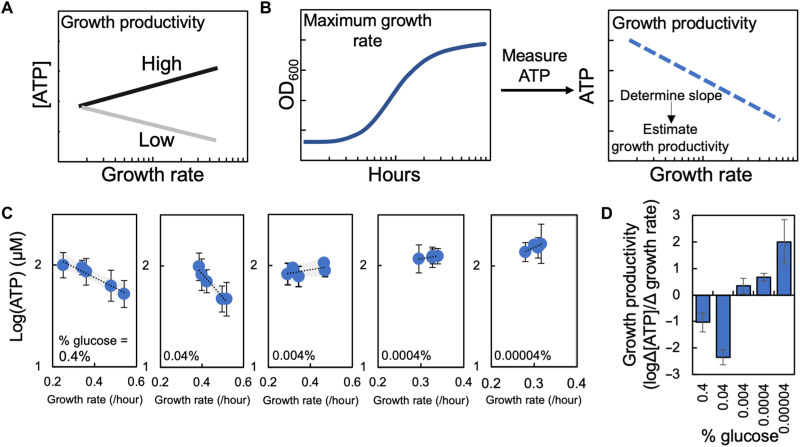
Growth productivity as a metric to quantify the relationship between growth and metabolism. (**A**) If metabolism (i.e., [ATP]) increases at a greater rate than maximum growth rate, growth productivity is high. If metabolism (i.e., [ATP]) decreases with increasing maximum growth rate, growth productivity is low. (**B**) Experimental quantification of growth productivity. We measured maximum growth rate at each percentage of carbon source and casamino acids. To measure metabolism, we measured [ATP] at each percentage of carbon source and casamino acids. Growth productivity for a carbon source was estimated by plotting [ATP] as a function of maximum growth rate. The slope of a linear line through this plot determined growth productivity; a negative slope (shown) indicates low growth productivity, whereas a positive slope indicates high growth productivity. (**C**) [ATP] as a function of maximum growth rate for *E. coli* grown in different percentages of glucose and casamino acids. ATP measurements from three biological replicates (minimum of two technical replicates). Maximum growth rate from three biological replicates. Shading denotes 95% confidence intervals. Error bars denote SD. Growth curves in fig. S1; average residual values from growth curve analysis in table S3. [ATP] was measured below carrying capacity of the medium and is reduced in stationary phase but remains relatively constant during log phase (fig. S2). (**D**) Growth productivity determined from data in (C) plotted as a function of percent glucose in the growth medium. Error bars denote standard error from linear regression used to determine growth productivity. Summary statistics in table S7.

Previous work has demonstrated that both growth rate and the concentration of ATP can be perturbed by growing bacteria in minimal medium supplied with a carbon source and various concentrations of casamino acids, which serve as a nitrogen source (*21*). Both ATP production (*27*) and growth rate (*28*) can be affected by the concentration and identity of a carbon source, together which serves to establish a unique growth environment. As the relationship between growth and the concentration of ATP could potentially explain IE, we developed a simple metric, called growth productivity, to estimate the relationship between growth and the concentration of ATP for each unique growth environment (Fig. 1A). Growth productivity measures the change in the concentration of ATP during log phase growth as a function of the change in maximum growth rate owing to differences in minimal media composition. Numerically, we can estimate growth productivity using the slope of a linear line fit through a plot of various concentrations of ATP as a function of maximum growth rate. By quantifying growth productivity across minimal media that allows different maximum growth rates and concentrations of ATP to be achieved, but with the same carbon source, we can derive a single value for a carbon source at a given percentage that estimates the relationship between growth rate and ATP production. If maximum growth rate is held constant, increasing the concentration of ATP would result in high growth productivity. Alternatively, if the concentration of ATP decreases when maximum growth rate is held constant, growth productivity is low. In scenarios where both growth rate and the concentration of ATP increase, if the increase in the concentration of ATP outpaces the increase in maximum growth rate, growth productivity increases. We focused on ATP as a measure of energy status in the bacteria due to its previously identified and critical role in determining antibiotic lethality (*21*).

To quantify growth productivity, we independently measured maximum growth rate and the concentration of ATP (Fig. 1B). We grew bacteria in minimal medium with different percentages of carbon source and increasing percentages of casamino acids (*21*). Changing the percentage of carbon source and casamino acids alters both growth rate and ATP production (*21*), thus allowing us to examine growth productivity over a wide range of environments. Maximum growth rate was quantified by making high-resolution measurements of cell density in the absence of antibiotics. We then plotted cell density as a function of time and estimated the maximum growth rate by fitting the experimental data using a logistic growth equation (eq. S1, SM Methods). The concentration of ATP during log phase growth and under aerobic conditions was measured in the absence of antibiotics using a bioluminescent assay, a method that is a strong correlate of alternative measures of energy status in bacteria including the ratio of nicotinamide adenine dinucleotide (NAD^+^)/reduced form of NAD^+^ (NADH) and O_2_ consumption rate (SM Results) (*21*). Growth productivity [ATP (micromolar)/growth rate (per hour)] for a given percentage of carbon source was determined by quantifying the slope of a linear line fit through a plot of the concentration of ATP versus maximum growth rate. A negative slope indicates low growth productivity; the concentration of ATP produced decreases with maximum growth rate. Alternatively, a positive slope indicates high growth productivity; the concentration of ATP increases with maximum growth rate. We used a linear regression to approximate and simplify our estimate of growth productivity for a given carbon source (additional justification in SM Results).

### Increasing growth productivity decreases the strength of IE when *Escherichia coli* is grown in medium with glucose

We grew *E. coli* that did not contain extrachromosomal elements conferring antibiotic resistance in M9 medium with different percentages of glucose and casamino acids. We started with glucose as it is inefficiently metabolized at high, but not low, concentrations (*29*) and is a preferred carbon source for *E. coli* (*30*). After measuring maximum growth rate (fig. S1) and the concentration of ATP (Fig. 1C), we quantified growth productivity (Fig. 1D). We observed a biphasic relationship between the percentage of glucose and growth productivity; growth productivity was lowest with 0.04% glucose. Increasing or decreasing glucose away from this concentration increased growth productivity. The greatest growth productivity was observed with 0.00004% glucose.

**Fig. 2.**
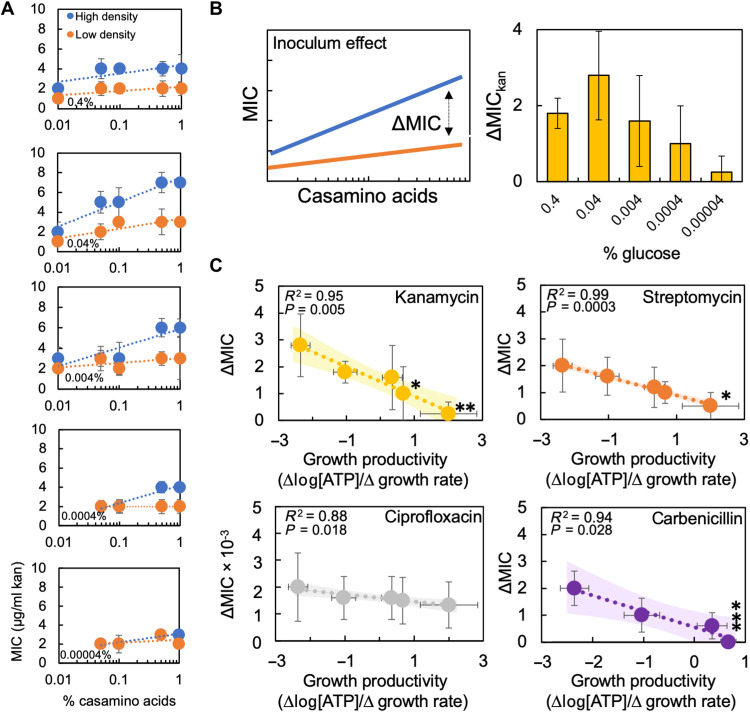
Growth productivity in medium with glucose can determine the strength of IE. (**A**) MIC of kanamycin for high- and low-density populations. SD from ≥3 biological replicates. Raw MIC data in fig. S3. Twenty-four hours of growth is sufficient time to measure IE (fig. S2). We confirmed the presence of IE in kanamycin with a lower initial density (1/500,000×; fig. S2). (**B**) Left: Strength of IE (ΔMIC) = MIC_high_ − MIC_low_ for each percentage of casamino acids for a given concentration of carbon source. Right: ΔMIC_kan_ as a function of percentage of glucose in the medium. Data from (A). SD from ≥3 biological replicates. (**C**) ΔMIC as a function of growth productivity. SD from ≥4 different percentages of casamino acids per percentage of glucose. For each percentage of casamino acids, ΔMIC from ≥3 biological replicates. *ΔMIC was not significantly different from zero (*P = 0.09, **P = 0.20, ***P = 1, one-tailed *t* tests compared to zero). *P* and *R*^2^ on the figure from a linear regression (table S9). Shading denotes 95% confidence interval. *x*-axis error bars denote standard error from regression used to determine growth productivity. Trends were significant using a Deming regression (table S10) and a WLS regression (table S11). Raw data in fig. S4. We found the same trends between ΔMIC and growth productivity when maximum growth rates were determined using a modified Gompertz equation or a k-means clustering analysis (fig. S5), when NAD^+^/NADH was used to measure metabolism, when *E. coli* was grown to midlog phase before initiating MIC experiments, and with a higher density of *E. coli* (1/500×; fig. S6). Maximum growth rate or [ATP] alone could not consistently account for changes in ΔMIC (SM Results and fig. S19).

We then quantified IE under aerobic conditions for the aminoglycoside kanamycin (kan) by measuring the MIC for two populations of *E. coli* strain BW25113 initiated from either high (5000× dilution from an overnight culture that had reached stationary phase; fig. S2) or low (50,000× dilution) density. The initial density of the high initial density population [1.13 × 10^6^ colony-forming units (CFU) per milliliter] was greater than the density recommended for testing MIC in the clinic (5 × 10^5^ CFU/ml) and outside of the acceptable range for MIC testing [2 to 8 × 10^5^ CFU/ml; (*13*, *31*)], thus establishing the clinical relevance of this initial density. As above, we grew *E. coli* in the presence of different percentages of glucose and casamino acids. After 24 hours, we measured cell density [optical density at 600 nm (OD_600_)] and observed that, for a given percentage of glucose, MIC_kan_ increased with the percentage of casamino acids. Moreover, MIC_kan_ for the high initial density population was generally greater than that of the lower initial density population, confirming IE in our system (Fig. 2A).

The strength of IE (ΔMIC) for a given percentage of carbon source was determined using the average difference in MIC between the initial high- and low-density populations; an increase in ΔMIC indicates that IE is enhanced, whereas a decrease indicates a reduction of IE (Fig. 2B, left). Experimentally, we observed a biphasic relationship between ΔMIC_kan_ and the percentage of glucose in the growth medium (Fig. 2B, right). ΔMIC_kan_ peaked at a glucose percentage of 0.04%; increasing or decreasing the percentage of glucose from 0.04% led to a reduction in ΔMIC_kan_. When ΔMIC_kan_ was plotted as a function of growth productivity, ΔMIC_kan_ decreased with increasing growth productivity [simple linear regression, *R*^2^ = 0.95, *P* = 0.005, Fig. 2C; Deming regression, *P* = 0.005 and weighted least squares (WLS) regression, *P* = 0.01; the two former methods of regression account for error in measuring growth productivity]. At the lowest growth productivity measured, ΔMIC_kan_ was greatest and was greater than zero (*P* = 0.004, one-tailed *t* test). If growth productivity was sufficiently high, ΔMIC_kan_ was not different from zero (*P* ≥ 0.09, one-tailed *t* test; *P* values henceforth found in figure legends).

To determine the generality of this finding, we then measured ΔMIC for an additional aminoglycoside, streptomycin. Consistent with kanamycin, as growth productivity increased (Fig. 2C), ΔMIC decreased, and ΔMIC was not significantly different from zero if growth productivity was sufficiently high. Next, we measured ΔMIC for two additional antibiotics that were representative of the major bactericidal classes: carbenicillin (β-lactams) and ciprofloxacin (fluoroquinolones). Combined, these antibiotics cover the three main bactericidal modes of action (*32*). Similar to kanamycin and streptomycin, both carbenicillin and ciprofloxacin showed high ΔMIC under low growth productivity conditions, and ΔMIC decreased with increasing growth productivity (Fig. 2C). ΔMIC of carbenicillin was not significantly different from zero under the highest growth productivity condition. The relationship between ΔMIC and growth productivity remains consistent when NAD^+^/NADH is used to measure metabolism (fig. S6). Initiating MIC experiments using bacteria in midlog (as opposed to stationary) does not change the relationship between ΔMIC_kan_ and growth productivity (fig. S6). The number of antibiotic targets as a result of changes in growth and metabolism is not correlated with ΔMIC_kan_ (fig. S7). The presence of efflux pumps and the endogenous *ampC* β-lactamase found in *E. coli* strain BW25113 did not affect the qualitative trends in IE as determined using knockout strains (fig. S7). For a discussion of alternative hypotheses that could explain IE, including the role of growth and metabolism independently, please see SM Results.

### Model-guided perturbation of growth and metabolism alters growth productivity and ΔMIC

To gain a general intuition into the interactions between growth, metabolism, and antibiotic lethality, we created an abstracted mathematical model (see Materials and Methods; Eq. 1). Bacteria (*N*) grow according to logistic growth scaled as a function of basal growth rate (μ; per hour) and metabolism (ε; millimoles per gram per hour). ε is an approximation of a maintenance coefficient (*33*), which is the amount of energy that is not directly used to create biomass. Consistent with our experimental definition of growth productivity, the relationship between μ and ε defines growth productivity; if μ is held constant, increasing ε, which approximates an increase in [ATP], increases growth productivity. A modified Michaelis-Menten equation describes per-capita death of bacteria due to antibiotics, which is dependent on the initial density of the population, the concentration of antibiotic (*A*), and an antibiotic killing rate (*b*). If growth due to the relative values of μ and ε outpaces per-capita antibiotic death, the population lives; otherwise, if antibiotic death is greater than growth, the population perishes. Reducing initial *N* reduces the logistic growth term in our model, which increases the effect of per-capita death due to antibiotics. This decreases the initial density at which bacteria die.

**Fig. 3.**
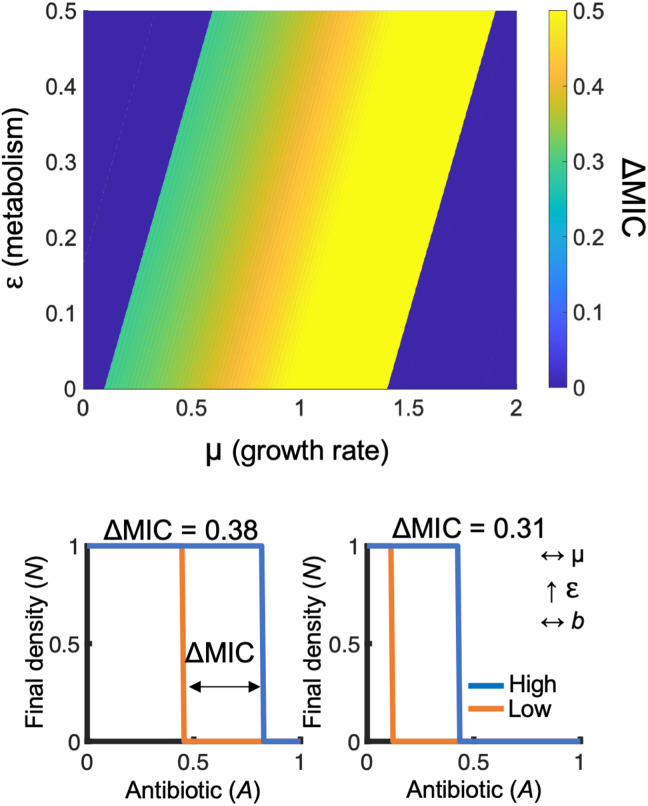
A mathematical model (Eq. 1) captures the qualitative trends of metabolism, growth rate, and ΔMIC. **Top**: Heatmap showing the interactions between growth rate (μ), metabolism (ε), and ΔMIC. Initial *N*_high_ = 5 × 10^−2^. Initial *N*_low_ = 10^−4^. **Bottom**: Left: For given values of μ and ε, simulation initiated with low initial *N* go extinct at a lower value of *A* as compared to high initial *N*. The difference between the lines determines ΔMIC. Right: Increasing ε at constant μ (increasing growth productivity) reduces ΔMIC. Parameters in table S4. Sensitivity analysis in fig. S8. Bottom left: μ = 0.6, ε = 0.055; bottom right: μ = 0.6, ε = 0.385. For all simulations: *t* = 24 hours. ΔMIC shown above each simulation.

Our simulations predict IE. For given values of μ and ε, if the initial density of the population (*N*) is sufficiently high (*N*_high_), the populations will grow. Otherwise, for the same value of *A*, if initial *N* is low (*N*_low_), the population will die. The difference in the smallest values of *A* where *N*_high_ and *N*_low_ do not grow captures ΔMIC. Over a wide range of values, our simulations show that for a given μ, increasing ε increases growth productivity and reduces ΔMIC (Fig. 3). Our simulations allowed us to gain a general intuition into the relationship between the combined effect of growth (μ) and metabolism (ε) on IE.

**Fig. 4.**
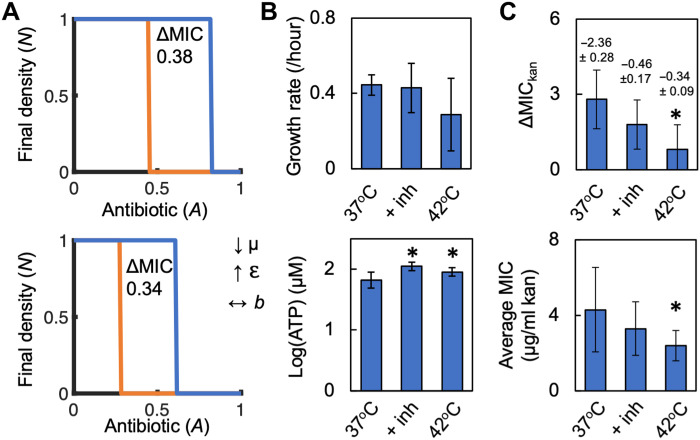
Model-guided manipulation of growth rate and metabolism alters growth productivity and ΔMIC. (**A**) Our model predicts that increasing ε and reducing μ increase growth productivity, reduce ΔMIC, and decrease the value of *A* at which high- and low-density populations go extinct. Top: μ = 0.6, ε = 0.055; bottom: μ = 0.48, ε = 0.11. Parameters in table S4. (**B**) Average maximum growth rate (top) and [ATP] (bottom) of *E. coli* grown at 37°C, at 42°C, or at 37°C with 10 ng/ml of serine protease inhibitor (+ inh) in medium with 0.04% glucose. Maximum growth rate and [ATP] averaged from five different casamino acid concentrations, each consisting of three biological replicates. *Increase in [ATP] (*P* ≤ 0.049, one-tailed *t* test). Error bars denote SD. Data for 37°C from Fig. 1C. For (B) and (C), raw data in fig. S9. Average residuals for growth curve fitting in table S3. (**C**) Top: ΔMIC_kan_ of *E. coli* grown at 37°C, 42°C, or at 37°C + inh. Growth productivity indicated above each bar with standard error shown (from linear regression used to determine growth productivity). *Decrease in ΔMIC (*P* = 0.033, one-tailed *t* test). Bottom: Average MIC from initial high- and low-density populations of *E. coli*. *Significant decrease in average MIC (*P* = 0.017, one-tailed *t* test). MIC averaged from five percentages of casamino acids, each with ≥3 biological replicates. Error bars denote SD. Similar trends were observed when using a modified Gompertz equation to determine maximum growth rate (fig. S9).

Our model predicts that increasing ε and decreasing μ will increase growth productivity. This perturbation is predicted to decrease ΔMIC (Fig. 4A). In addition, owing to increased metabolism (ε), the model predicts an increase in antibiotic efficacy, which can be quantified as a reduction in the average MIC of the high- and low-density populations when considered separately. Averaging the MIC values of these populations can be used to measure changes in antibiotic efficacy due to metabolism and outside of density-dependent effects. To test these predictions, we grew *E. coli* in kanamycin at 42°C or at 37°C with a serine protease inhibitor. As reported previously, these perturbations increased [ATP] (*34*, *35*) and reduced maximum growth rate (Fig. 4B) (*36*), thus increasing growth productivity (Fig. 4C). As predicted by our model, this decreased ΔMIC_kan_ and the average MIC_kan_ of the high- and low-density populations (Fig. 4C). The relative decrease in ΔMIC_kan_ and average MIC_kan_ followed the relative increase in growth productivity.

**Fig. 5.**
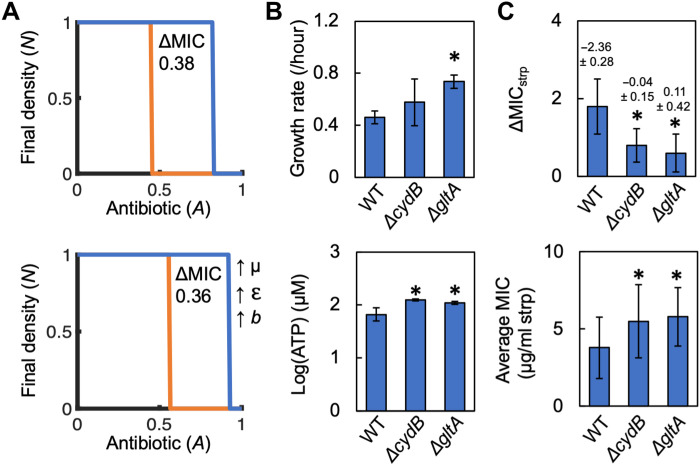
Model-guided manipulation of growth rate and metabolism using *E. coli* strains that lack genes in the tricarboxylic acid cycle and electron transport affects growth productivity and ΔMIC. (**A**) Our model predicts that increasing *b*, ε, and μ increases growth productivity, decreases ΔMIC, and increases average MIC. Top: μ = 0.6, ε = 0.055, and *b* = 0.1; bottom: μ = 0.84, ε = 0.0935, and *b* = 0.117. Parameters in table S4. (**B**) Average maximum growth rate (top) and [ATP] (bottom) of *E. coli* knockout strains Δ*gltA* and Δ*cydB*. *Significant increase in growth rate or [ATP] (*P* < 0.001 for Δ*gltA* growth rate, *P* = 0.006 for Δ*cydB* ATP, *P* = 0.013 for Δ*gltA* ATP, one-tailed *t* tests). Maximum growth rate averaged from four different casamino acid concentrations, each consisting of three biological replicates. [ATP] averaged from three biological replicates (three technical replicates) for each of four different casamino acid concentrations. Error bars denote SD. Data for 37°C and wild type (WT) from Fig. 1C. Raw data in fig. S9. Average residuals for curve fitting in table S3. (**C**) Top: ΔMIC of Δ*gltA* and Δ*cydB* grown in streptomycin (strp). Growth productivity indicated above each bar with standard error shown (from linear regression used to determine growth productivity). *Significant decrease in ΔMIC (*P* = 0.016 for Δ*cydB*, *P* = 0.028 for Δ*gltA*, one-tailed *t* tests). Bottom: Average MIC from both high and low initial density populations of Δ*gltA* and Δ*cydB*. *Significant increase in average MIC of both populations (*P* = 0.001 for Δ*cydB*, *P* = 0.008 for Δ*gltA*, one-tailed *t* tests). Similar trends were observed when using a modified Gompertz equation to determine maximum growth rate (fig. S9). Error bars denote SD from ≥3 biological replicates.

Our model predicts that interactions between ε, μ, and *b* determine ΔMIC. When holding ε and μ constant, increasing *b* would decrease average MIC by increasing antibiotic lethality. However, changes in ε and μ can offset increases to *b*. If both ε and μ are increased, this can offset the increase in *b* resulting in an increase to average MIC. This prediction is consistent with previous experimental data showing that increasing [ATP] and growth rate reduces antibiotic lethality (*21*). Our model predicts that if the increase in ε is greater than μ, growth productivity increases, which reduces ΔMIC even when the average MIC of the high- and low-density populations increases (Fig. 5A). To test these predictions, we used *E. coli* that lacked *gltA* and *cydB*, genes that are involved in the tricarboxylic acid cycle and electron transport, respectively. Removal of genes involved in central carbon metabolism has been reported to increase cellular respiration and ATP production, which potentiates antibiotic lethality (*37*). Both strains showed increased [ATP] and maximum growth rate, the former of which was greater (Fig. 5B). As predicted by the model, this increased growth productivity and average MIC_kan_ but reduced ΔMIC_kan_ (Fig. 5C). These simulations and experiments provide support for the role that growth productivity plays in IE.

### Growth productivity determines ΔMIC for multiple species of bacteria

**Fig. 6.**
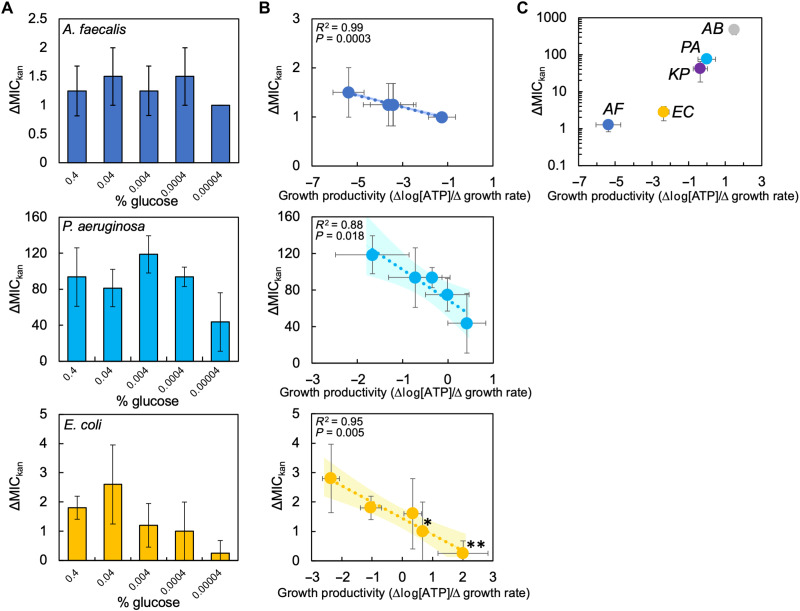
Growth productivity determines the strength of IE for multiple bacterial species. (**A**) ΔMIC_kan_ as a function of % glucose y for *A. faecalis*, *P. aeruginosa*, and *E. coli* (from Fig. 2B). SD from ≥3 biological replicates. For (A) and (B), SD from ≥4 different percentages of casamino acids per percentage of glucose. Raw data in fig. S10. Average residuals for growth curve fitting in table S3. (**B**) ΔMIC_kan_ as a function of growth productivity. *ΔMIC_kan_ not greater than zero (*P = 0.09, **P = 0.20, one-tailed *t* tests). *P* and *R*^2^ values on the figure from a linear regression. Shading denotes 95% confidence interval. Trends were significant using a Deming regression (table S10) and a WLS regression (table S11). Error bars: *x* axis = standard error from linear regression used to determine growth productivity, *y* axis = SD. Raw growth productivity data in fig. S10A. (**C**) ΔMIC_kan_ as function of growth productivity for multiple bacterial species. Glucose = 0.04%. AB = *A. baumannii*, EC = *E. coli*, KP = *K. pneumoniae*, AF = *A. faecalis*, and PA = *P. aeruginosa*. We did not find a significant linear correlation between ΔMIC_kan_ and [ATP] or maximum growth rate for *P. aeruginosa* and *A. faecalis*. Error bars: *x* axis = standard error from linear regression used to determine growth productivity, *y* axis = SD. We did not find a significant linear correlation between ΔMIC_kan_ and [ATP] or maximum growth rate when all species were considered (glucose = 0.04%; fig. S10 and SM Results).

Bacterial species differ in their growth requirements. For a given carbon source, each species could have a unique growth productivity and thus a different ΔMIC. To test this, we determined growth productivity and ΔMIC_kan_ with glucose and in aerobic conditions for *Alcaligenes faecalis* and *Pseudomonas aeruginosa*. The percentage of glucose in the medium had different effects on ΔMIC_kan_ for each species (Fig. 6A). As growth productivity increased, ΔMIC_kan_ decreased (Fig. 6B). For the same percentage of glucose (0.04%), each species had a unique growth productivity. To explore this, we quantified growth productivity (0.04% glucose) and ΔMIC_kan_ for *Acinetobacter baumannii* and *Klebsiella pneumoniae*. Each species had its own growth productivity (Fig. 6C). When we grew *P. aeruginosa* in medium that contained acetate, which is a preferred carbon source relative to glucose (*38*), growth productivity increased, which resulted in a decrease in ΔMIC_kan_ as compared to when glucose was used in the growth medium (fig. S10). Thus, although increasing growth productivity can reduce IE for bacteria other than *E. coli*, species-specific carbon source preferences dictate growth productivity. We did not find a significant relationship between ΔMIC_kan_ and maximum growth rate or [ATP] when all bacterial species were considered (SM Results and fig. S10).

### When growth productivity, as determined by carbon source, is sufficiently high, IE can be abolished

**Fig. 7.**
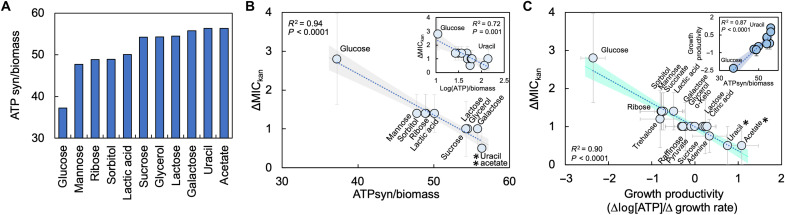
FBA predicts the effects of different carbon sources on growth productivity. (**A**) In silico prediction of growth productivity [ATP synthase activity (ATPsyn)/biomass] using FBA. Parameters in table S5. (**B**) ΔMIC_kan_ as a function of FBA-predicted growth productivity. *P* and *R*^2^ values from linear regression. Shading denotes 95% confidence interval. SD from ≥3 biological replicates. Inset: ΔMIC_kan_ versus growth productivity calculated using experimental [ATP] and FBA-predicted biomass. SD from ≥3 biological replicates and ≥4 percentages of casamino acids. *ΔMIC_kan_ is not greater than zero (*P = 0.091, one-tailed *t* test). Sensitivity analysis for FBA—fig. S11. Raw data—fig. S12. (**C**) ΔMIC_kan_ as a function of experimentally determined growth productivities. Carbon source = 0.04%. *ΔMIC not greater than zero (*P = 0.091). Raw MIC data—fig. S12. Growth curves—fig. S13. Raw growth productivity data—fig. S14. *P* and *R*^2^ from linear regression (table S9). Shading denotes 95% confidence interval. Trends were significant using Deming (table S10) or WLS regression (table S11). Inset: Correlation between FBA predicted and experimentally determined values of growth productivity. SD from ≥3 biological replicates (*y* axis). *x*-axis error bars denote standard error from linear regression used to determine growth productivity. ΔMIC_kan_ decreases with growth productivity, and there is a positive correlation between FBA-predicted and experimental values of growth productivity when alternative methods to determine growth rate are used (fig. S15). Growth productivity values are correlated when calculated using different approaches to determine growth rate (fig. S15).

Because each bacterial species studied had a different growth productivity for the same percentage of glucose, we wondered how growth productivity and ΔMIC would be affected by carbon sources other than glucose. Carbon sources can alter both ATP production (*39*) and growth rate (*40*). Given the breadth of carbon sources that we could test, we used flux balance analysis (FBA) coupled with a whole-genome model of *E. coli* to predict which carbon sources would reduce growth productivity. Using published flux parameters (table S5), we simulated biomass accumulation and ATP synthase activity for 10 carbon sources in addition to glucose. Growth productivity was determined as the ratio of ATP synthase activity to biomass accumulation. Our FBA predicts a range of growth productivities; glucose had the smallest growth productivity, whereas uracil and acetate had the greatest (Fig. 7A). Thus, we predict that glucose will have the greatest ΔMIC, whereas uracil and acetate will have the smallest.

To test these predictions, we grew *E. coli* in the 11 carbon sources studied in our FBA and determined ΔMIC_kan_ under aerobic conditions. To protect against concentration-dependent changes in ΔMIC and growth productivity, the percentage of carbon source was constant (0.04%). Carbon sources with the smallest (glucose) and greatest (acetate/uracil) FBA-predicted growth productivities had the largest and smallest values of ΔMIC_kan_, respectively (Fig. 7B). A range of ΔMIC values were found between these two extremes for the additional carbon sources. The relationship between ΔMIC_kan_ and growth productivity remained consistent and significant when growth productivity was calculated using experimentally derived ATP concentrations and final biomass as computed using FBA (Fig. 7B, inset), indicating a general qualitative agreement between experimentally derived concentrations of ATP and those predicted by FBA.

To validate the FBA-predicted trends in growth productivity, we quantified growth productivity for all 11 carbon sources and an additional 7 carbon sources that lacked published flux parameters. We observed a significant negative linear correlation between ΔMIC_kan_ and growth productivity (Fig. 7C). ΔMIC_kan_ for uracil and acetate, which represented the two greatest growth productivities measured, was no different from zero. We found a strong positive correlation between FBA-predicted growth productivity and experimentally measured growth productivity for each carbon source (Fig. 7C, inset). Last, we tested the significance of the slope from Fig. 7C by generating 2500 bootstrapped slopes and determined whether these bootstrapped slopes were significantly different from zero using a *t* test. The relationship between ΔMIC_kan_ and growth productivity was significantly different from zero (*P* < 0.001). We note that the concentration of ATP measured in our experiments matches previous published trends and that trends in growth productivity remain consistent when [ATP] is measured at different times during log phase growth (fig. S14). A significant linear relationship between growth productivity and ΔMIC_kan_ continues to be observed when growth productivity for insignificant relationships between [ATP] and maximum growth rate (as determined using a linear regression) are removed from the analysis (fig. S15). A reduction in ΔMIC_kan_ at higher growth productivities cannot be due to a slow growth rate, which would impede the ability of the low-density population to reach carrying capacity (fig. S16). Last, the relationship between growth productivity and ΔMIC_kan_ remains significant when equimolar concentrations of amino acids, as opposed to casamino acids, are used (fig. S17).

**Fig. 8.**
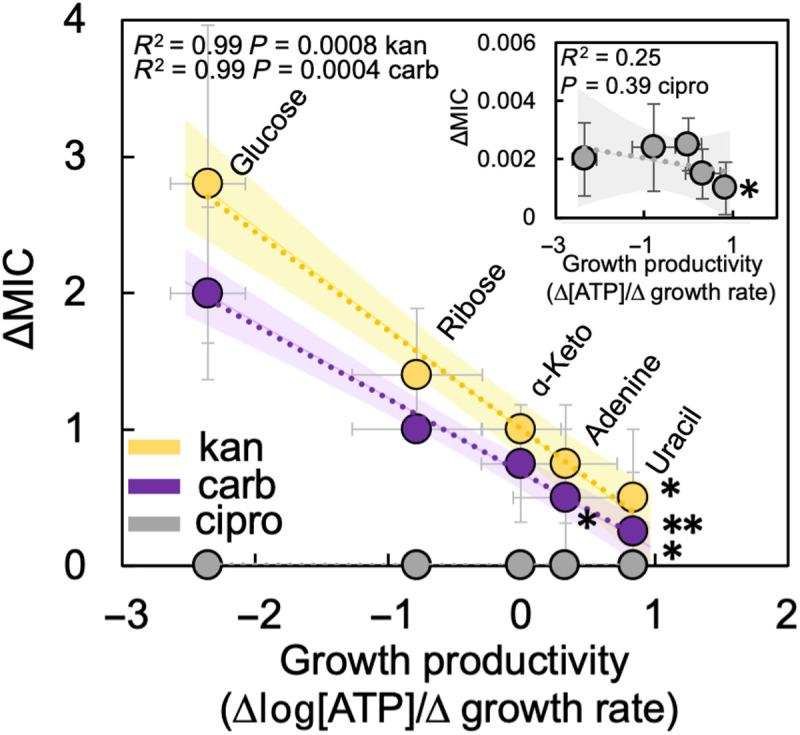
Increasing growth productivity decreases ΔMIC for multiple antibiotics. ΔMIC of kanamycin (kan), carbenicillin (carb), and ciprofloxacin (cipro) for *E. coli* grown in representative carbon sources (0.04%) covering the range of growth productivities. *ΔMIC is not greater than zero (*P = 0.091, **P = 0.21, one-tailed *t* test). *R*^2^ and *P* value from a simple linear regression shown in the figure (summary statistics in table S9). Trends were also significant using a Deming regression (table S10) or WLS regression (table S11). Raw MIC data in fig. S18. Inset: ciprofloxacin data replotted with a smaller *y* axis. ΔMIC decreases as a function of growth productivity when a modified Gompertz equation or k-means clustering analysis is used to determine maximum growth rate (fig. S18). Data from a minimum of three biological replicates. Error bars: *x* axis = standard error from linear regression used to determine growth productivity, *y* axis = SD.

To determine whether carbon source–determined growth productivity could reduce ΔMIC for additional antibiotics, we grew *E. coli* in five carbon sources that spanned the range of growth productivities. As growth productivity increased, ΔMIC decreased for carbenicillin and ciprofloxacin (Fig. 8, see SM Results for explanation as to why the relationship between ciprofloxacin and growth productivity is not significant). Consistent with Fig. 2C, when growth productivity was sufficiently high (uracil), ΔMIC of carbenicillin and ciprofloxacin was no different from zero. The concentration of ATP, maximum growth rate, and pH were not consistently or significantly associated with ΔMIC_kan_ across all growth conditions, bacterial species, and antibiotics, providing further support of growth productivity as a critical determinant of IE (SM Results, figs. S10 and S19).

## DISCUSSION

Our analysis suggests that growth productivity can account for IE across multiple classes of bactericidal antibiotics and in multiple species of bacteria. As the growth environment, and antibiotic used, could influence the presence of IE, differences in host-specific environments could account for conflicting observation of IE in the clinic (*41*). Our proposed mechanism to account for IE is dependent on both growth productivity and the initial density of the population. For a given growth productivity, and in a closed system, populations initiated at lower initial density will produce more ATP over a longer period of time as compared to their higher initial density counterparts. As higher initial density populations will enter stationary phase quickly, a concurrent reduction in the concentration of ATP will protect these populations from antibiotics; this leads to a higher MIC. Conversely, populations initiated at lower initial density will produce ATP over a longer period of time before entering stationary phase; this longer period of growth where ATP production is greater than in stationary phase will potentiate antibiotic lethality and reduce MIC. Thus, for a given growth productivity, higher initial density populations have a greater MIC relative to lower initial density populations, thus fulfilling the core definition of IE. When comparing between different growth productivities, the relationship between the concentration of ATP and maximum growth rate determines MIC. For populations with low growth productivity, ATP production decreases with increasing maximum growth rate, resulting in a negative growth productivity (e.g., glucose). In this scenario, the effective reduction of [ATP] at higher log phase maximum growth rates reduces antibiotic lethality. As bacteria initiated from high initial density will have reduced [ATP] owing to both lower growth productivity and earlier entry into stationary phase (as compared to their lower initial density counterparts), they are protected against antibiotics. Thus, ΔMIC is high. As growth productivity increases, bacteria produce higher concentrations of ATP at higher maximum growth rates, thus leading to a positive growth productivity (e.g., acetate). This increase in [ATP] potentiates antibiotic lethality. Thus, for populations initiated from high initial density, even when rapidly approaching stationary phase owing to higher maximum growth rates, increased ATP-driven antibiotic lethality reduces the MIC relative to lower initial density populations. Thus, ΔMIC is reduced. Last, and as supported by our results, if growth productivity is sufficiently high (e.g., acetate and uracil), the MIC of both high and low initial density populations are effectively the same, and ΔMIC is reduced to zero. Our findings generally apply to a closed system where growth and metabolism are bounded by nutrient limitation. It is currently unclear whether growth productivity can account for IE in an open system, such as a chemostat. We note that our estimation of growth productivity in this manuscript has its limitations. For example, we chose to estimate the relationship between the concentration of ATP and maximum growth rate using the slope of a linear line, which will not capture any significant nonlinear trends in this relationship. Despite these approximations, the relationship between ΔMIC and growth productivity remains strong; we have observed this relationship consistently for multiple species of bacteria and carbon source, in addition to conditions that used either casamino acids or equimolar concentrations of amino acids (fig. S17, SM Results). Thus, our simplified approach to estimate growth productivity does not appear to influence the overall interpretation of its relationship to ΔMIC.

While previous work has indicated that the concentration of ATP and maximum growth rate can separately influence antibiotic efficacy, our metric of growth productivity may serve to unify the influence of these features of bacterial physiology, perhaps even in the absence of IE. It is important to consider both the role of ATP and growth rate together when considering IE. If one only considers the concentration of ATP in the absence of changes in growth, entry into stationary phase would not be considered. Without considering entry into stationary phase owing to growth, the concentration of ATP would not be reduced for the high initial density population. Thus, populations initiated from both high and low initial density would have the same concentration of ATP, which would likely result in identical MICs. If one only considers growth in the absence of changes in the concentration of ATP, then populations would have the same concentration of ATP throughout the growth curve. That is, a reduction in ATP would not occur in stationary phase. Thus, the high initial density population, although entering stationary phase earlier than the low initial density population, would have the same concentration of ATP. We would therefore expect the MIC of both populations to be the same. Consideration of both growth rate and metabolism is reflected in our experimental findings; maximum growth rate and [ATP] were not consistently associated with changes in ΔMIC (SM Results, fig. S19). Alternatively, growth productivity was consistently and strongly associated with ΔMIC over a wide range of condition and bacterial species. It is entirely possible that there are reactions and changes to bacterial physiology that are occurring as growth productivity changes, such as changes in flux through the tricarboxylic acid cycle (*42*), nucleotide concentrations (*43*), and reactive oxygen species (*44*), as well as changes to bacterial physiology during stationary phase outside of growth and metabolism. While these additional underlying changes may play an important role in determining IE, we have nevertheless demonstrated conclusive links between growth productivity and ΔMIC; additional changes to bacterial physiology in light of the inoculum certainly warrant future study. Overall, it is important to consider both growth and the concentration of ATP when considering IE.

Using different percentages of glucose, and both uracil and acetate as a carbon source, we reduced ΔMIC to zero for *E. coli.* However, when glucose was used as the carbon source for *A. faecalis* and *P. aeruginosa*, although increasing growth productivity reduced ΔMIC, we were unable to abolish IE. This may reflect differences in carbon source preference. Carbon sources that are preferred by each individual species could result in the formation of more ATP, which could extend the period of time during log phase growth. For example, *P. aeruginosa* is known to prefer organic acids as a carbon source as opposed to complex carbon sources, such as glucose (*38*). As evidence that carbon source preference may be critical to reduce IE across diverse pathogens, we provided *P. aeruginosa* with acetate, an organic acid, as a carbon source. We found that growth productivity increased and that ΔMIC decreased as compared to when glucose was used as the carbon source. Accordingly, to reduce IE in each species of bacteria, different carbon sources, and different percentages of carbon sources, may be required.

In line with recent studies that have examined the use of metabolites as possible antibiotic adjuvants [e.g., (*42*)], modulation of growth productivity using metabolite(s) may reduce or eliminate IE, which could prolong the usefulness of existing antibiotics. For example, previous work has shown that potentiating ATP production through the use of different metabolites can increase susceptibility of persister cells (*45*) and opportunistic pathogens (*42*) to aminoglycosides. It has been proposed that providing antibiotics along with metabolites to serve as adjuvants could increase antibiotic efficacy in the clinic (*46*).

It has been suggested that the emergent properties of microbial populations be studied as mechanisms to drive the evolution of antibiotic resistance, as opposed to the sole focus on the individual bacterium (*6*). As IE in this study occurs in the absence of any unique gene-based resistance mechanism that is specific to any one individual bacterium, it is the emergent properties of a bacterial population that drive the resistance mechanism. Prolonged growth of a bacterial population under sub-MIC conditions, owing to IE, may lead to the evolution of additional resistance mechanisms (*16*). To our knowledge, IE has yet to be observed in nonclinical environments where populations of bacteria reside, including soils and wastewater. However, it is well established that antibiotics have been detected in such areas (*47*) and that the acquisition of infections caused by antibiotic resistance bacteria can occur outside of the clinical setting (*48*). Thus, it is entirely possible that IE serves as a mechanism by which microbial communities can tolerate antibiotics in the natural environment allowing for the evolution of antibiotic resistance.

## MATERIALS AND METHODS

### Strains and growth conditions

*E. coli* strain BW25113 [F-Δ(*araD*-*araB*)*567* Δ*lacZ4*787:*rrnB*-3 λ-*rph-1* Δ(*rhaD*-*rhaB*)*568 hsdR514*] was used throughout. Note that BW25113 contains the *amp*C β-lactamase and several efflux pumps. *P. aeruginosa* strain PA14, *K. pneumoniae* strain (Carolina Biological, Burlington, NC), *A. baumannii* strain 5-109 (Carolina Biological), and *A. faecalis* (Carolina Biological) were used where indicated. Keio collection mutants (*49*) were obtained from Horizon Discovery (Boyertown, PA). Overnight cultures (grown for ~24 hours) were created by isolating single colonies from Luria-Bertani (LB) agar medium (MP Biomedicals, Solon OH). Each colony was subsequently inoculated in 3 ml of liquid LB medium and was shaken overnight (250 RPM and 37°C) in culture tubes (Genesee Scientific, Morrisville, NC). Bacteria grown under this condition had reached stationary phase as we did not observe a change in CFU per milliliter between 23 and 25 hours after inoculation (fig. S2). Experiments were performed in modified M9 medium [1× M9 salts (48 mM Na_2_HPO_4_, 22 mM KH_2_PO_4_, 862 mM NaCl, and 19 mM NH_4_Cl), 0.5% thiamine (Alfa Aesar, Ward Hill, MA), 2 mM MgSO_4_, and 0.1 mM CaCl_2_] with various percentages of glucose (or additional carbon sources) and casamino acids (Teknova, Hollister, CA). For most of the assays, we used casamino acid concentrations of 0.01, 0.05, 0.1, 0.5, and 1.0%. This was reduced to four concentrations (0.05, 0.1, 0.5, and 1.0%) if growth with 0.01% was unreliable or minimal (less than 0.01 at the lowest concentration of antibiotic tested or in antibiotic-free medium). Additional information on key reagents used in this study can be found in SM Methods.

### Maximum growth rate and curve fitting

Overnight cultures were washed once in ddH_2_O and diluted 200-fold into 200 μl of fresh M9 medium, which was subsequently placed in the wells of a 96-well plate. The medium was overlaid with 70 μl of mineral oil to prevent evaporation. OD_600_ was then measured every 10 min for approximately 16 hours in a PerkinElmer Victor X4 (Waltham, MA) plate reader that was set to 37°C. OD_600_ values from cell-free medium were subtracted from all measurements to ensure that only cell density was being recorded. Bacterial growth curves from three biological replicates were averaged. We then extracted maximum growth rate by fitting the growth curves to a logistic equation (*50*), using a modified Gompertz equation or a k-means clustering analysis (as described in SM Methods).

### Determining the concentration of ATP

Overnight cultures were diluted 1/40 into 40 ml of M9 medium with carbon source and that lacked casamino acids. Bacteria were shaken at 250 RPM and at 37°C for 2 hours in a 50-ml conical tube. Cultures were then concentrated 2× in fresh diluted (3:1) M9 medium that contained the carbon source and casamino acids. One hundred microliters of these cultures was added to the wells of an opaque walled 96-well plate, overlaid with two Breath-Easy filters (Sigma-Aldrich, St. Louis, MO, to prevent evaporation), and was shaken at 250 RPM/37°C for 1 hour whereupon they reached OD_600_ = ~0.075. [ATP] was measured using a BacTiter-Glo assay (Promega, Madison, WI) according to the manufacturer’s recommendations. ATP (quantified using luminescence) and OD_600_ were measured in a microplate reader. Using a lower initial density of bacteria (1/400) to measure [ATP] does not affect the concentration of ATP measured (fig. S2—note that these bacteria were concentrated 10× before the measurement of ATP to account for differences in density). A standard curve prepared using pure ATP (ATP disodium salt hydrate, Sigma-Aldrich) was used to quantify the ATP concentration. Additional details on measuring ATP during heat shock, in the presence of protease inhibitor and in stationary phase, can be found in SM Methods.

### Measuring the IE and MIC

Overnight cultures were washed in ddH_2_O and diluted in modified M9 medium that contained various concentration of antibiotics, carbon source, and casamino acids. Two hundred microliters of these cultures was then placed in the wells of a 96-well plate. The plate was overlaid with two Breathe-Easy sealing membranes (to reduce evaporation but ensure an aerobic growth environment) and was then shaken at 250 RPM/37°C for 24 hours (or 40 hours where indicated). Cell density was determined using OD_600_ measured in a microplate reader. All OD_600_ measurements were blanked using OD_600_ values observed in cell-free medium. Any condition where growth did not exceed 0.01 was set to zero (see SM Methods for justification). Concentration gradients for antibiotics used in MIC assays are presented in table S6. For each bacteria and antibiotic pair, we chose the smallest gradient that would lead to an observable difference between high-density and low-density populations and that could be largely contained in a single 96-well plate. We generally used the same concentration gradient for a given antibiotic-bacteria species pair so as to not allow changes in concentration gradient to dictate differences in MIC for the high- and low-density populations. We verified the qualitative trends in our OD_600_ measurements using CFUs by performing the experimental setup above. After 24 hours of growth, we performed a dilution series and determined the number of CFUs growing on approximately 1 ml of LB agar medium housed in 24-well plates. We note that due to the constraints afforded by the carrying capacity of growth medium with low percentages of glucose and casamino acids, we could not test initial densities greater than ~5 × 10^7^ CFU/ml (fig. S6).

### Mathematical modeling

We created a model to describe the density-dependent bistability of survival as a function of initial cell density and antibiotic concentration. Bacteria grow according to logistic growth that is scaled by basal growth rate (μ) and non–biomass-generating metabolism (ε). Antibiotics reduce growth using a per-capita death rate term, which is dependent on the density of the population (*N*), the antibiotic concentration (*A*), and antibiotic lethality (*b*)

$$\frac{dN}{dt}=(\mu -\epsilon )\left(1-\frac{N}{N_{m}}\right)N-\left(\frac{N^{*}A^{*}b}{K+N}\right)$$ (1)

where μ is the basal growth rate (per hour) and ε is metabolism (millimoles per gram per hour), which approximates a maintenance coefficient as described in several previous studies (*33*). We assume that cells grow logistically to a carrying capacity (*N*_m_), *A* represents the antibiotic concentration (unitless), and *b* represents the antibiotic-specific death rate (per hour) as a function of the antibiotic concentration, and that cells are killed according to Michaelis-Menten dynamics, where *K* represents the half-maximal killing rate of the antibiotic. All simulations were performed for *t* = 24 hours using MATLAB (R2021b, MathWorks Inc., Natick, MA) using ode45. Base parameters are shown in table S4. Modifications are as indicated in the corresponding figure legend.

### FBA and whole-genome modeling

FBA was performed using the COBRA toolbox v.3.0 (*51*) coupled with the iJO1366 genome-scale model of *E. coli* metabolism (*52*). Lower and upper bound flux parameters were derived from previous literature (see SM Results, table S5).

### Statistical analysis

Statistical analysis is indicated in the main text or figure legends. Unpaired *t* tests (unequal variance) were performed using Microsoft Excel (Redmond, WA). Standard linear regression analysis was performed using JMP Pro 16 SAS Institute Inc. (Cary, NC). Deming regressions were performed in GraphPad Prism (version 9.3.1, GraphPad, San Diego, CA). SD in MIC values was used for error on the *y* axis; standard error from the linear line used to calculate growth productivity was used as the error in the *x* axis. WLS regressions (which account for error on the *x* axis) were performed in JMP Pro 16. Additional details are provided in SM Methods.

## Supplementary Materials

### This PDF file includes:

Supplementary Text Figs. S1 to S19 Tables S1 to S13 References

## Notes

View/request a protocol for this paper from *Bio-protocol*.
