# Supplementary material for: Growth productivity as a determinant of the inoculum effect for bactericidal antibiotics
Source: Sci Adv. 2022 Dec 14;8(50):eadd0924. doi: 10.1126/sciadv.add0924 (PMC9750144; doi:10.1126/sciadv.add0924)
Supplement: Supplementary file 1 — Supplementary Text Figs. S1 to S19 Tables S1 to S13 References [file sciadv.add0924_sm.pdf]

Science Advances
AAAS

# Supplementary Materials for

## Growth productivity as a determinant of the inoculum effect for bactericidal antibiotics

Gabriela Diaz-Tang *et al.*

Corresponding author: Robert P. Smith, rsmith@nova.edu

*Sci. Adv.* **8**, eadd0924 (2022)
DOI: 10.1126/sciadv.add0924

**This PDF file includes:**

Supplementary Text
Figs. S1 to S19
Tables S1 to S13
References

**Supplementary Text**

**SM Methods**

*Key reagents*

Antibiotics tested were as follows: kanamycin sulphate (Fisher BioReagents, Fisher Scientific, Hampton, NH), streptomycin sulfate (Fisher BioReagents), carbenicillin disodium salt (Alfa Aesar) and ciprofloxacin (Acros Organics, Fair Lawn, NJ). Antibiotics were dissolved in 10 mL of solvent (dd$H_2O$ for all antibiotics except ciprofloxacin where ethanol was used) and used or diluted to the working stock concentrations detailed in Table S6.

Carbon sources tested were as follows: D(+) mannose (Acros Organics), glycerol (Acros Organics), D(+) ribose (Acros Organics), 2- ketoglutaric acid (Alfa Aesar), D(+) galactose (Alfa Aesar), D-lactose (Alfa Aesar), sodium pyruvate (Alfa Aesar), succinic acid (Alfa Aesar), citric acid anhydrous (Fisher Scientific), sorbitol (Fisher Scientific), D-glucose (Fisher Scientific), D(+) trehalose dihydrate (MP Biomedicals), D(+) lactic acid lithium salt (MP Biomedicals), sodium acetate (Sigma Aldrich, St. Louis, MO) sucrose (Sigma-Aldrich), uracil (Sigma-Aldrich), adenine (Sigma-Aldrich) and D(+) raffinose pentahydrate (TCI, Portland, OR). For experiments using equimolar concentrations of amino acids, we used the following at the concentrations indicated in the text: L (+)-Glutamic acid (Acros Organics), L-Isoleucine (Acros Organics), L-Threonine (Acros Organics), L-Leucine (MP Biomedicals), L-Serine (Alfa Aesar), L-Tryptophan (Alfa Aesar), L-Tyrosine (Alfa Aesar), L-Cysteine hydrochloride monohydrate (Alfa Aesar), L-Valine (TCI, Portland, OR), L-Alanine (Sigma), Glycine (Sigma), L-Aspartic acid (Fisher Scientific), L-Arginine (Sigma), L-Histidine (Sigma), DL-Methionine (Fisher), L-Lysine (Fisher Scientific), L-Phenylalanine (Fisher Scientific), L-Proline (Sigma).

*Measuring the change in colony forming units during ATP measurement*

We measured the number of colony forming units (CFU) by performing a dilution series and plating bacteria onto ~1 mL of LB agar in the wells of a 28 well plate. To determine if using undiluted or diluted (3:1) medium affected bacterial density at the time at which [ATP] is measured, initial CFUs were measured from either undiluted or diluted (3:1) M9 medium with 0.04% glucose (and without casamino acids, as per our protocol for measuring [ATP] (*Methods*, main text). After 2 hours of growth, 2X concentration and growth for 1 hour in casamino acids (as per our protocol for measuring [ATP]), we determined the number of CFUs in undiluted and diluted M9 medium containing 0.01, 0.05, 0.1, 0.5 and 1% casamino acids. To show that our measurement time point (3 hours) and bacterial density ($OD_{600}$ = ~0.1) at which [ATP] is measured was lower than carrying capacity, bacteria were allowed to grow for another 21 hours in diluted medium whereupon CFUs were measured.

*Measuring ATP during heat shock and in the presence of protease inhibitor*

We generally followed the same approach to measure [ATP] as described in the *Methods* section with the following changes. To measure [ATP] in the heat shock (42ºC) condition, *E. coli* was grown at 37ºC for 2 hours. Following 2X concentration and the addition of casamino acids in diluted M9 medium, cultures were grown at 42ºC for one hour. [ATP] was then measured. To measure [ATP] in media with 10 ng/mL phenylmethylsulfonyl fluoride (serine protease inhibitor), *E. coli* was grown at 37ºC for 2 hours. Following 2X concentration and the addition of casamino acids in diluted M9 medium, PMSF was added, and cultures were grown for one hour. [ATP] was then measured as described in the main text and in (*21*).

*Measuring ATP in stationary phase*

To measure the concentration of ATP in the stationary phase, we diluted an overnight culture of *E. coli* 1/10 into 30 mL of M9 medium with 0.04% glucose. After 2 hours of shaking at 37ºC (250

RPM), the cells were concentrated two-fold and resuspended in diluted (3:1) M9 medium with 0.04% glucose and different percentages of casamino acids. 100 μL of each cell culture was added to the wells of an opaqued walled microplate, overlaid with 2 Breathe-Easy films and shaken at 37°C (250 RPM) for 4 hours. ATP was then measured as described in the main text and in (*21*).

*Maximum growth rate and curve fitting*

Bacterial growth curves from three biological replicates were averaged. Next, the average was log-transformed and normalized to the initial minimum density. This step helps to remove artifacts of any background effects and ensure all data was initiated at the same starting point, thus reducing the error in our fitting methods. Three distinct methods were used to calculate maximum growth rates over 10 hours of growth. The first method utilized the well-known logistic equation (Eq. S1):

$$y = \frac{A}{\left\{1+\exp\left(\frac{4\mu_m}{A}(\lambda-t)+2\right)\right\}} \qquad \text{(Eq. S1)}$$

The second method utilized the well-known modified Gompertz equation (Eq. S2).

$$y = A\exp\left\{-\exp\left(\left(\frac{\mu_m e}{A}\right)(\lambda-t)+1\right)\right\} \qquad \text{(Eq. S2)}$$

In both equations, $A$ is the maximum density, $\mu_m$ is the maximal growth rate, and $\lambda$ is the lag time. In all cases, we used lower bounds of 0, and upper bounds of 2, 1, and 10 for $A$, $\mu_m$, and $\lambda$ respectively; this ensures that all parameters would be fit within biologically reasonable values. For example, no parameter could take a negative value, which is biologically infeasible, and all parameters would be maintained within a reasonable maximum value based on the general trends in the experimental data. Fitting was performed in MATLAB 2021a using lsqcurvefit, which is a non-linear least squares solver. Specifically, lsqcurvefit takes an initial condition for each parameter, the equation (e.g., Eq. S1 or S2 above), and parameter bounds, and estimates the best values for all three parameters that minimize the distance between the data and fit. For each fit, we determined the average residual. Both of these

two methods rely on analytical expressions of bacterial growth (i.e., the equations 1 and 2 that explicitly describe *y* (cell density) as a function of time) to best estimate the maximum growth rate. Lastly, for the third method, we manually fit the growth rate using a non-arbitrarily identified longest region of fastest growth. Specifically, log-transformed curves were first smoothed to remove small artifacts (e.g., spikes due to plate reader inconsistencies). Then, k-means clustering analysis was used to identify the longest region of fastest growth, consistent with the maximum growth rate. This approach works by first removing any initial lag by identifying the region where the curve begins to increase exponentially, and then separates all remaining data points into two groups. The group with the greatest rate of change over the longest period is taken as the region of continuous exponential growth. Finally, this identified area was fit with a linear line as done previously (*7*).

*Determining the cutoff value of 0.01 to indicate no growth*

After blanking $OD_{600}$ for MIC assays, any condition where growth did not exceed 0.01 was set to zero. We used the 0.01 threshold to guard against fluctuations in $OD_{600}$ values in cell free medium. To establish this value, we measured fluctuations in $OD_{600}$ values in 17 different cell free media containing different percentages of glucose and casamino acids over the course of 24 hours. We found that $OD_{600}$ of cell free medium could fluctuate +/- 0.009 and thus any value below this was not reliable for indicating bacterial growth. We note that not setting values below 0.01 to zero does not alter ΔMIC for bacteria grown in 0.04% glucose (Fig. S2).

*rRNA extraction and quantification*

To isolate total rRNA, the Qiagen RNeasy Mini Kit was used following the RNAprotect Bacteria Reagent Handbook with modifications. *E. coli* was grown as described in our protocol for *Determining the concentration of ATP* (*Methods*, main text). ~175 µL of cell-cultured medium was collected

into 1.5 mL centrifuge tubes and centrifuged for 2 minutes at 12,000 RPM. Pelleted cells were resuspended in 175 μL of diluted (3:1) M9 medium. Each tube was centrifuged again for 2 minutes at 12,000 RPM and the remaining supernatant was discarded. Then, 30 μL of a 10 mg/mL lysozyme solution (MP Biomedicals) in 1X Tris-EDTA (Thermofisher) was added to each tube and incubated at room temperature for 20 minutes while vortexing every 2 minutes. Total rRNA was then extracted according to the manufacturer's recommendations, including the optional in-column DNase digestion using the RNase-Free DNase set. Total rRNA was quantified using a Nanodrop.

*Quantifying $NAD^+$/NADH*

To quantify the concentration of $NAD^+$/NADH, *E. coli* was grown as described in the protocol for *Determining the concentration of ATP* (*Methods*, main text). 50 μL of each cell culture was added to the wells of an opaqued walled microplate. $NAD^+$/NADH was then measured using the $NAD^+$/NADH-Glo Assay (Promega, G9071) according to the manufacturer's recommendations. $NAD^+$/NADH (quantified using luminescence) and $OD_{600}$ were measured in a microplate reader after 40 minutes of incubation at room temperature.

*Quantifying ΔMIC using bacteria in mid-log phase.*

Bacteria were grown overnight as described in the main text (*Strains and growth conditions*). After 24 hours of overnight growth, we diluted bacteria 1000-fold in 2 mL of fresh LB medium in a culture tube. After 3 hours, the bacteria were isolated via centrifugation, washed in $ddH_2O$ and diluted 10 and 100-fold; these served as our high ($1.89 \times 10^7$ +/- $1.51 \times 10^6$ CFU/mL) and low ($2.10 \times 10^6$ +/- $3.21 \times 10^5$ CFU/mL) initial dilutions for this experiment. Bacteria were then grown as described in the main text (*Measuring the inoculum effect and MIC*).

*Measuring pH of the growth medium*

To measure initial pH, we prepared M9 medium (*Methods*, main text) with various carbon sources (0.04%) and five different percentages of casamino acids (0.01, 0.05, 0.1, 0.5 and 1%). For each different carbon source, we took 150 μL of medium containing each percentage of casamino acid, combined the aliquots in a 15 mL conical tube and add 2 mL of $ddH_2O$ to ensure sufficient volume for measurement. pH was then measured while the tube was gently swirled. To measure final pH, we inoculated a 50,000X dilution of an overnight culture of *E. coli* into 200 μL of medium in a 96 well plate, overlaid the plate with two Breathe-Easy membranes and placed the plate at 37°C/250 RPM for 24 hours. As above, we removed 150 μL of medium containing each percentage of casamino acids, pooled the media containing each carbon source in 15 mL conical tubes, and added 2 mL of $ddH_2O$ to increase volume. We then measured the pH with gentle swirling.

*Additional details on statistical analysis*

Statistical analysis was performed as indicated in the text or figure legend. Unpaired t-tests (unequal variance) were performed using Microsoft Excel (Redmond, WA). We performed three different types of linear regressions in this study as specified in the figure legend. For data where error was not present on the x-axis, we performed a standard linear regression analysis using JMP Pro 16 SAS Institute Inc., Cary, NC).

To account for error when estimating growth productivity, we performed two error-in-variable analyses to account for this error; a Deming regression (*53*) and a weighted least squares (WLS) linear regression (*54*). A Deming regression is an error-in-variables regression that considers error on both the x and y axis. Thus, the average error for both growth productivity (standard error, which accounts for error in the slope used to calculate growth productivity) and MIC (standard deviation) was considered. Error was calculated by determining the standard deviation of the average

values plotted on the x and y-axes. We note that this extra step makes a Deming regression different from an orthogonal linear regression and also accounts for the fact that both the y and x values are in different units. Deming regressions were performed in GraphPad Prism (version 9.3.1, GraphPad, San Diego, CA).

WLS is also an error-in-variables regression that weights the values along the x-axis by their error; values with greater errors have less influence on the regression as compared to values that have smaller errors. Thus, if values with large errors were confounding our analysis, this method detects and controls for this. For this analysis, we converted the standard error from each growth productivity value into variance; in accordance with the WLS protocol, we weighted each value as using the reciprocal of the variance. WLS was performed in JMP Pro 16.

To test the significance of the slope for the relationship between $\Delta MIC_{kan}$ and growth productivity (Fig. 7C), we also used a bootstrapping approach using a simple linear regression to resample the data. We generated 2,500 bootstrapped slopes from our data and tested the null hypothesis that our observed slopes were significantly different from zero using a two-tailed t-test in JMP Pro 16. All additional tests were performed in JMP Pro 16 (SAS Institute Inc., Cary, NC).

Values for all linear regression where growth productivity was calculated, including P values, $R^2$ and 95% confidence intervals are found in Tables S7. Values for all simple linear regressions are found in Table S9. Values for Deming regressions are found in Table S10. We note that Deming regressions do not calculate $R^2$ values. Values for all WLS regressions are found in Table S11. Values from bootstrapping analysis are found in Table S9. Unless otherwise indicated, $R^2$ and P values shown on each figure panel where a regression was used are from a simple linear regression.

**SM Results**

*Antibiotic classes where IE has been observed*

IE had been observed previously in multiple bactericidal antibiotics including β-lactams (*55*), aminoglycosides (*14*), cephalosporins (*55*), carbapenems (*56*), fluoroquinolones (*19*), glycopeptides (*57*), and cyclic lipopeptides (*58*). This demonstrates that, in conjunction with the clinical studies and case reports shown in Table S1 and S2, IE is a wide ranging mechanism by which bacteria resist antibiotics.

*Existing mechanisms to explain IE*

Several mechanisms to explain IE have been proposed. These include a reduced ratio of antibiotics to drug binding targets (*18*), recovery of growth following antibiotic treatment (*14*), prolonged lag phase at high bacterial density (*17*), and growth/no growth boundaries determined by bacterial density and environmental conditions (*59*). In addition, several antibiotic specific mechanisms have been proposed to account for IE. These include antibiotic induced degradation of the antibiotic target (*14*), collective degradation of antibiotic (*18*), and differential growth rates (*19*). However, some of these hypotheses lack experimental verification, and in general, cannot account for IE across antibiotic classes. They also do not take into account any measures of energy status in bacteria, including the concentration of ATP, which better predicts antibiotic efficacy as compared with growth (*21*).

*The use of casamino acids as a nitrogen source*

Previous work has shown that the use of different ratios of carbon and nitrogen sources, such as glucose and casamino acids, can alter the efficiency at which bacteria utilize nutrients (*60, 61*). We used casamino acids as a nitrogen source as amino acid biosynthesis requires a substantial amount of

cellular energy (*39, 62*); by providing a complex source of amino acids, we would alleviate the energy required to synthesize amino acids. This, in turn, would allow more ATP produced from a carbon source to be directed towards biomass generation (growth rate) (*62, 63*). Overall, by varying the concentration of casamino acids, we could vary growth rate (thus allowing us to quantify growth productivity) while allowing a higher amount of ATP generated from carbon source consumption to be available for other cellular processes, including reactions that potentiate antibiotic lethality. Indeed, previous work has established that using a carbon source coupled with varying amounts of casamino acids can manipulate ATP concentrations, growth rate, and antibiotic lethality (*21*). We note that use of casamino acids, as opposed to equimolar concentrations of amino acids, does not appear to confound our results. Specifically, when using equimolar concentrations of amino acids, we continue to observe a significant linear relationship between $\Delta MIC_{kan}$ and growth productivity; if growth productivity was sufficiently high, $\Delta MIC_{kan}$ was not statistically different than zero (Fig. S17). Moreover, if the sequential and preferentially catabolism of amino acids provided in different concentrations in casamino acids was driving the relationship between growth productivity and ΔMIC, then we would expect, for a given percentage of casamino acids and across different carbon sources, the concentration of ATP would be the same. This is not the case, as there are significant differences in the concentration of ATP when growth conditions containing 0.05% and 1% casamino acids are compared (ANOVA, $P < 0.0001$, Fig. S17). Finally, if casamino acids were the predominant driver of ΔMIC, then we would once again observe that, for a given percentage of casamino acids, the ΔMIC values were the same amongst all carbon sources. We indeed do not observe this (Fig. S17). While the consumption of amino acids is likely having an impact on [ATP], growth rate, growth productivity and ΔMIC, it is not the primary driver of our trends.

*Measurement of ATP*

Previous work has demonstrated that the measurement of ATP using our approach significantly correlates with alternative measures of energy status in bacteria, including $NAD^+$/NADH and oxygen consumption rate (*21*). Moreover, the use of diluted M9 medium (or non-diluted M9 medium) has no effect on the change in CFU during the time over which ATP is measured (Fig. S2).

Although ATP production can decrease when cell density is sufficiently high (i.e., during stationary phase), the point in time (3 hours) at which we measure ATP concentration is well below the carrying capacity of M9 medium (Fig. S2). We confirmed that the concentration of ATP is reduced during stationary phase (Fig. S2). We found that the concentration of ATP measured during log phase (3 hours of growth) is significantly higher than that measured during stationary phase. We confirmed this across different percentages of casamino acids (glucose % = 0.04%, Fig. S2). Thus, a reduction in ATP owing to entry into stationary phase is not affecting our measurements of ATP.

Furthermore, as the concentration of ATP may change during log phase growth, we quantified ATP at 3 hours, 5 hours and 7 hours. For the most part, we did not observe a significant change in the concentration of ATP, with the exception of conditions containing higher concentrations of casamino acids at 7 hours (as compared to the 3 hours); the reduction in [ATP] at this time point may reflect entry into stationary phase (Fig. S2 (glucose) and Fig. S14 (acetate)). We also note that the general trends in growth productivity remain the same between bacteria grown in medium with either glucose or acetate when later times (5 hours, 7 hours) for ATP measurements are considered (Fig. S14). Overall, it does not appear that measuring [ATP] at 3 hours is confounding our results or estimation of growth productivity.

*Determining growth productivity using a linear line*

We used a linear regression to approximate and simplify our estimate of growth productivity for a given carbon source. Previous work has demonstrated that the relationship between measures of metabolism (*60*), including the concentration of ATP (*21*), may not be strictly and linearly correlated to growth rate. Instead, they depend upon the efficiency of resource utilization and the amount of both carbon and nitrogen source (*61, 64*), which may lead to non-linear changes in [ATP] and growth rate. However, so that we could compare growth productivity across diverse growth environments as determined by the identity and percentage of carbon source without having to estimate growth productivity using multiple and potentially disparate fitting approaches, we approximated the relationship between [ATP] and growth rate as linear. This does not appear to confound our results as including the standard error of the slope used to estimate growth productivity in regression analysis (using a Deming or WLS) continues to indicate a significant relationship between ΔMIC and growth productivity (see Tables S10 and S11 for P values, see *SM Methods* for explanation of how Deming and WLS regressions factor in error during a regression).

*Alternative hypotheses to changes in ΔMIC*

<u>Previous findings:</u> We previously ruled out several alternative hypotheses that could potentially account for IE. These include molecular titration of the antibiotic, the role of bacterial metabolites, including $H_2S$ and indole, persister cells formation, cell-cell communication and the formation of antibiotic resistant mutants (*14*).

<u>Efflux pumps:</u> To ensure that transporters were not playing a critical role in the relationship between growth productivity and ΔMIC, we obtained four mutants that lack efflux pumps *(ΔacrA*, *ΔacrB*, *ΔtolC* and *ΔmdtA*) from the Keio collection (*49*). We then determined ΔMIC of streptomycin relative to the wildtype strain. Note that Keio collection strains are kanamycin resistant, thus we performed these experiments with an alternative aminoglycoside, streptomycin. In all cases, there was

not a significant difference in ΔMIC between the wildtype and mutant strains (Fig. S7). This demonstrates that these efflux pumps are not playing a critical role in determining ΔMIC.

Presence of *amp*C β-lactamase in BW25113: To rule out the involvement of the *ampC* β-lactamase in determining ΔMIC for carbenicillin, we obtained an Δ*amp*C knockout from the Keio collection. We challenged this strain with carbenicillin using two different concentrations of glucose. 0.04% and 0.0004% glucose. In the wildtype strain, these percentages of glucose resulted in the greatest and smallest values of ΔMIC for carbenicillin. We observed that the removal of *ampC* did not result in a difference in ΔMIC for both percentages of glucose tested, that ΔMIC continued to decrease as a function of growth productivity measured using the wildtype strain, and if growth productivity was sufficiently high (0.0004% glucose), ΔMIC was no different than zero (Fig. S7). Accordingly, the *ampC* β-lactamase was not the leading determinant of trends ΔMIC for carbenicillin.

Quorum sensing: We acquired a Δ*luxS* mutant strain of *E. coli* that lacks the ability to perform quorum sensing. Using this knockout strain, we quantified ΔMIC of streptomycin. We found that ΔMIC of this strain was the same as the wildtype strain. Thus, quorum sensing cannot account for the value of ΔMIC and IE in our system (Fig. S7). This finding is consistent with previous work showing that quorum sensing, or other secreted metabolites produced during growth, cannot account for IE (*14*).

Number of antibiotic targets: To test the ability of the number of antibiotic targets to account for changes in ΔMIC, we extracted total RNA from *E. coli* under the same experimental conditions where [ATP] was measured. Total RNA can be used as a measure of the total amount of rRNA in the cell because only ~2% of total RNA is attributed to mRNA (*65*) and tRNA is routinely removed during RNA extraction according to the manufacturer. We also note that the production of rRNA and ribosomal proteins is tightly regulated (*66, 67*). Thus, rRNA can serve as a surrogate of total

ribosomes. We quantified total RNA using a nanodrop and plotted ΔMIC as a function of average total RNA for each percentage of glucose measured. We did not find a significant association between the total amount of RNA and ΔMIC for the ribosome targeting antibiotics kanamycin and streptomycin (Fig. S7). This indicates that the total number of antibiotic targets cannot account for ΔMIC .

Changes in uptake rate of antibiotics: Previous work has found that providing different carbon sources or metabolites can increase the accumulation of aminoglycosides via changes in proton motive force (*42, 45, 46, 68*). However, this was not the case for β-lactams nor fluoroquinolones suggesting that this phenomenon is aminoglycoside specific (*42*). Thus, changes in antibiotic uptake cannot account for changes in ΔMIC for carbenicillin and ciprofloxacin when we perturbed growth productivity using different carbon sources (Fig. 7 and 8). In the case of the aminoglycosides kanamycin, we demonstrated in Figs. 2 and 6 that even when using the same carbon source (glucose), increasing growth productivity reduces ΔMIC. Thus, even in instances where we do not change carbon source, we continue to see a strong relationship between growth productivity and ΔMIC. Changes in aminoglycoside uptake owing to different carbons sources are most pronounced in the first four hours of exposure to aminoglycosides (*42*). Because we measure MIC and ΔMIC after 24 hours of growth, it is highly unlikely that small changes in uptake can account for changes in ΔMIC. While we cannot conclusively rule out that changes in the uptake of aminoglycosides are occurring in our experiments with different carbon sources (Fig. 7 and 8), it is likely that they are playing a minor role.

Metabolism (ATP) independently of maximum growth rate: Previous work has indicated that, when decoupled from growth, increasing the concentration of ATP can increase antibiotic efficacy (*21*). We found that increasing the concentration of ATP was associated with a decrease in ΔMIC for *E. coli* when grown in glucose for all antibiotics tested (Fig. S19). However, we did not find significant

associations between the concentration of ATP and ΔMIC for *P. aeruginosa*, *A. faecalis* (Fig. S10), and when all bacterial species were plotted together (Fig. S10). We found a small ($R^2 = 0.28$), yet significant ($P = 0.02$), relationship between [ATP] and *E. coli* grown in all carbon sources tested (Fig. S19). We also note that [ATP] was not consistently and significantly associated with ΔMIC when all individual [ATP] measurements were compared to individual ΔMIC values (Fig. S19). Thus, the most consistent predictor of IE across growth conditions and bacterial species is growth productivity.

<u>Maximum growth rate independent of metabolism</u>: Previous work has indicated that increasing growth rate can potentiate antibiotic lethality (*19, 69*). While we did find significant associations between maximum growth rate and ΔMIC when bacteria were grown in glucose (Fig. S19), we did not find significant associations between the maximum growth rate and ΔMIC for *P. aeruginosa* and *A. faecalis* (Fig. S10), and when all bacterial species were plotted together (Fig. S10). When *E. coli* was grown in different carbon sources (0.04%), we did not find a significant relationship between maximum growth rate and ΔMIC (Fig. S19). We also plotted ΔMIC at each combination of percent glucose and casamino acids. As above, we did not consistently find a strong nor significant association between ΔMIC and maximum growth rate. This was true when maximum growth rate for each ΔMIC value was considered (Fig. S19). Even in scenarios where maximum growth rate was significantly associated with ΔMIC, stronger (greater $R^2$) and more significant (lower P values) were found between [ATP] and ΔMIC (Fig. S19). Finally, we also found that some of the mutants used in Fig. 4 and 5 had insignificant increases in maximum growth rate, while they had significant increases in [ATP]. This significant increase in [ATP] was sufficient to perturb ΔMIC independent of significant changes in growth rate. Thus, maximum growth rate alone cannot explain IE across these diverse conditions.

Carrying capacity: For the same initial density, increasing carrying capacity would afford a longer period of growth. This would increase antibiotic lethality owing to a longer time period over which ATP production is high. This could potentially reduce the difference in total time over which the high and low initial density populations produce high concentrations of ATP, which could effectively reduce ΔMIC. Thus, increasing carrying capacity could account for IE. We therefore plotted carrying capacity of *E. coli* grown in medium with different carbon sources (Fig. 7C) and without antibiotics as a function of ΔMIC. We found a weak ($R^2 = 0.31$), but significant, relationship ($P = 0.018$, simple linear regression, Fig. S19). However, given the strength of the relationship between growth productivity and ΔMIC presented in both our FBA and our experiments (Fig. 7 and 8), growth productivity is the dominating mechanism for IE in our system.

Change in pH: We measured the initial pH of M9 medium containing the various carbon sources used in our study (Fig. 7C) and the final pH after 24 hours of growth (without antibiotics). We did not find a significant association between the initial ($R^2 = 0.016$, $P = 0.61$, simple linear regression) or final ($R^2 = 0.24$, $P = 0.09$, simple linear regression) pH and ΔMIC (Fig. S19).

*Parameter estimation – ordinary differential equation*

We used our model to gain a generalized insight into the relationship between growth and metabolism, the latter of which was measured using [ATP] in our experiments. Accordingly, we estimated the relative order of magnitude of key parameters. We estimated the maximum value of growth rate ($\mu$) by calculating the average growth rate of *E. coli* in M9 medium with 0.4 - 0.00004% glucose and 0.01-1.0% casamino acids (0.38/hr ± 0.09). Our value also approximates additional average growth rates observed in our experiments (*A. baumannii* = 0.29/hr ± 0.16, *A. faecalis* = 0.12/hr ± 0.04, *P. aeruginosa* = 0.38/hr ± 0.05, and *K. pneumoniae* 0.52/hr ± 0.06). We estimated the initial

densities of the high and low initial density by determining the carrying capacity of M9 medium with 0.4% glucose with 0.1% and 1% casamino acids ($1.55 \times 10^9 \pm 5.00 \times 10^8$ CFU/mL). We set this density to 1 (carrying capacity, $N_m$) and normalized the initial high ($N_{high}$, $1.13 \times 10^6$ CFU/mL $\pm$ $2.05 \times 10^5$) and low ($N_{low}$, $1.23 \times 10^5$ CFU/mL $\pm$ $4.99 \times 10^4$) density populations. Thus, we estimated $N_{high}$ and $N_{low}$ values of ~0.05-0.001 and ~0.0005-0.00001, respectively. The order of magnitude of metabolism ($\varepsilon$) was estimated using previously reported maintenance coefficients. That is, the metabolism term represents energy that is not allocated to growth and the generation of biomass. Reported maintenance coefficients for *E. coli* range from 0.013 to 0.473 mmol/g/hr depending upon growth conditions, including carbon source (e.g. (*70*)). Thus, we estimated that $\varepsilon$ would reasonably range between these values. Antibiotic killing rate ($b$) was estimated according to time-kill curves from (*21*), which demonstrated that ~ 1 - 0.1% of a bacterial population survives after 3 hours of growth in an antibiotic concentration greater than MIC. Finally, we fit the half maximal killing rate of the antibiotic ($K$) to our data. All simulations were performed until $t = 24$ to coincide with the timing of our experiments. Sensitivity analysis showing that our modeling predictions remain qualitatively consistent over a range of parameter values is presented in Fig. S8. Using different initial values of high and low initial ($N_{high}$ and $N_{low}$) bacterial density and extending our simulation time to $t = 100$ does not affect the qualitative nature of our predictions (Fig. S8).

*Parameter estimation – flux balance analysis (FBA)*

Flux balance analysis was performed using the COBRA toolbox v.3.0 (*51*) coupled with the iJO1366 genome-scale model of *E. coli* metabolism (*52*). To capture the composition of M9 medium (regardless of the carbon source or percentage of casamino acids in medium), exchange values for $K^+$, $Mg^{2+}$, $K^+$, $Na^+$, $NH_4^+$, $Cl^-$ $P_i$, $SO_4^{2-}$, and $Ca^{2+}$ were set at -1000 and 1000 for the lower and upper

bound, respectively. We estimated the consumption rate of thiamine from previously published literature (*71*). All additional upper and lower bounds commonly associated with the core function of the iJO1366 model were not adjusted unless indicated otherwise.

The lower bound exchange flux for each carbon source tested was derived from literature (see Table S5). Reported consumptions/uptake rates were converted/approximated to mmol/$g_{dw}$/hr and used as the lower bound exchange flux. When extrapolating $g_{DW}$ from $OD_{600}$, we approximated 1 $OD_{600}$ = 0.39g/L cell dry weight (*72*). Where possible, we used consumption rates that corresponded to *E. coli* B strains grown in minimal medium. All upper bound exchange fluxes were set at 1000. To demonstrate the robustness of our FBA predictions, we performed a sensitivity analysis of all carbon source lower bound exchange values by varying each value 5-fold in both directions away from values reported in Table S5 (Fig. S11).

The lower bound exchange flux for amino acids was estimated using data from (*73*), which reports experimentally determined uptake rates of amino acids (from casamino acids at final percentages of 0.2%, 0.1% and 0.05%) in M9 minimal medium. To approximate these uptake rates to our experimental setup (which uses casamino acid concentrations of 0.01%, 0.05%, 0.1%, 0.5% and 1%), and to account for any non-linearity in uptake rates when the percentage of casamino acids is increased or decreased, we averaged the uptake rates from 0.2%, 0.1% and 0.05% casamino acids reported in (*73*). To demonstrate that this approximation did not confound our results, we performed our FBA analysis using the maximum and miniumum uptake values as determined by the standard deviation of the average the 0.2%, 0.1% and 0.05% conditions (Fig. S11). In all cases, all upper bound exchange fluxes were set at 1000.

As oxygen consumption rate has been observed to change with different carbon sources (*74*), we averaged previously reported $O_2$ consumption values for various carbon sources (*74*) used in this study (20.9 +/- 7.8 mmol/$g_{dw}$/hr). The upper bound exchange flux of $O_2$ was set at 1000.

*Relationship between growth productivity and ciprofloxacin*

We observed that increasing growth productivity had a more pronounced effect on aminoglycosides and carbenicillin (β-lactams), as compared to ciprofloxacin (fluoroquinolones, Fig. 2C and 8). While this may be owing to differences in mode of action, it may also be owing to previous findings that suggest a high oxygenation rate is required to sensitize high density populations of bacteria to fluoroquinolones (*19*). Towards the former, it has been previously found that the activity of DNA gyrase is primarily focused on restoring DNA supercoiling during stationary phase growth. Thus, not only does the reduction in [ATP] owing to stationary phase reduce the indirect effects of antibiotic lethality (*44*), but it is likely that the direct mode of action of ciprofloxacin is also limited as DNA gyrase is not focused on DNA replication. Towards the latter, it is possible that due to oxygen limitation in the growth media, the indirect effects of ciprofloxacin are reduced owing to a reduction in aerobic cellular respiration (*37*). While our use of oxygen permeable membranes during growth ensured oxygenation, it is possible that increasing the oxygenation rate of the growth media could result in a more pronounced effect of increasing growth productivity on the decrease in ΔMIC observed for ciprofloxacin. While the effect of increased oxygenation on ΔMIC of ciprofloxacin should be explored in the future, we have nevertheless demonstrated a strong relationship between ΔMIC for ciprofloxacin and growth productivity.

*Using equimolar amino acids to determine growth productivity*

We found that the relationship between $\Delta MIC_{kan}$ and growth productivity remained consistent when we used equimolar concentrations of amino acids; as growth productivity increased, $\Delta MIC_{kan}$ decreased (Fig. S17). While the overall trend remained the same, the values of $\Delta MIC_{kan}$ for some carbon sources tested were reduced as compared to when casamino acids were used in the growth

medium. This difference is likely owing to interactions between the availability of carbon source and specific concentrations of amino acids as a nitrogen source. Indeed, previous work has demonstrated that ratios of carbon source and nitrogen source affect nutrient utilization (*61*), and that there are preferences in amino acid catabolism order in bacteria (*73*). These likely influence growth productivity, and thus ΔMIC. Previous work has indicated that supplementation of medium with different amino acids can potentiate antibiotic lethality by altering cellular respiration (*75*). While some of the values of $\Delta MIC_{kan}$ changed using equimolar concentrations, the relationship between $\Delta MIC_{kan}$ and growth productivity remains intact; this demonstrates that our use of casamino acids does not confound our findings.

# SM Figures and Figure Legends

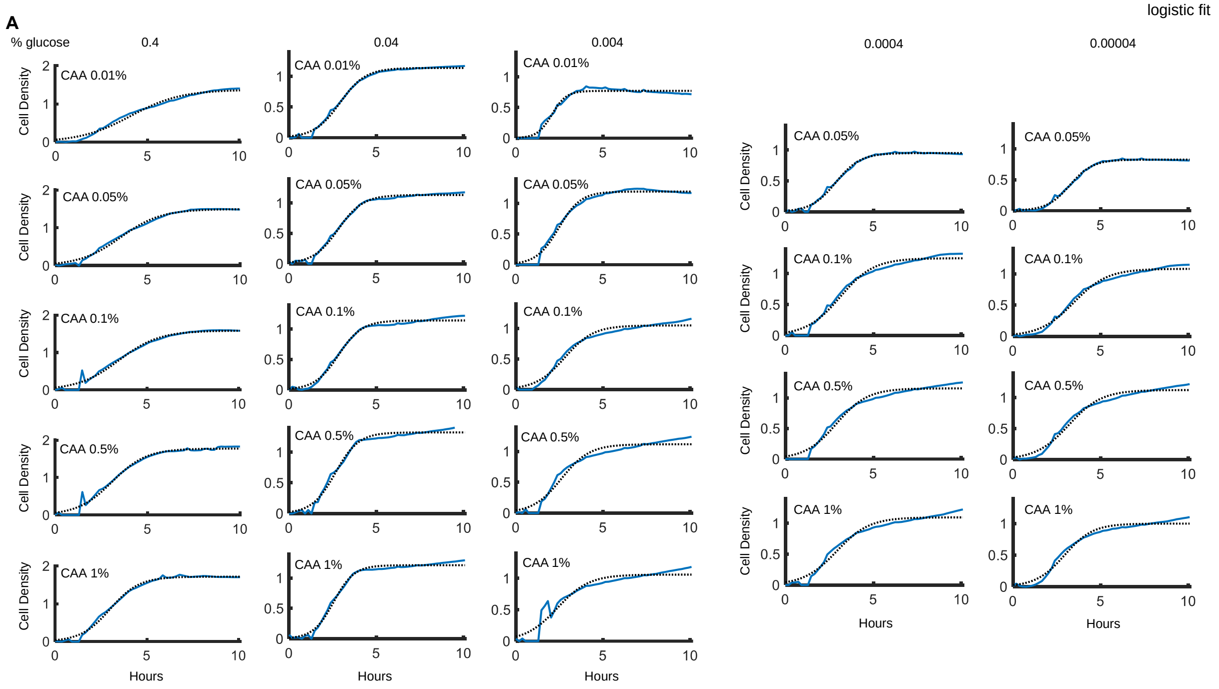


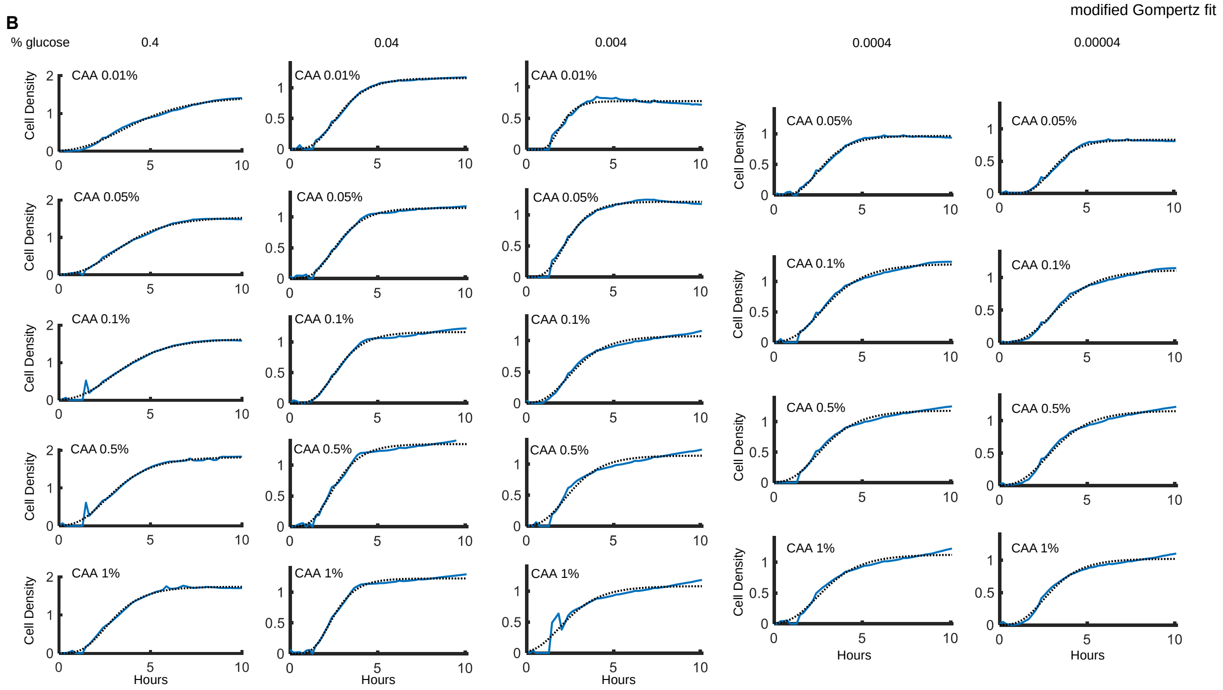

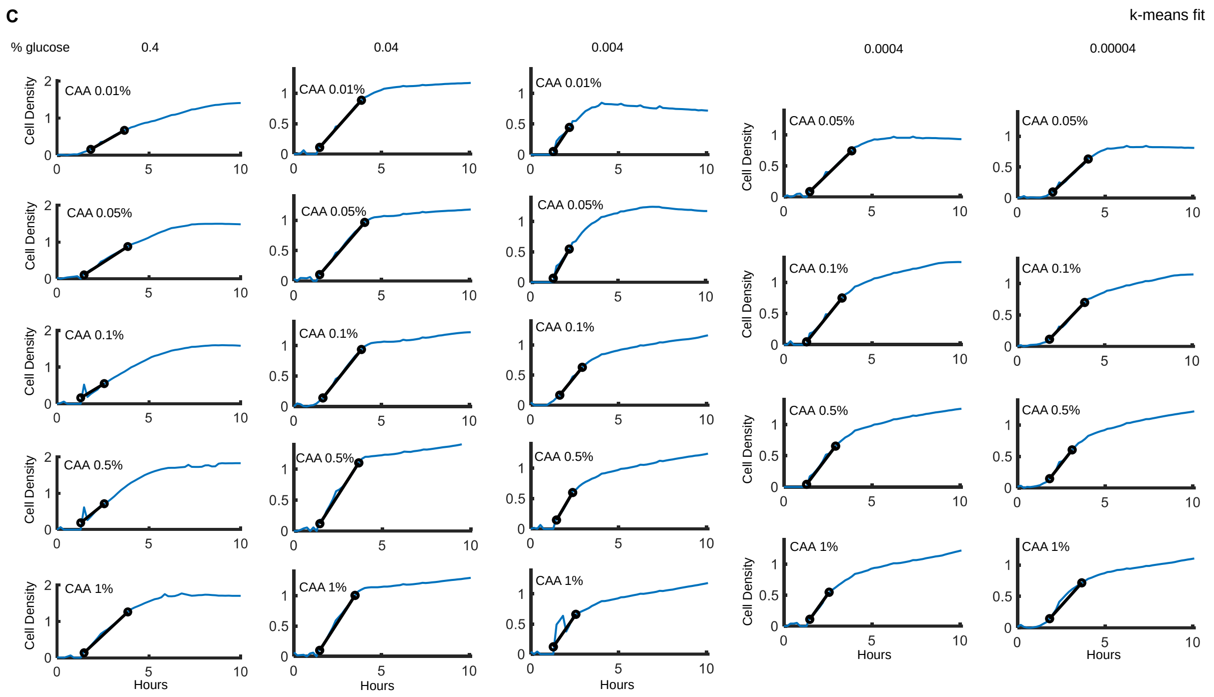


**Fig. S1. Growth curves of *E. coli* grown in M9 medium with different percentages of glucose and casamino acids.**

**A)** Growth curves fit using a logistic equation (Eq. S1). For panels solid line = experimental data and dotted lined = fit using equation. To determine cell density, $OD_{600}$ values were log-transformed and normalized to the initial minimum density. Average plotted from a minimum of three biological replicates. Average residual values for each growth curve fit (logistic and modified Gompertz) are found in Table S3. CAA = casamino acids. For all sub panels y = cell density, x = time in hours.

**B)** Growth curves fit using a modified Gompertz equation (Eq. S2).

**C)** Growth curves fit using a k-means clustering analysis. Solid black line indicates longest region of fastest growth. Black circles indicate beginning and end point.

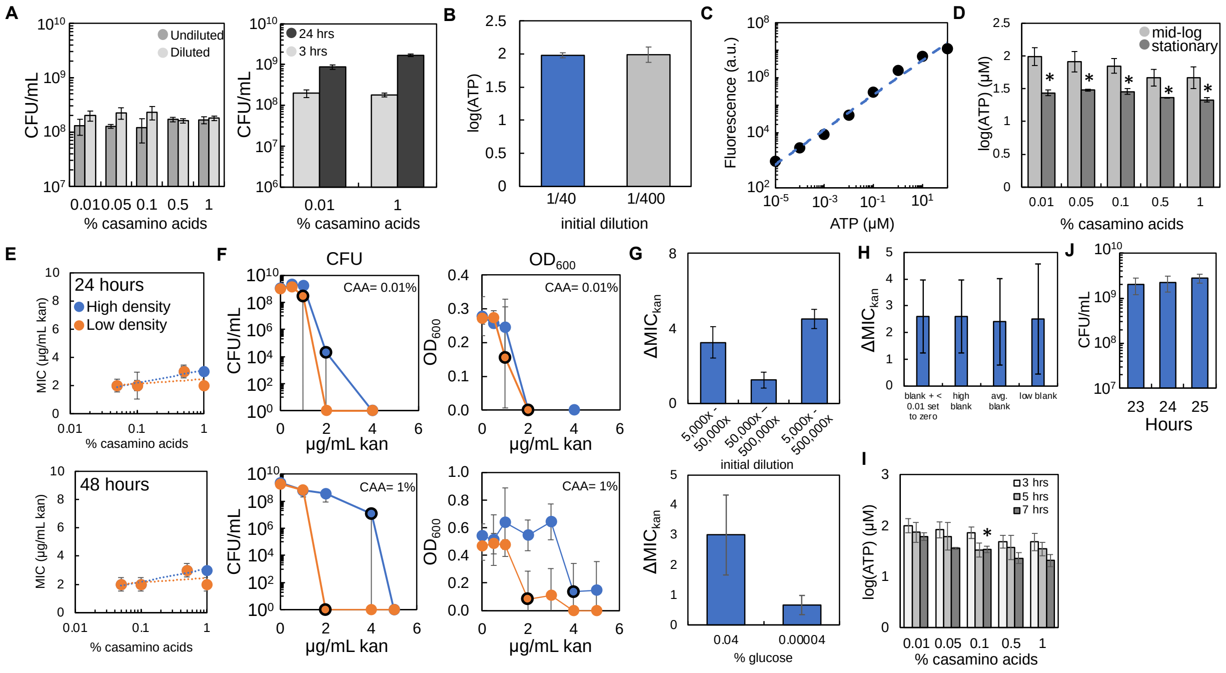


**Fig. S2. Measuring the concentration of ATP and the strength of IE (ΔMIC) in *E. coli.***

**A)** Left panel: Density of bacteria grown in undiluted or diluted (3:1, 0.04% glucose) M9 medium after 3 hours of growth in increasing percentages of casamino acids. There was no difference in CFU/mL when bacteria were grown in undiluted and diluted medium when each percentage of casamino acids was considered (P = 0.18, 0.11, 0.14, 0.55, and 0.57 for 0.01%, 0.05%, 0.1%, 0.5% and 1% casamino acids, respectively, two-tailed t-tests). Right panel: Density of bacteria after 3 and 24 hours of growth (glucose = 0.04%). The bacterial density at 3 hours was significantly below the carrying capacity of the medium (24 hour density, P = 0.007 and 0.002 for 0.01% and 1%, respectively, two-tailed t-tests). This indicated that the concentration of ATP was not reduced owing to entry into stationary phase at the point in time where we measured ATP. For both panels, standard deviation from three biological replicates.

**B)** A lower density of bacteria (1/400 dilution) does not affect the overall concentration of ATP extracted from bacteria. Blue bar = 1/40 dilution of an overnight strain (used for all other

ATP measurements in this manuscript). Grey bar = 1/400 dilution of an overnight strain. Glucose = 0.04%, % casamino acids = 0.01%. We chose to measure 0.01% as it has the lowest carrying capacity of all percentages of glucose. Thus, any changes in the concentration of ATP owing to entry into stationary would be detected in this sample. P = 0.93, two-tailed t-test. Standard deviation from a minimum of four biological replicates, each consisting of three technical replicates.

**C)** Standard curve for the quantification of [ATP]. Purified ATP was measured using a BacTiter-Glo assay kit according to manufacturer's recommended protocol. Standard deviation from three biological samples (error bars are within each marker).

**D)** Average concentration of ATP in *E. coli* grown in M9 medium with 0.04% glucose in mid-log or stationary. Standard deviation from four biological replicates, each measured using three technical replicates. * indicates significantly reduced concentration of ATP within each percentage of casamino acids (P = 0.0007, 0.01, 0.001, 0.018 and 0.012 for 0.01%, 0.05%, 0.1%, 0.5% and 1% casamino acids, one-tailed t-tests).

**E)** Twenty-four hours of growth is sufficient to measure MIC. We determined MIC for initial high- and low-density populations. We used a low percentage of glucose 0.00004% as it had the lowest average maximum growth rate of all percentages of glucose measured (0.3/hr, measured using a logistic equation). We observed that the MIC for both population across percentages of casamino acids was the same at 24 hours, as it was at 40 hours. Standard deviation from a minimum of three biological replicates. Lines indicate general trend in the data.

**F)** Similar trends in growth and $MIC_{kan}$ are found when cell density is measured using colony forming units (CFU, left) and $OD_{600}$ (right). Glucose= 0.04%. Percentage of casamino acids indicated on panels. Data point with a black outline indicates that the cell density was not

different than zero. For CFU measurements, P = 0.32, 0.12, 0.12 and 1 for 0.01%- high density, 0.01%-low density, 1%-high density, and 1%-low density, respectively, Mann Whitney, (Shapiro-Wilk P < 0.0002 for both 0.01% and 1% casamino acids data sets). For $OD_{600}$ measurements, P = 1, 0.053, 0.103 and 0.18 for 0.01%- high density, 0.01%-low density, 1%-high density, and 1%-low density, respectively, one-tailed t-tests. Standard deviation from a minimum of three biological replicates. kan = kanamycin.

**G)** A further reduction in the initial bacterial density continues to show the general trend in $\Delta MIC_{kan}$. Top panel: We determined $\Delta MIC_{kan}$ using a lower initial density condition (1/500,000) in medium containing 0.04% glucose. $\Delta MIC_{kan}$ was greater than zero for all comparison thus indicating that IE is present at this lower initial density. Bottom panel: We determined that reducing the percentage of glucose in the medium (increasing growth productivity) reduced $\Delta MIC_{kan}$ when a lower initial density was included (1/500,000). For each percentage of glucose, $\Delta MIC_{kan}$ was calculated as the average $\Delta MIC_{kan}$ between all comparisons (5,000X-50,000X, 50,000X-500,000X, 5,000X-500,000X). In both panels, standard deviation from a minimum of five biological replicates drawn from four percentages of casamino acids. 1/500,000X = $8.3 \times 10^3$ CFU/mL ± $4.32 \times 10^3$.

**H)** Changing our methods of 'blanking' the medium does not change $\Delta MIC_{kan}$ for bacteria grown in medium with 0.04% glucose. Our standard for determining MIC was to blank $OD_{600}$ values using cell free medium and then to set $OD_{600}$ values of < 0.01 to zero. To demonstrate that this method of treating the data does not fundamentally impact ΔMIC, we compared four different blanking methods using data for *E. coli* grown in 0.04% glucose (with 0.01%, 0.05%, 0.1%, 0.5% and 1.0% casamino acids); average blank was subtracted from all values and values below 0.01 set to zero (blank + < 0.01 set to zero in figure axis), average blank value subtracted

from all values and without any further modifications ('avg. blank' in figure axis), average blank value plus standard deviation of all blank values (subtracted from all values, no further modification, called 'high blank' in figure axis) and average blank value minus standard deviation (subtracted from all values, no further modification, 'low blank' in figure axis). We found no statistical difference in the ΔMIC values obtained using these different blanking methods ($P = 0.9936$, Kruskal Wallis, $P = 0.0057$ Shapiro Wilk for normality). Standard deviation from a minimum of four biological replicates.

**I)** The concentration of ATP during log phase growth. We measured the concentration of ATP in *E. coli* grown in medium with 0.04% glucose after 3 hours (standard procedure for measuring [ATP] in this manuscript), 5 hours and 7 hours. We did not observe a significant difference in ATP concentrations at these different time points with the exception of 3 hours vs. 7 hours for medium containing 0.1% (*, $P = 0.036$, two-tailed t-test). The reduction at this time point may reflect a reduction in [ATP] owing to entry into stationary phase.

**J)** Bacterial density in LB medium from which the majority of experiments were initiated. Experiments were initiated from bacteria that had been grown for 24 hours in LB, thus reaching stationary. To verify this, we measured CFU from overnight cultures that had been grown for 23, 24 or 25 hours. We did not observe a change in CFUs at these three times point indicating that bacteria had reached stationary phase. Standard deviation from three biological replicates. $P > 0.33$ for all comparisons (two-tailed t-test).

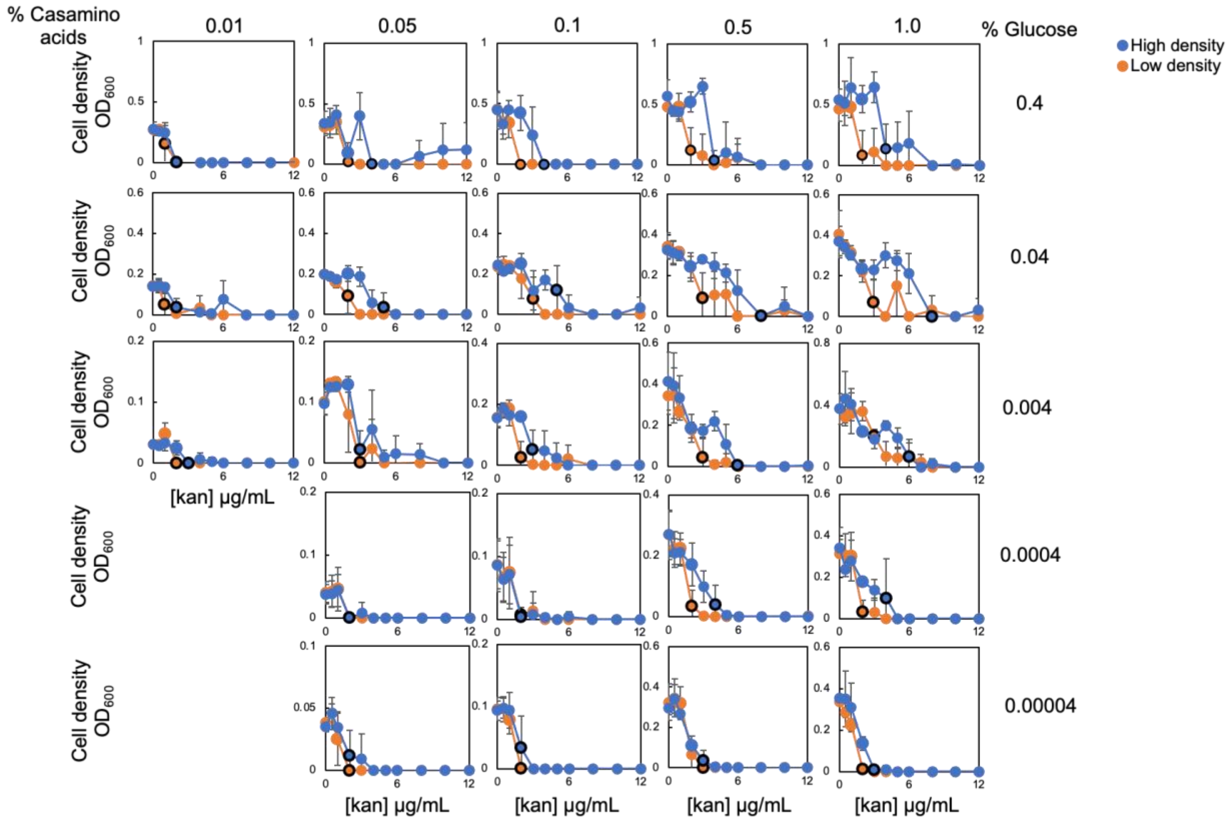


**Fig. S3. Raw growth data for *E. coli* treated with kanamycin.** This data was used to generate the MICs shown in Fig. 2A. Dark outline indicates MIC of each condition as determined using a student one-tailed t-test tested against zero. Standard deviation from a minimum of three biological replicates. As MIC is approached for both high and low initial densities, the variation (as indicated through increased standard deviation) increases. This is likely owing to small stochastic fluctuations in the system. We (*14, 76*), and others (e.g., (*77*)), have previously observed increased variability in cell growth (or death) before or at the MIC.

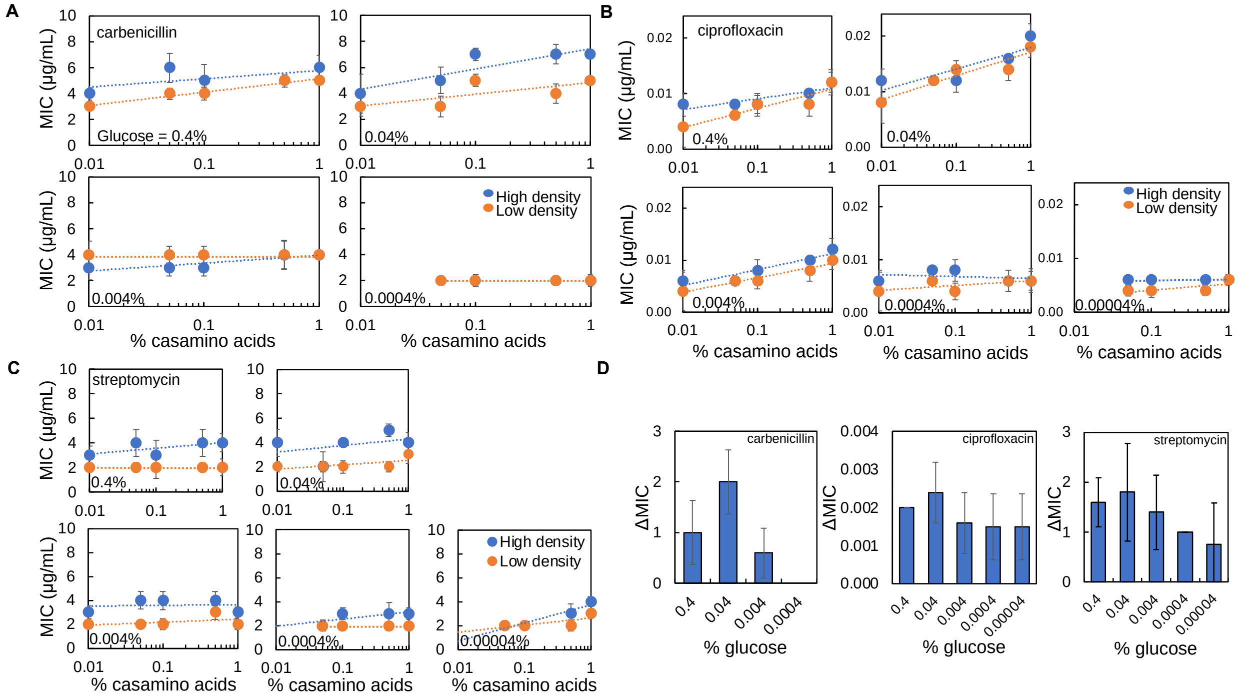


**Fig. S4. Raw data for different antibiotics in Fig. 2C.** MIC of initial high (blue) and low (orange) density populations grown in the presence of:

**A)** carbenicillin. For medium with 0.004% glucose, we took the absolute value of ΔMIC.

**B)** ciprofloxacin, and

**C)** streptomycin. For panels A-C, lines indicate general trend in the data.

**D)** ΔMIC as a function of % glucose for all three antibiotics. For each panel, standard deviation from a minimum of three biological replicates.

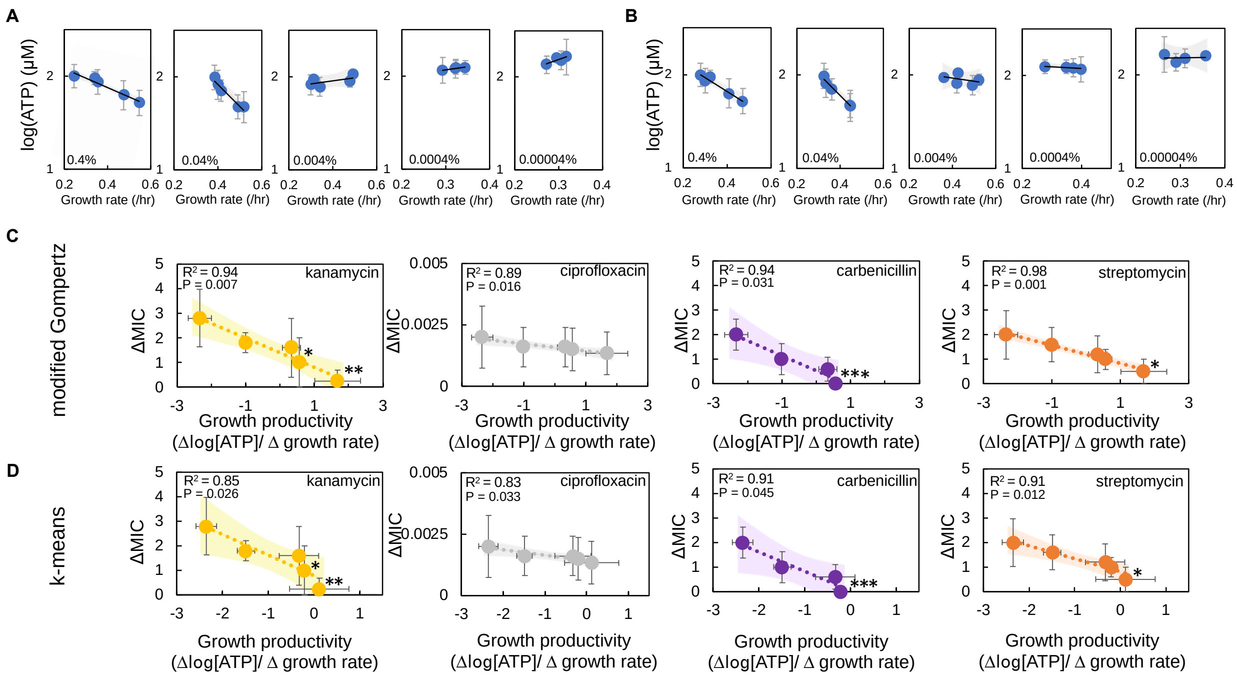

**Fig. S5. Alternative methods to quantify maximum growth rate yield qualitatively similar trends between growth productivity and ΔMIC.**

**A)** The concentration of ATP as a function of maximum growth rate for *E. coli* grown in different percentages of glucose and casamino acids. Maximum growth rate was measured using a modified Gompertz equation. Standard deviation from a minimum of three biological replicates. In both panels A and B, the slope of each linear line represents growth productivity. ATP data replotted from Fig. 1C. Maximum growth rate from three biological replicates. Growth curves in Fig. S1; average residuals for growth curve fitting in Table S3. For all panels, shading indicates 95% confidence interval.

**B)** The concentration of ATP as a function of maximum growth rate for *E. coli* grown in different percentages of glucose and casamino acids. Maximum growth rate was measured using a k-means clustering analysis. Standard deviation from a minimum of three biological replicates.

**C)** ΔMIC of kanamycin, streptomycin, ciprofloxacin and carbenicillin as a function of growth productivity when maximum growth rate was calculated using a modified Gompertz equation. ΔMIC replotted from Fig. 2C. Error bars; x-axis = standard error from linear regression used to determine growth productivity, y-axis = standard deviation. P value and $R^2$ value from simple linear regression shown on panel (additional outputs of this test in Table S9). Deming regression: P = 0.007, 0.0012, 0.03, 0.02 for kanamycin, streptomycin, carbenicillin and ciprofloxacin, respectively. Additional outputs of Deming regression in Table S10. For panels C and D, *P = 0.09, **P=.20, ***P=1, one-tailed t-tests compared to zero.

**D)** ΔMIC of kanamycin, streptomycin, ciprofloxacin and carbenicillin as a function of growth productivity when maximum growth rate was calculated using a k-means clustering analysis. ΔMIC replotted from Fig. 2C. Error bars; x-axis = standard error from linear regression used to determine growth productivity, y-axis = standard deviation. P value and $R^2$ value from simple linear regression shown on panel (additional outputs of this test in Table S9). Deming regression: P = 0.007, 0.0012, 0.03, and 0.02 for kanamycin, streptomycin, carbenicillin and ciprofloxacin, respectively. Additional outputs of Deming regression in Table S10. WLS regression:  P = 0.009, 0.001, 0.010, and 0.052 for kanamycin, streptomycin, carbenicillin and ciprofloxacin, respectively Additional outputs of WLS regression in Table S11.

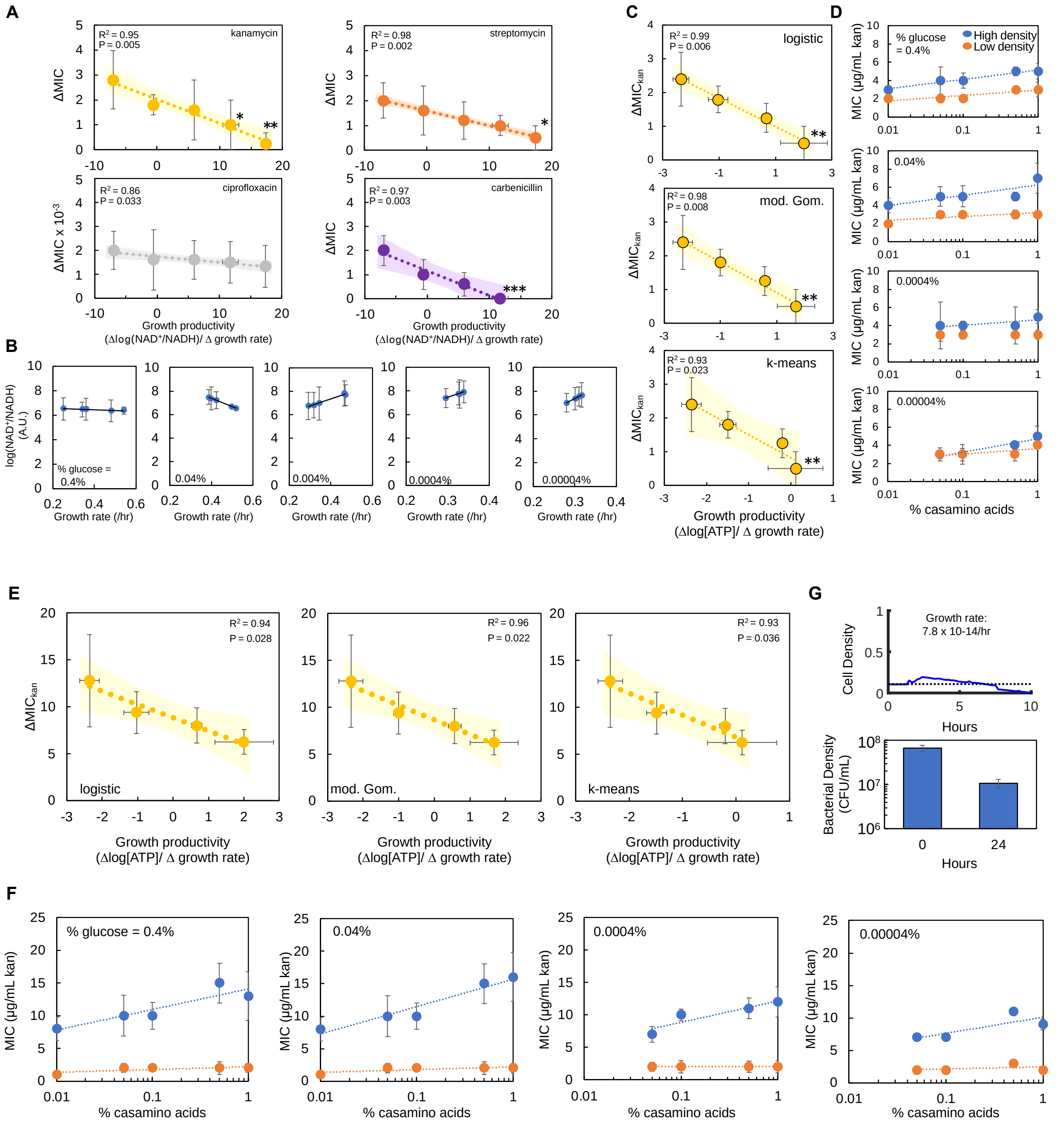

**Figure S6: The relationship between growth productivity and ΔMIC is consistent when measuring NAD$^+$/NADH, experiments initiated from bacteria in mid-log and with a higher initial density of bacteria.**

**A)** ΔMIC of kanamycin, streptomycin, ciprofloxacin and carbenicillin as a function of growth productivity when growth productivity was determined using NAD$^+$/NADH. Maximum

growth rate calculated using a logistic equation. ΔMIC replotted from Fig. 2C. For panels A, C and E: Error bars; x-axis = standard error from linear regression used to determine growth productivity , y-axis = standard deviation. P value and $R^2$ value from simple linear regression shown on panel (summary statistics in Table S12). The relationship between ΔMIC and growth productivity was strong and significant ($P < 0.05$) when using a Deming regression and a WLS regression (Table S12). For panels A and C, *P = 0.09, **P=.20, ***P=1, one-tailed t-tests compared to zero. For panels A, B, C and E, shading indicates 95% confidence interval.

**B)** Luminescence produced by $NAD^+$/NADH as a function of maximum growth rate for *E. coli* grown in different percentages of glucose and casamino acids. Maximum growth rate was measured using a logistic equation. Standard deviation from a minimum of three biological replicates. The slope of each linear line represents growth productivity. $NAD^+$/NADH measurements from a minimum of three biological replicates. Maximum growth rate from three biological replicates. Statistical information for the linear fit can be found in Table S8.

**C)** $\Delta MIC_{kan}$ as a function of growth productivity when experiments were initiated from bacteria in mid-log phase (high initial density = $1.89 \times 10^7$ +/- $1.51 \times 10^6$ CFU/mL; low initial density $1.23 \times 10^5$ CFU/mL ± $4.99 \times 10^4$ CFU/mL). Maximum growth rate calculated using a logistic equation (top), modified Gompertz equation (middle) or k-means clustering analysis (bottom). Standard deviation from a minimum of three biological replicates.

**D)** Raw data for panel C. MIC of initial high (blue) and low (orange) density populations grown in the presence of kanamycin. Standard deviation from a minimum of three biological replicates.

**E)** $\Delta MIC_{kan}$ as a function of growth productivity when a higher initial density of bacteria was used (1.03 x $10^7$+/- 3.70 x $10^6$ CFU/mL). Maximum growth rate calculated using a logistic equation (left), modified Gompertz equation (middle) or k-means clustering analysis (right). $\Delta MIC_{kan}$ calculated using a minimum of 4 percentages of casamino acids per percentage of glucose tested. $\Delta MIC_{kan}$ was calculated from the above higher initial density (500-fold dilution) and the low initial density used through the rest of the manuscript (50,000-fold dilution). Standard deviation from a minimum of three biological replicates. The relationship was also significant when tested using WLS regression or Deming regression (Table S13).

**F)** Raw data for panel E. MIC of initial high (blue) and low (orange) density populations grown in the presence of kanamycin. Standard deviation from a minimum of three biological replicates.

**G)** Growth curve (rate shown on plot, top) and change in CFU (0 hr vs 24 hrs) of bacteria diluted 50-fold from an overnight culture and grown in 0.00004% medium with 0.05% casamino acids. We chose to grow bacteria in this medium as it has the lowest carrying capacity measured in our experiments. Growth curve from the average of three biological replicates. CFU measurements from three biological replicates. Because bacteria did not increase in density over 24 hours from a 50-fold dilution, we could not use this initial density to measure IE.

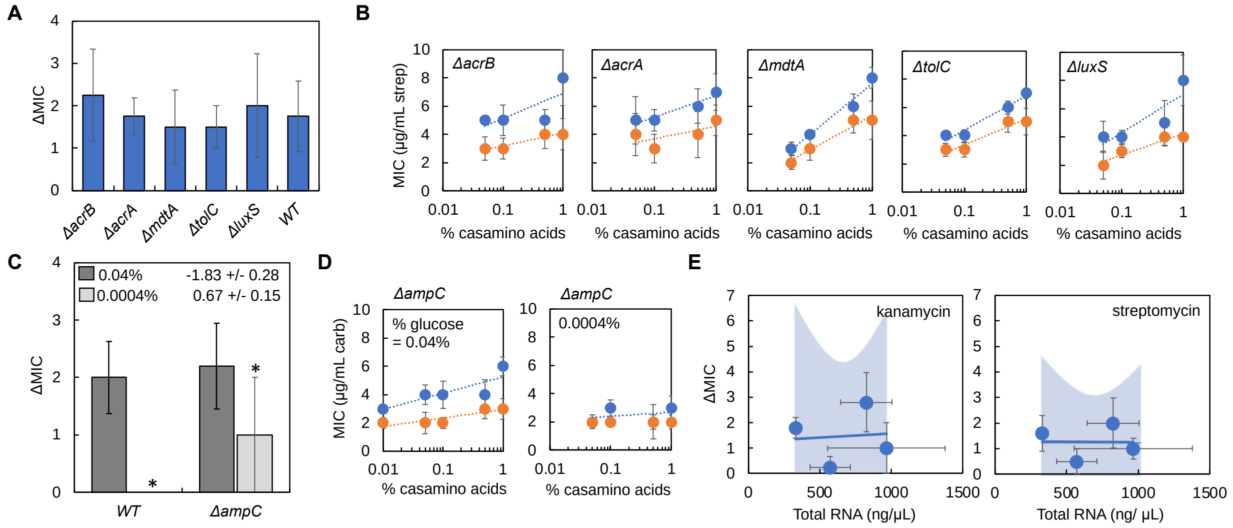


**Figure S7: Alternative hypotheses to explain IE and ΔMIC.**

**A)** ΔMIC of streptomycin using BW25133 knockout strains lacking efflux pumps (*acr*A, *acr*B, *tol*C, *mdt*A) and the ability to perform quorum sensing (*lux*S) as compared to the wildtype (WT) strain. ΔMIC determined in medium with 0.04% glucose containing four concentrations of casamino acids. None of the ΔMIC values were different from the WT strain (P >0.54, two-tailed t-test as compared to WT, ANOVA = 0.88). Streptomycin was used as the knockout strains contain a kanamycin resistance marker.

**B)** Raw data for panel A. MIC of initial high (blue) and low (orange) density populations grown in the presence of streptomycin. Standard deviation from a minimum of three biological replicates.

**C)** ΔMIC of carbenicillin using a BW25133 knockout strain lacking *amp*C. At both percentages of glucose tested (0.04%, lowest growth productivity; 0.0004% highest growth productivity tested with carbenicillin and BW25113), ΔMIC was not significantly different between the WT and *amp*C knockout strain (P > 0.19, two-tailed t-test). ΔMIC also decreased with

increasing growth productivity (values for BW25113 shown on plot next to percent glucose). Finally, ΔMIC was not different from zero for both the WT and *amp*C knockout strains when grown in medium with 0.0004% (*, $P > 0.09$, one-tailed t-test).

**D)** Raw data for panel C. MIC of initial high (blue) and low (orange) density populations grown in the presence of carbenicillin. Standard deviation from a minimum of three biological replicates.

**E)** Total RNA as a function of ΔMIC for kanamycin (right) and streptomycin (left). $R^2$ and P value on plot are from a linear regression. Shading = 95% confidence interval. ΔMIC replotted from Fig. 2C. Standard deviation on x-axis from 3 biological replicates, each consisting of two technical replicates.

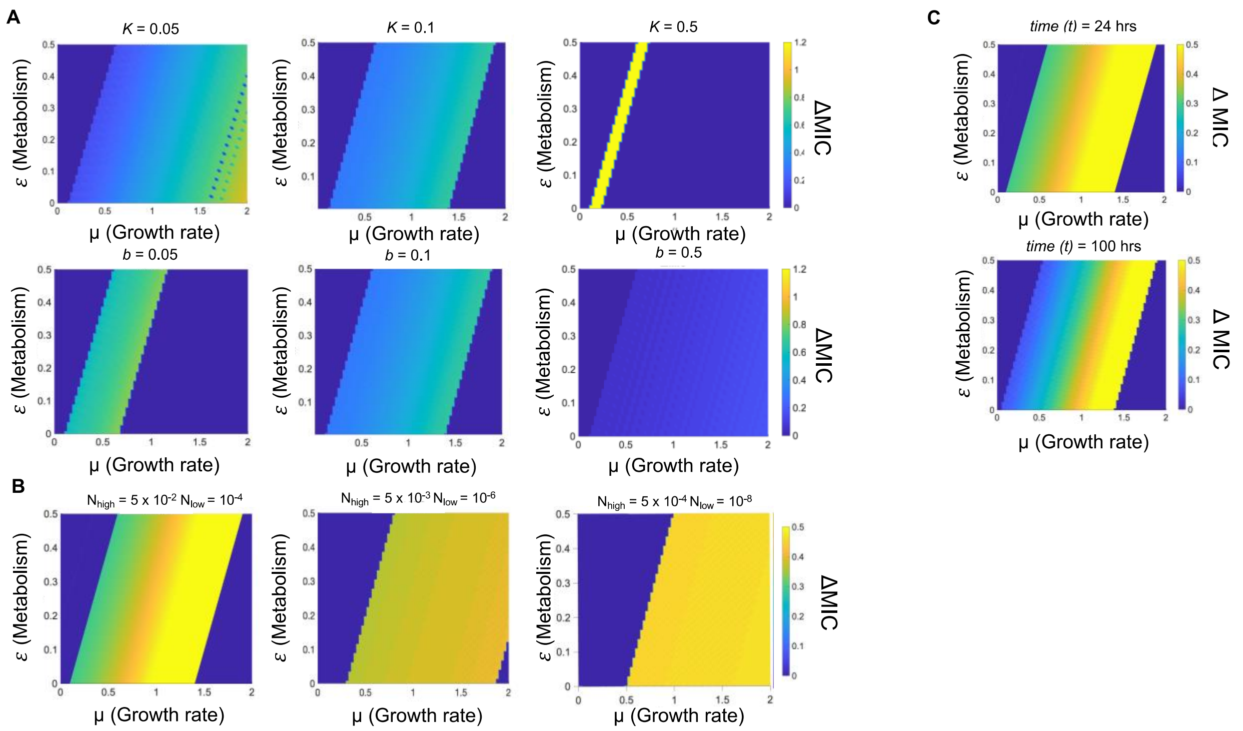


**Fig. S8. Sensitivity analysis for our model (Eq. 1, *Methods,* main text).**

- **A)** Sensitivity analysis for model parameters $\mu$, $\varepsilon$, $K$ and $b$, and their effect of changing ΔMIC. $t$ = 24 hrs.
- **B)** The general trends of our modeling predictions hold true when the initial density of the populations is decreased. Initial density indicated on the panel. $N_{high}$ and $N_{low}$ are high initial and low initial density, respectively.
- **C)** Our model continues to predict the qualitative trends in our simulations over a longer simulation time. Top: $t$ = 24 hrs, bottom: $t$ = 100 hrs.

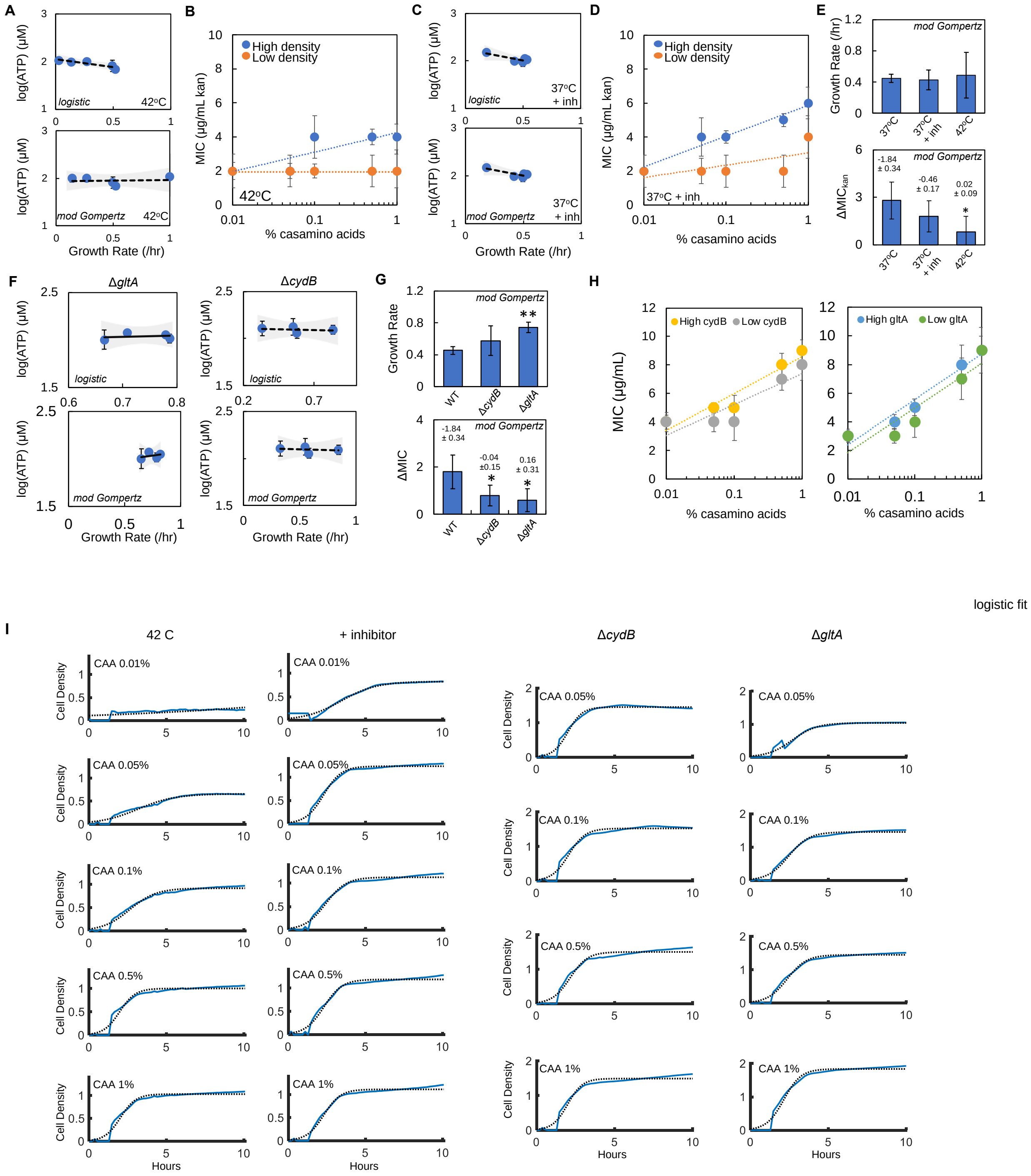

A
B
C
D
E
F
G
H
I
log(ATP) (µM)
logistic
42ºC
mod Gompertz
Growth Rate (/hr)
MIC (µg/mL kan)
High density
Low density
% casamino acids
37ºC + inh
Growth Rate (/hr)
ΔMICkan
-1.84 ± 0.34
-0.46 ± 0.17
0.02 ± 0.09
37ºC
42ºC
ΔgltA
ΔcydB
Growth Rate
ΔMIC
-1.84 ± 0.34
-0.04 ±0.15
0.16 ± 0.31
WT
MIC (µg/mL)
High cydB
Low cydB
High gltA
Low gltA
logistic fit
42 C
+ inhibitor
Cell Density
CAA 0.01%
CAA 0.05%
CAA 0.1%
CAA 0.5%
CAA 1%
Hours

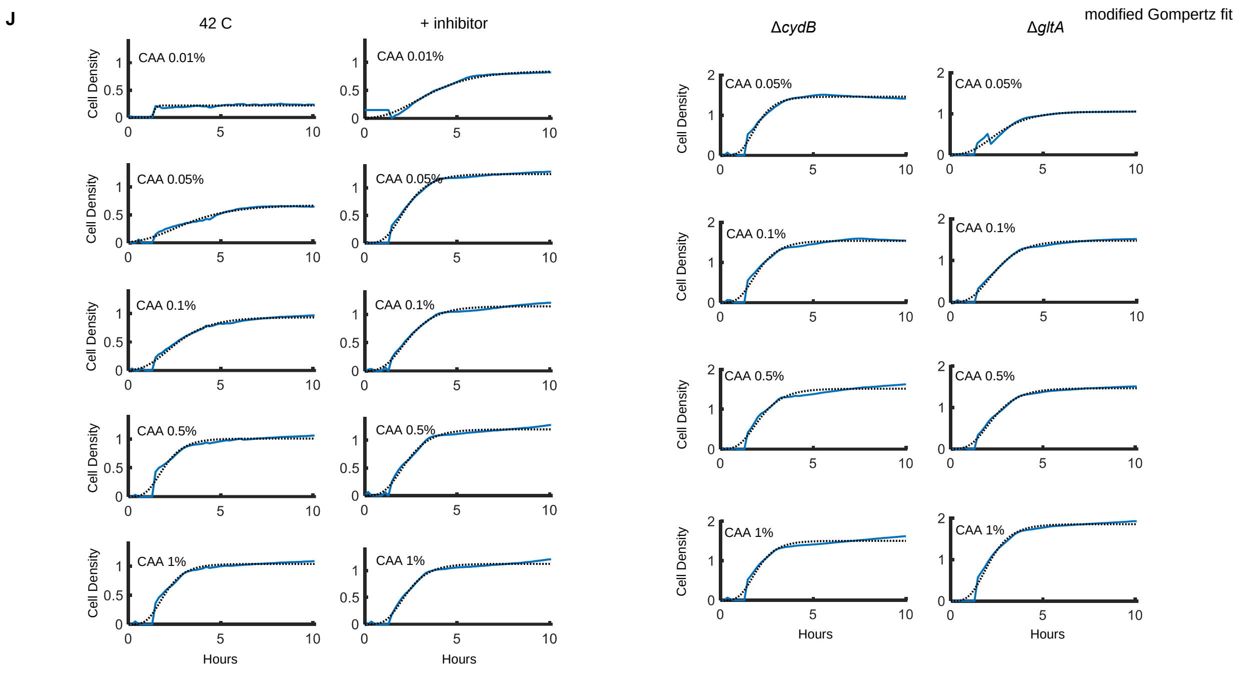


**Fig. S9. Raw data for Fig. 4 and Fig. 5.**

**A)** [ATP] as a function of maximum growth rate for *E. coli* grown at 42°C. The slope of the linear line was used to determine growth productivity. For panels A, C and F, ATP measurements consist of three biological replicates, each with three technical replicates. Top: Maximum growth rate measured using a logistic equation (Eq. S1). Bottom: Maximum growth rate measured using a modified Gompertz equation (Eq. S2). In both cases, maximum growth rate measured from the average of three biological replicates. Shading in panels A, C and F represents 95% confidence interval.

**B)** Raw MIC data for kanamycin for *E. coli* grown in M9 medium (glucose = 0.04%) at 42°C. Standard deviation from a minimum of five biological replicates. For panels B, D, and H, lines indicate general trend in the data.

**C)** [ATP] as a function of maximum growth rate for *E. coli* grown at 37°C and in 10 ng/mL of PMSF (serine protease inhibitor, + inh). The slope of the linear line was used to determine growth productivity. Top: Maximum growth rate measured using a logistic equation (Eq.

S1). Bottom: Maximum growth rate measured using a modified Gompertz equation (Eq. S2). In both cases, growth rate measured from average of three biological replicates.

**D)** Raw MIC data for kanamycin for *E. coli* grown in M9 medium (glucose = 0.04%) at 37°C and with 10 ng/mL PMSF. Standard deviation from a minimum of five biological replicates.

**E)** Average maximum growth rate (top) and ΔMIC (bottom) of *E. coli* grown at 37°C, at 42°C or at 37°C with 10 ng/mL of serine protease inhibitor (+ inh) in medium with 0.04% glucose. Maximum growth rate calculated using a modified Gompertz equation. $\Delta MIC_{kan}$ data replotted from Fig. 4. * indicates significant decrease in average MIC (P = 0.017, one-tailed t-test). Values above bars on bottom panel represent growth productivity with standard error (from linear regression used to determine growth productivity).

**F)** Growth productivity for *ΔgltA* (right) and *ΔcydB* (left) *E. coli* knockout strains. Top: Maximum growth rate measured using a logistic equation. Bottom: Maximum growth rate measured using a modified Gompertz equation. In both cases, growth rate measured from average of three biological replicates.

**G)** Average maximum growth rate (top) and ΔMIC (bottom) of streptomycin for *ΔgltA* (right) and *ΔcydB* (left) *E. coli* knockout strains grown in 0.04% glucose. Maximum growth rate calculated using a modified Gompertz equation. ΔMIC data replotted from Fig. 5. ** indicates significant increase in maximum growth rate (P = 0.0005), * indicates significant decrease in average ΔMIC (P = 0.001 for *ΔcydB*, P = 0.008 for *ΔgltA*, one-tailed t-tests). Values above bars on bottom panel represent growth productivity with standard error (from linear regression used to determine growth productivity).

**H)** Raw MIC data for streptomycin for the *ΔgltA* (right) and *ΔcydB* (left) *E. coli* knockout strains grown in M9 medium (glucose = 0.04%). We could not use kanamycin as strains from the

Keio collection are kanamycin resistant (*49*). Standard deviation from a minimum of five biological replicates.

**I)** Growth curves of *E. coli* grown in 42ºC, at 37ºC and in 10 ng/mL of PMSF (serine protease inhibitor, + inh), and with *ΔgltA* or *ΔcydB* knocked out. Maximum growth rate estimated by fitting the data using a logistic equation (Eq. S1). Percentage of casamino acids in the growth medium as indicated on the figure. For all panels, maximum growth rate calculated from the average of three biological replicates, dotted black line indicates fitting and average residuals for fits are found in Table 3. To determine cell density, $OD_{600}$ values were log-transformed and normalized to the initial minimum density. For sub panels in I-J, y = cell density, x = time in hours. CAA = casamino acids.

**J)** Growth curves fit using a modified Gompertz equation (Eq. S2).

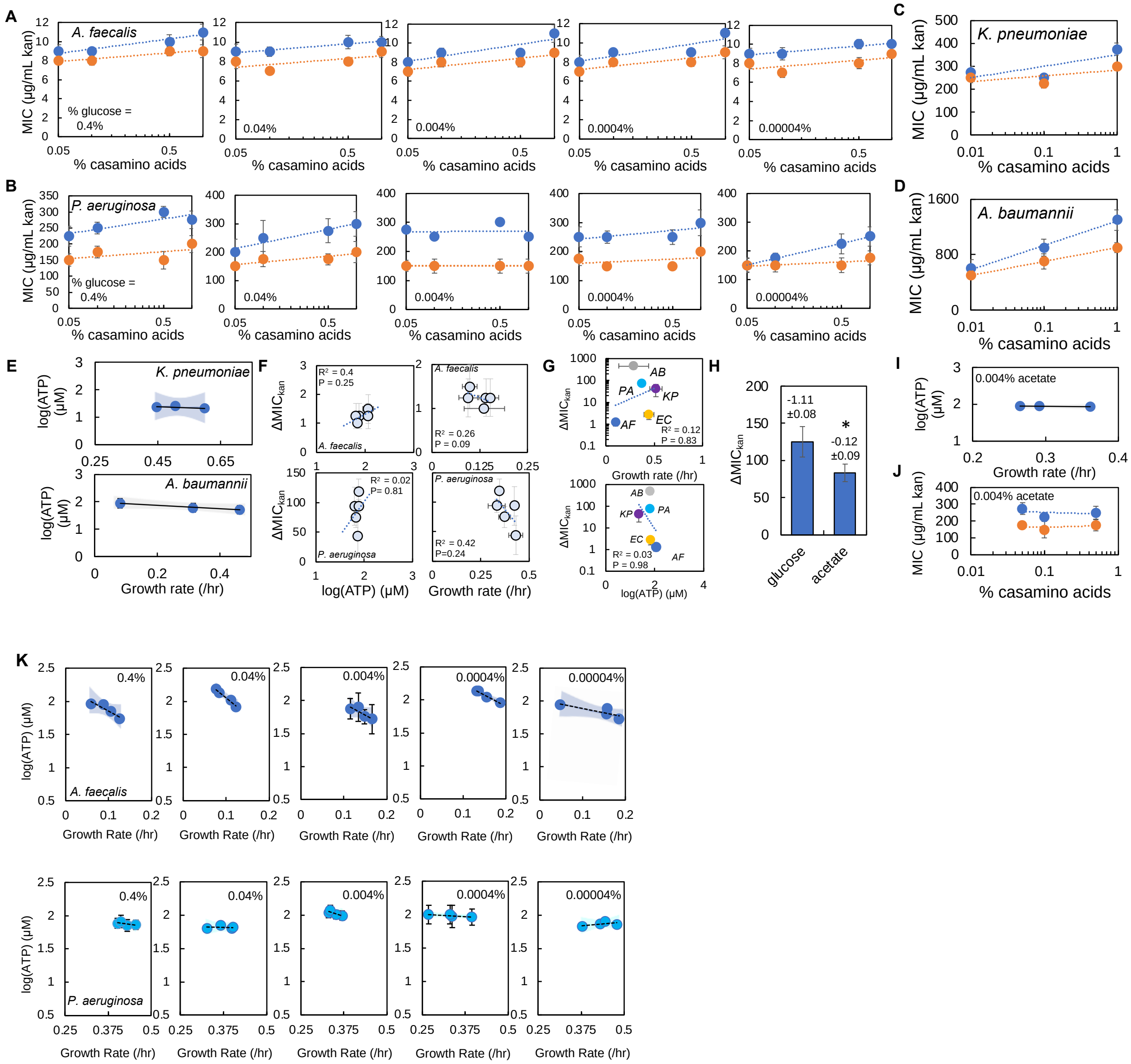

A
B
C
D
E
F
G
H
I
J
K
A. faecalis
P. aeruginosa
K. pneumoniae
A. baumannii
MIC (µg/mL kan)
% casamino acids
% glucose = 0.4%
0.04%
0.004%
0.0004%
0.00004%
log(ATP) (µM)
Growth rate (/hr)
ΔMIC$_{kan}$
$R^2$ = 0.4 P = 0.25
$R^2$ = 0.26 P = 0.09
$R^2$ = 0.02 P= 0.81
$R^2$ = 0.42 P=0.24
$R^2$ = 0.12 P = 0.83
$R^2$ = 0.03 P = 0.98
AB
PA
KP
EC
AF
-1.11 ±0.08
-0.12 ±0.09
*
glucose
acetate
0.004% acetate
Growth Rate (/hr)

L

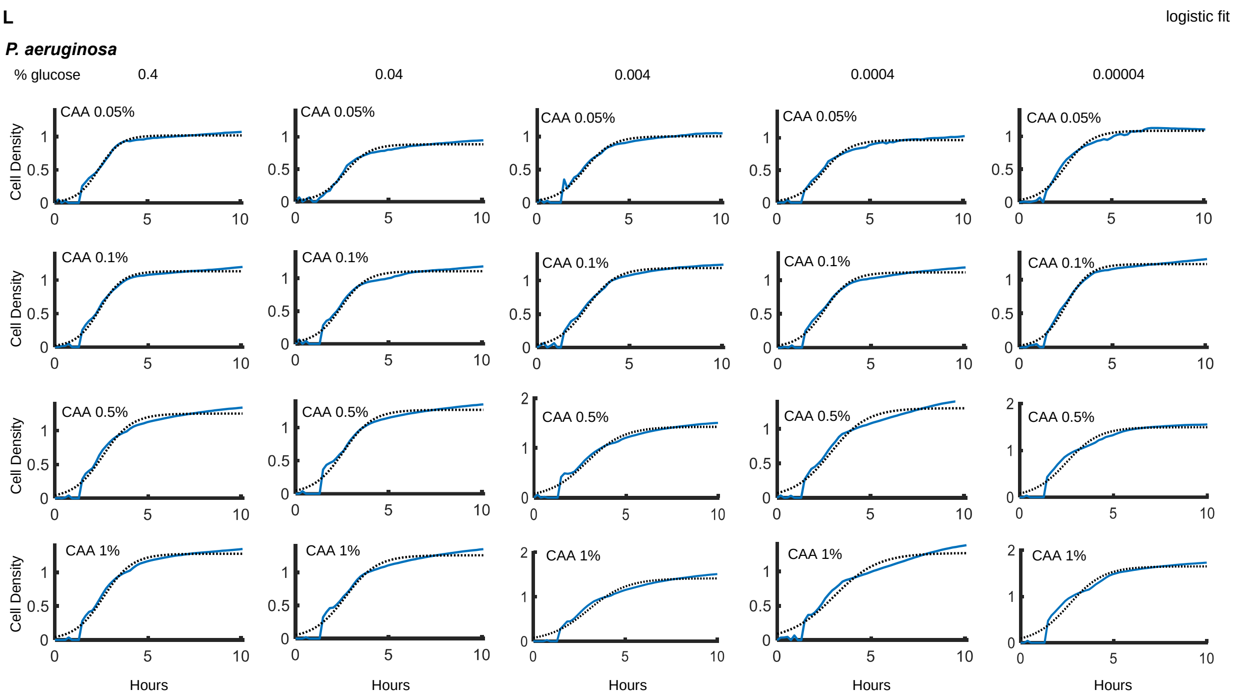
logistic fit
P. aeruginosa
% glucose
0.4
0.04
0.004
0.0004
0.00004
CAA 0.05%
CAA 0.1%
CAA 0.5%
CAA 1%
Cell Density
Hours

M

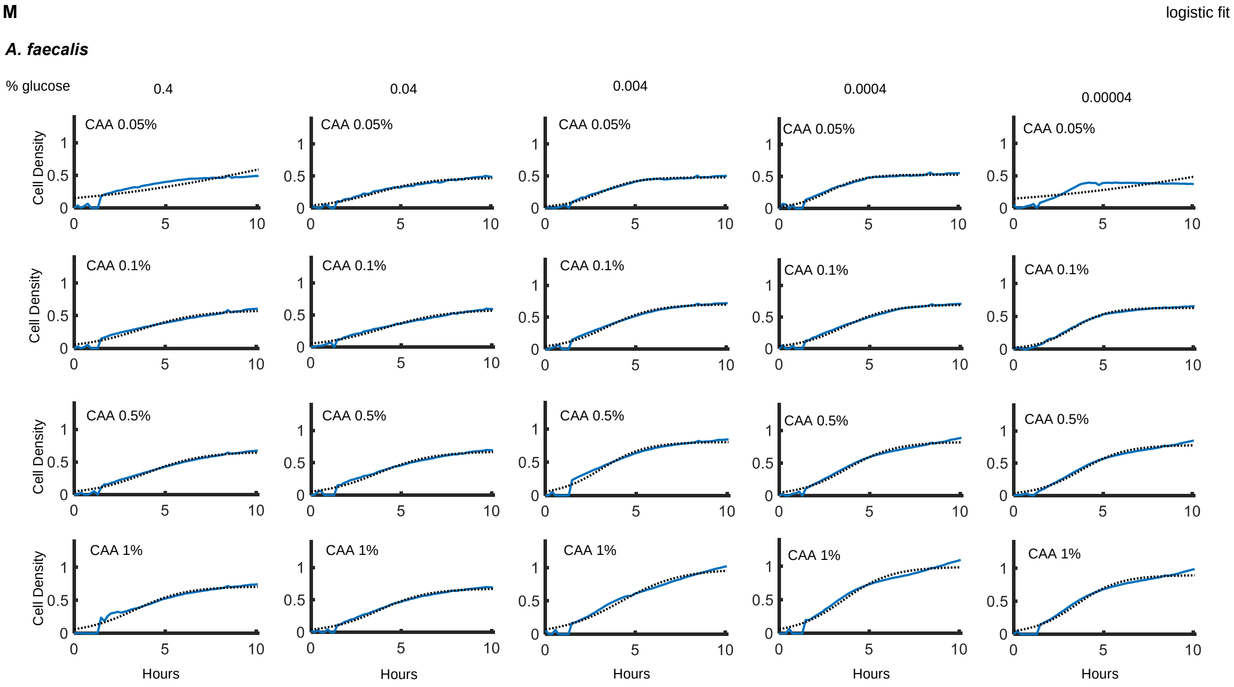
logistic fit
A. faecalis
% glucose
0.4
0.04
0.004
0.0004
0.00004
CAA 0.05%
CAA 0.1%
CAA 0.5%
CAA 1%
Cell Density
Hours

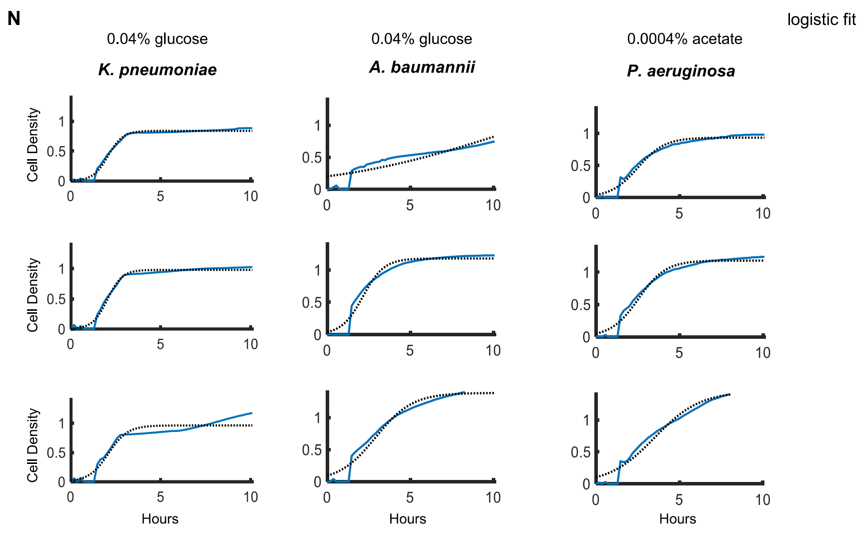


**Fig. S10. Raw data for different bacterial species presented in Fig. 6.**

**A)** MIC of kanamycin for initial high and low density of *A. faecalis*. For panels A-D, standard deviation from a minimum of three biological replicates. For panels A, B, C, D, and J, lines indicate general trend in the data.

**B)** MIC of kanamycin for of initial high and low density of *P. aeruginosa*.

**C)** MIC of kanamycin for of initial high and low density of *K. pneumoniae*. For panels C and D, medium contained 0.04% glucose.

**D)** MIC of kanamycin for of initial high and low density of *A. baumannii*.

**E)** Growth productivity of *K. pneumoniae* (top) and *A. baumannii* (bottom) grown in M9 medium. Glucose = 0.04%. Casamino acid concentrations: 0.05%, 0.1%, 0.5% and 1% were used to measure ATP and maximum growth rate. ATP measurements consist of three biological replicates, each with three technical replicates. Maximum growth rate measured from the average of three biological replicates using a logistic equation. Growth curves shown in panel N. Shading indicates 95% confidence interval.

**F)** $\Delta MIC_{kan}$ of *A. faecalis* (top) and *P. aeruginosa* (bottom) as a function of average ATP concentration (left) and maximum growth rate (right). ATP concentration and maximum

growth rate averaged across five concentrations of glucose (and four percentages of casamino acids). We did not find a linear relationship between these variables in these species (P values indicated on plot, simple linear regressions).

**G)** $\Delta MIC_{kan}$ for each bacterial species measured in this study plotted as a function of maximum growth rate (top) or ATP (bottom). Glucose =0.04%. We did not find a linear relationship between these variables (P values indicated on plot, simple linear regression).

**H)** $\Delta MIC_{kan}$ of *P. aeruginosa* grown in medium containing 0.004% glucose or acetate. Growth productivity indicated above each bar with standard error shown (from linear regression used to determine growth productivity). We observed a significant reduction in $\Delta MIC_{kan}$ when *P. aeruginosa* was grown in acetate as compared to glucose (* P = 0.041, one-tailed t-test). Error bars = standard deviation.

**I)** [ATP] as a function of maximum growth rate for *P. aeruginosa* grown in M9 medium containing 0.0004% acetate. Maximum growth rate determined using a logistic equation (see *Methods*). Shading indicates 95% confidence interval.

**J)** MIC of kanamycin for initial high and low density of *P. aeruginosa* grown in M9 medium with 0.0004% acetate. Standard deviation from a minimum of five biological replicates.

**K)** The concentration of ATP as a function of maximum growth rate (/hr) for *A. faecalis* (top) and *P. aeruginosa* (bottom) grown in different percentages of glucose and casamino acids. The slope of each linear line was used to estimate growth productivity. ATP measurements from a minimum of three biological replicates, each consisting of a minimum of three technical replicates. Maximum growth rate from the average of three biological replicates. Shading indicates 95% confidence interval. Error bars = standard deviation.

**L)** Growth curves and fitting of *P. aeruginosa* grown in M9 medium. A logistic equation (Eq. S1) was used to fit this data. For panels L-N, dotted black line indicates fitting. Solid line is

experimental data. To determine cell density, $OD_{600}$ values were log-transformed and normalized to the initial minimum density. Percentage of carbon source indicated above each panel. Percentage of casamino acids in the growth medium as indicated with different colored lines on the figure. Average residuals for fit are found in Table 3. CAA = casamino acids. For all sub panels in L-N, y = cell density, x = time in hours.

**M)** Growth curves and fitting of *A. faecalis* grown in M9 medium.

**N)** Growth curves and fitting of *K. pneumoniae* (0.04% glucose), *A. baumannii* (0.04% glucose) *and P. aeruginosa* (0.0004% acetate) grown in M9 medium.

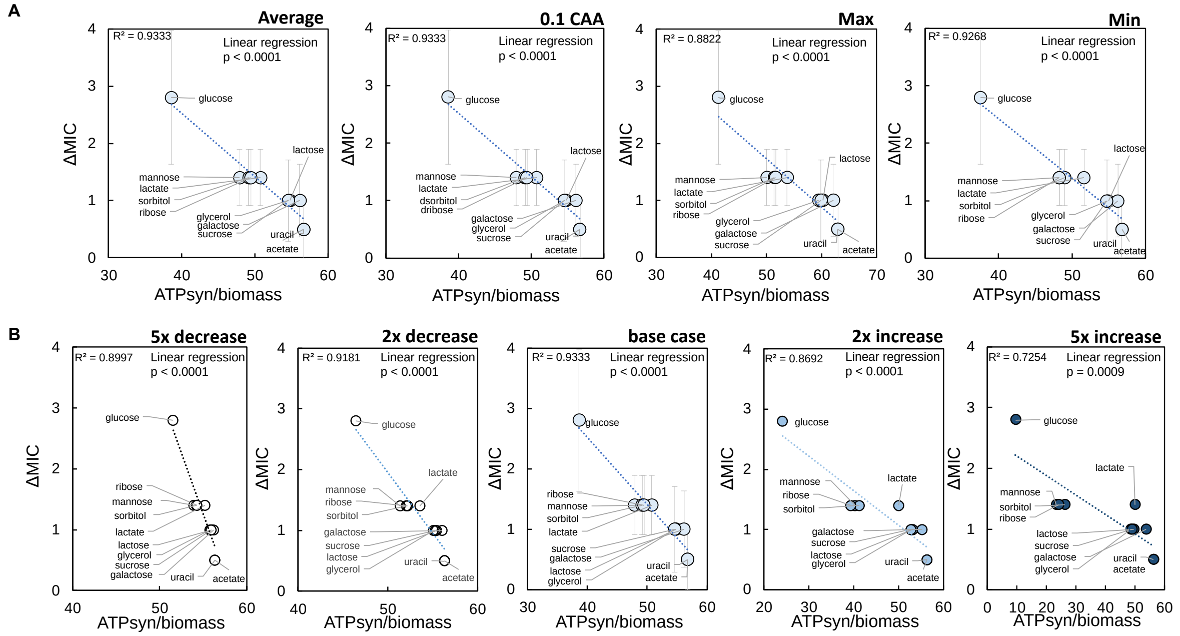


**Fig. S11. Sensitivity analysis of our flux balance analysis (FBA).**

**A)** Sensitivity analysis for lower bound exchange flux values for amino acids. We used previously reported exchange values reported in the 0.1% casamino acids condition (*73*) (left center, panel). We also calculated the standard deviation of the averaged flux values (averaged from 0.05%, 0.1% and 0.2% (*73*)). We then added the standard deviation to the calculated average to generate maximum and minimum lower bound exchange values (center, right and right panels). Average (0.2%, 0.1% and 0.05% casamino acids, which are used in the main text) flux values are shown on the left for comparison. For panels A and B, P values from linear regression indicated on each plot. $R^2$ indicated on plots. ATPsyn = ATP synthase activity.

**B)** Sensitivity analysis for lower bound exchange flux values of carbon sources. We independently varied the lower bound exchange value for each carbon source tested in our FBA as indicated above each panel. Despite these variations, the qualitative predictions of our FBA remain consistent.

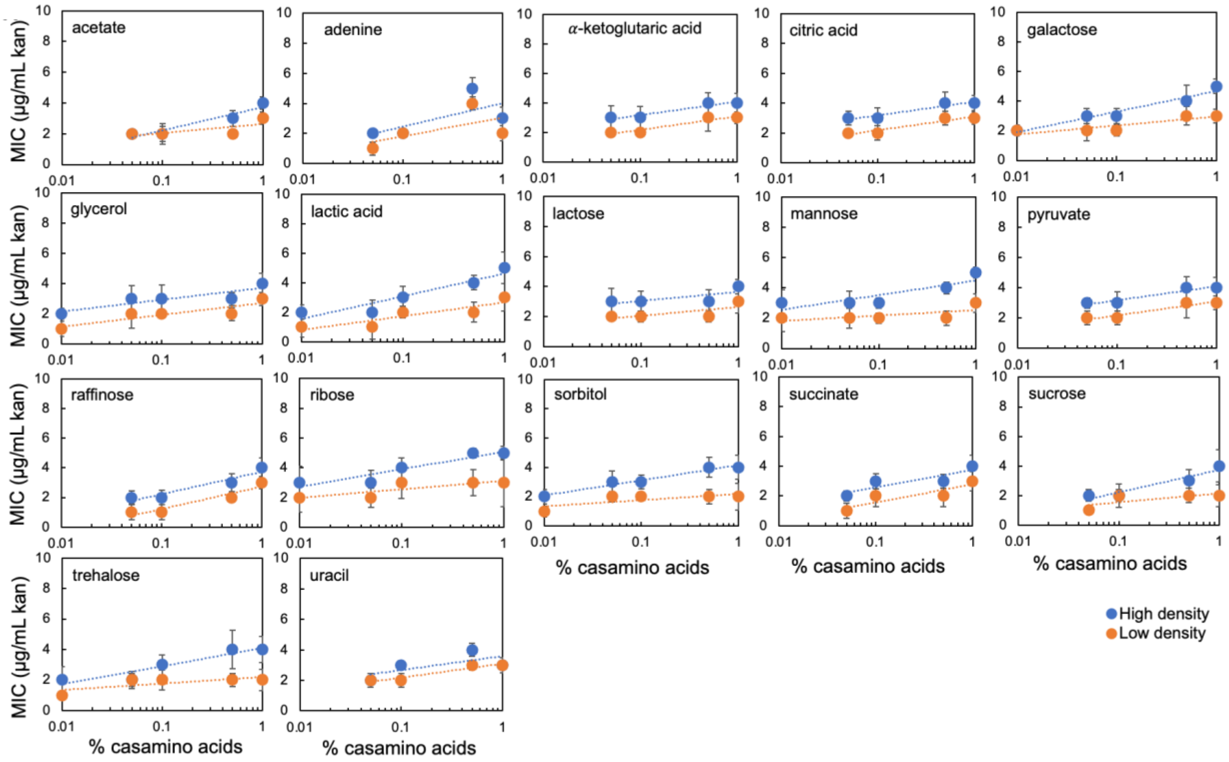


**Fig. S12. Raw MIC data for Fig. 7.** MIC of initial high- and low- density populations grown in the presence of kanamycin. The carbon source used in the growth medium is shown in each panel. Standard deviation from > three biological replicates. Lines indicate general trend in the data.

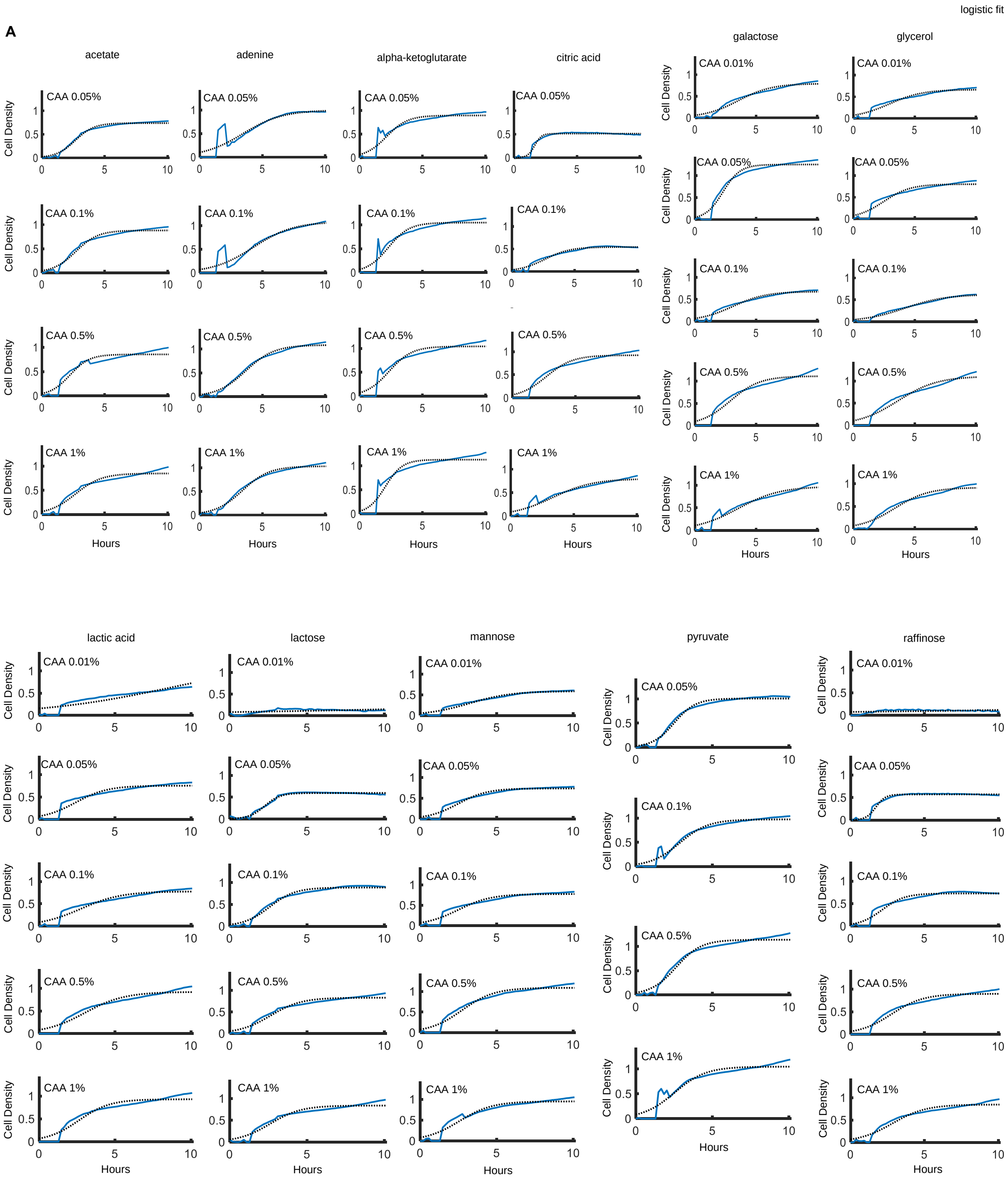
logistic fit
A
acetate
adenine
alpha-ketoglutarate
citric acid
galactose
glycerol
lactic acid
lactose
mannose
pyruvate
raffinose
CAA 0.01%
CAA 0.05%
CAA 0.1%
CAA 0.5%
CAA 1%
Cell Density
Hours

**A (continued)**

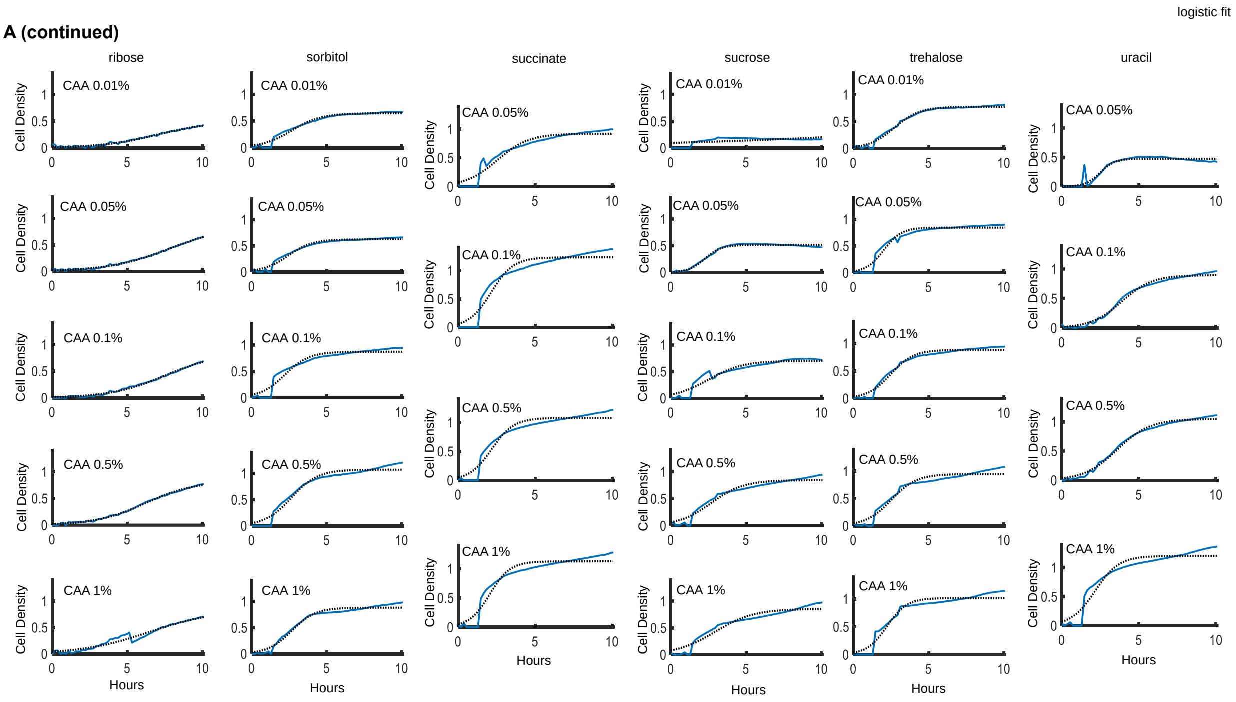


**B**

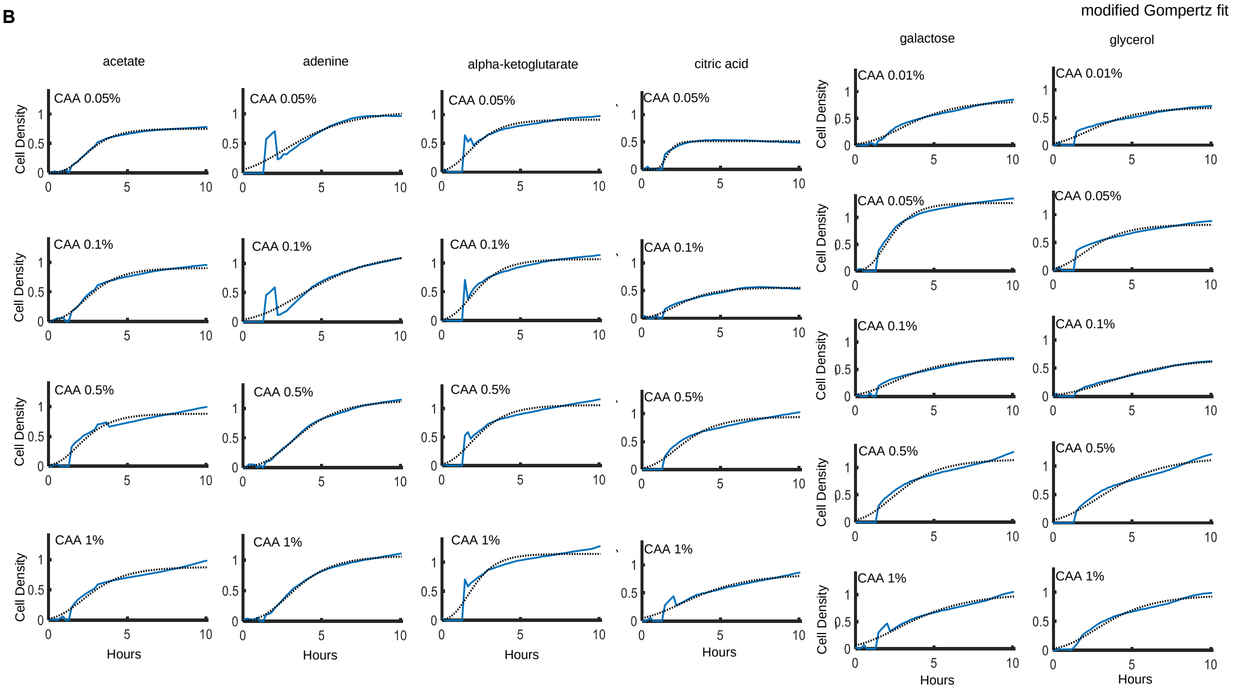

**B (continued)**

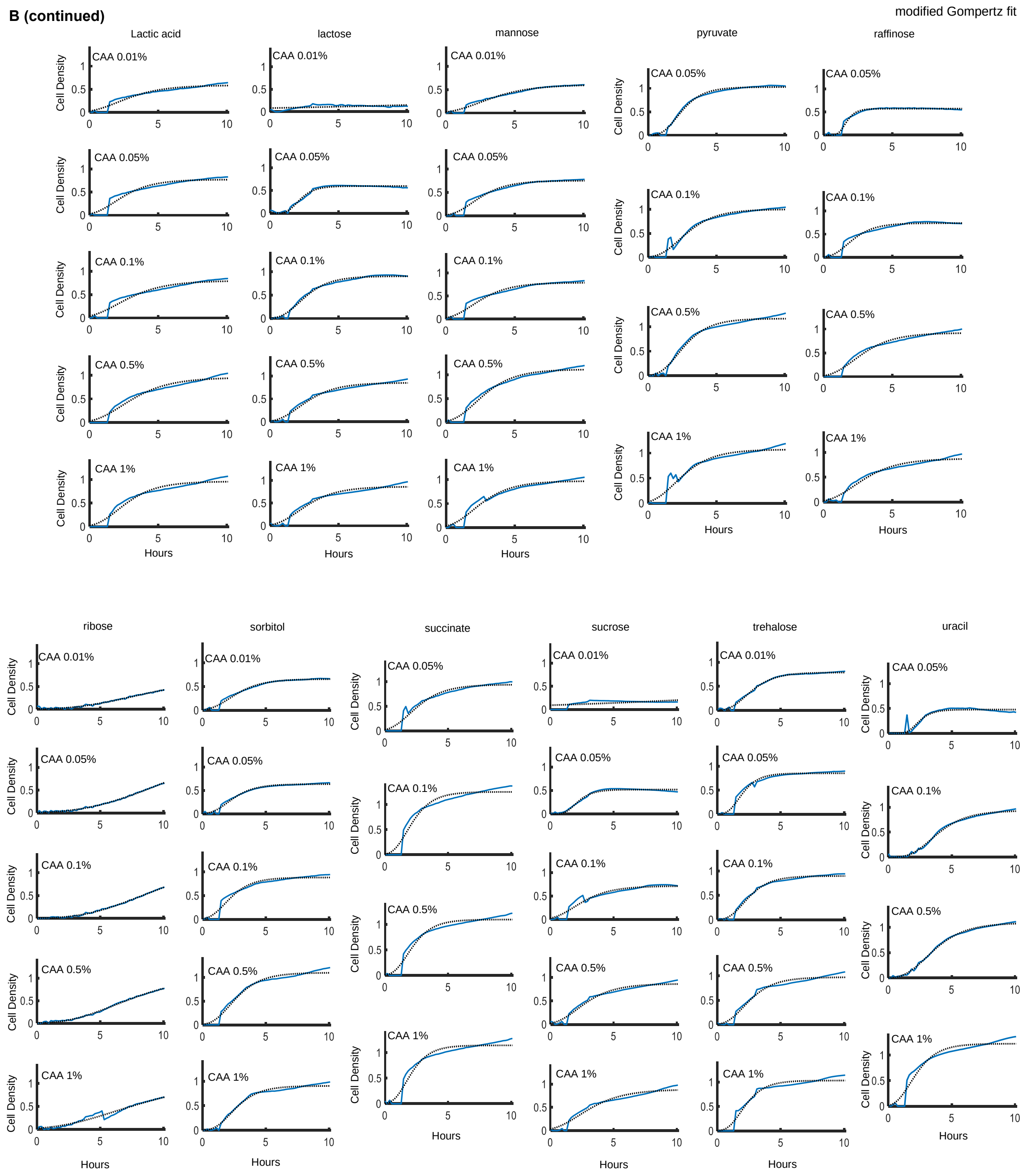
modified Gompertz fit
Lactic acid
lactose
mannose
pyruvate
raffinose
ribose
sorbitol
succinate
sucrose
trehalose
uracil
CAA 0.01%
CAA 0.05%
CAA 0.1%
CAA 0.5%
CAA 1%
Cell Density
Hours

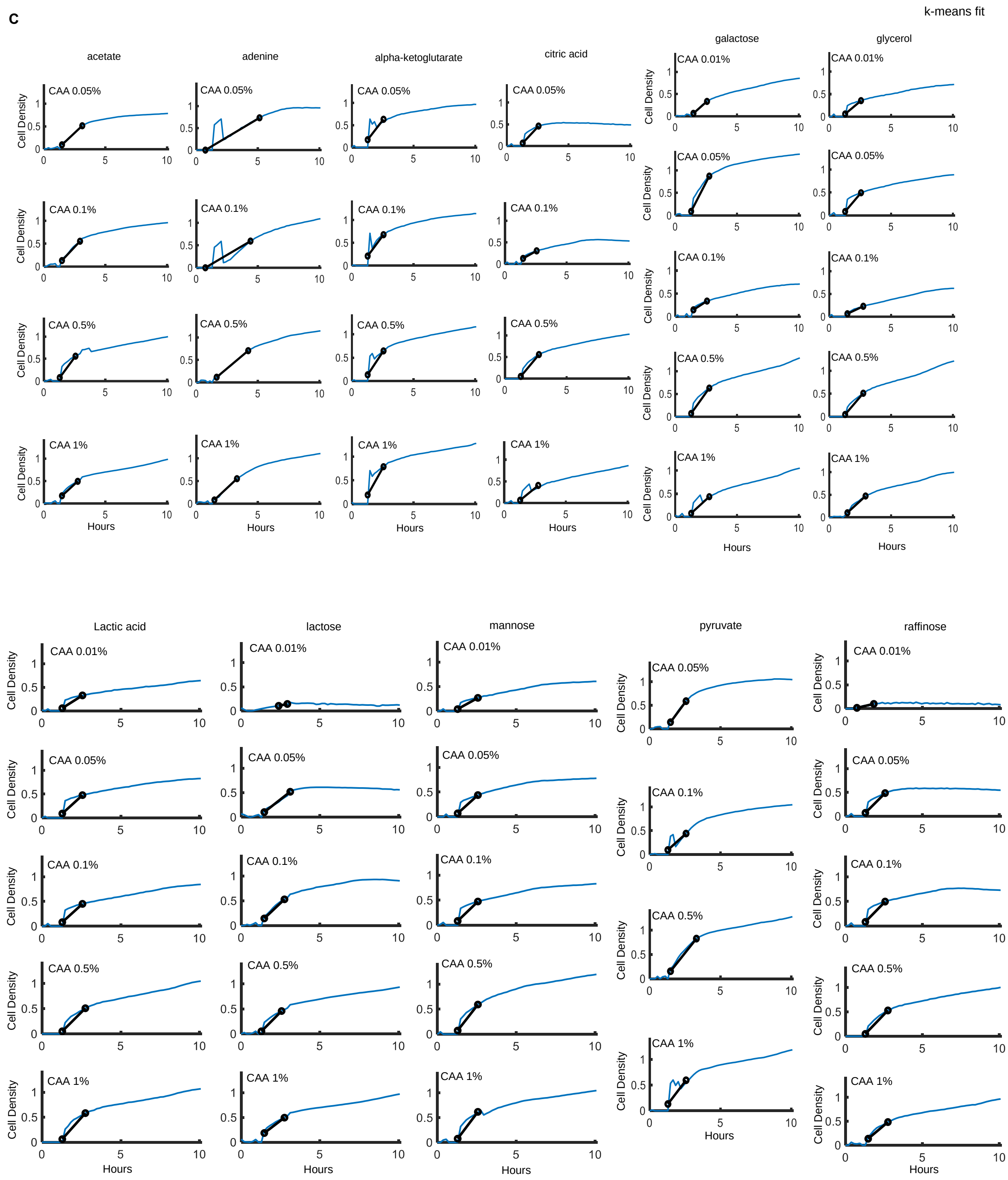

C
k-means fit
acetate
adenine
alpha-ketoglutarate
citric acid
galactose
glycerol
Lactic acid
lactose
mannose
pyruvate
raffinose
CAA 0.01%
CAA 0.05%
CAA 0.1%
CAA 0.5%
CAA 1%
Cell Density
Hours

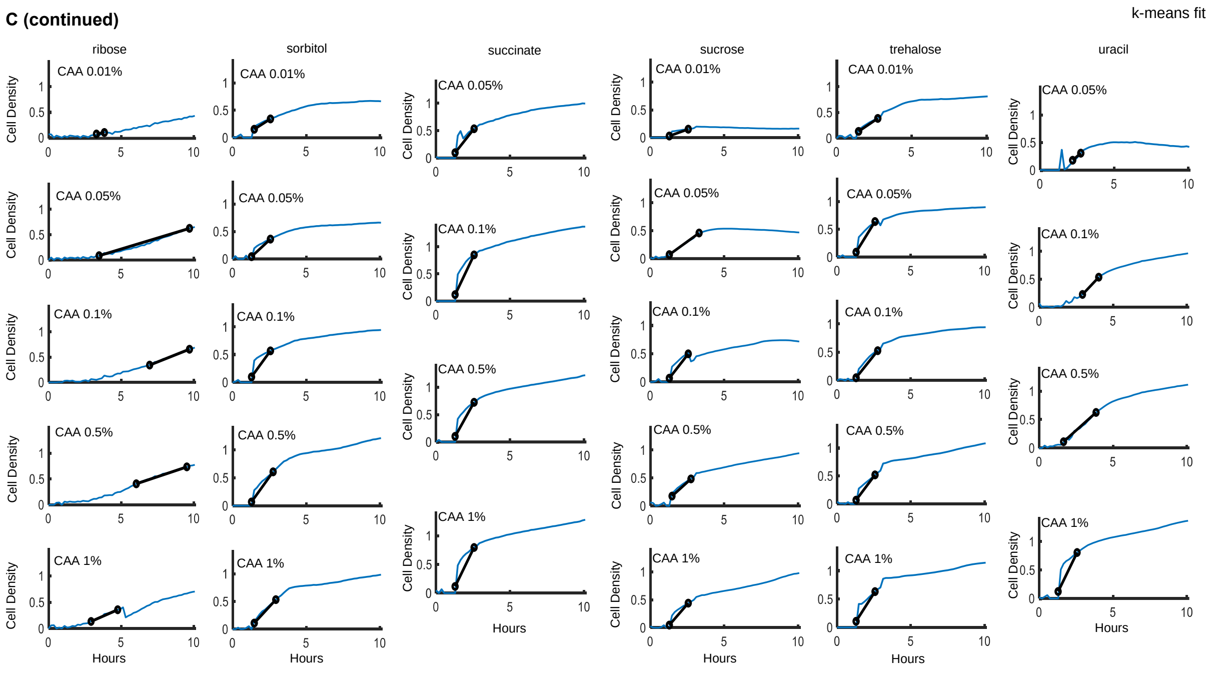


**Fig. S13. Growth curves and fitting for *E. coli* grown in different carbon sources.**

- **A)** Growth curves of *E. coli* grown in M9 medium with different carbon sources (0.04%). In this panel, maximum growth rate was determined using a logistic equation (Eq. S1). Dotted black line indicates fitting. Solid line indicates experimental data. For all panels, to determine cell density, $OD_{600}$ values were log-transformed and normalized to the initial minimum density. Growth curves plotted from the average of three biological replicates. Average residuals for fits in panels A-C are found in Table S3. CAA = casamino acids. For all sub panels in this figure, y = cell density, x = time in hours.
- **B)** Growth curves fit using a modified Gompertz equation (Eq. S2).
- **C)** Growth curves fit using a k-means clustering analysis. Solid black line indicates longest region of fastest growth. Black circles indicate beginning and end point.

A

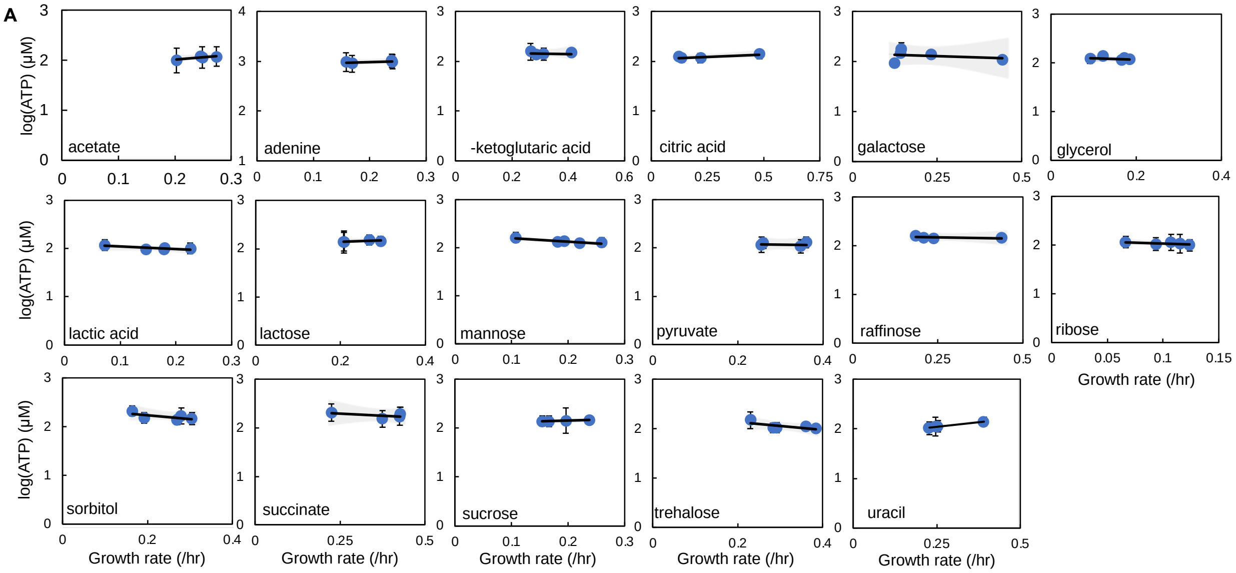
log(ATP) (μM)
Growth rate (/hr)
acetate
adenine
-ketoglutaric acid
citric acid
galactose
glycerol
lactic acid
lactose
mannose
pyruvate
raffinose
ribose
sorbitol
succinate
sucrose
trehalose
uracil

B

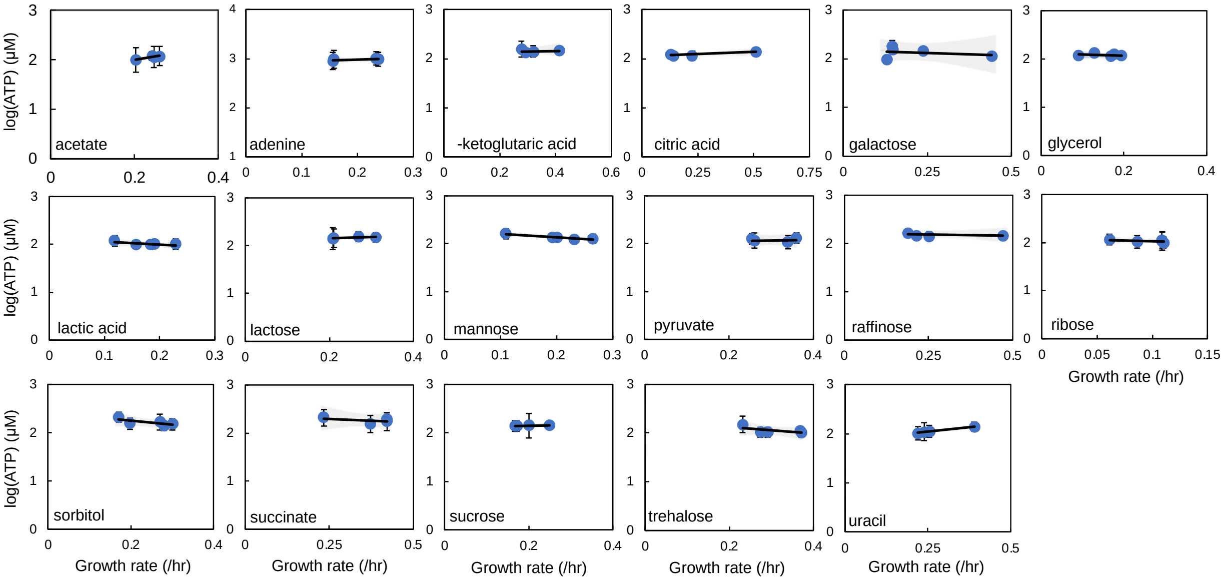
log(ATP) (μM)
Growth rate (/hr)
acetate
adenine
-ketoglutaric acid
citric acid
galactose
glycerol
lactic acid
lactose
mannose
pyruvate
raffinose
ribose
sorbitol
succinate
sucrose
trehalose
uracil

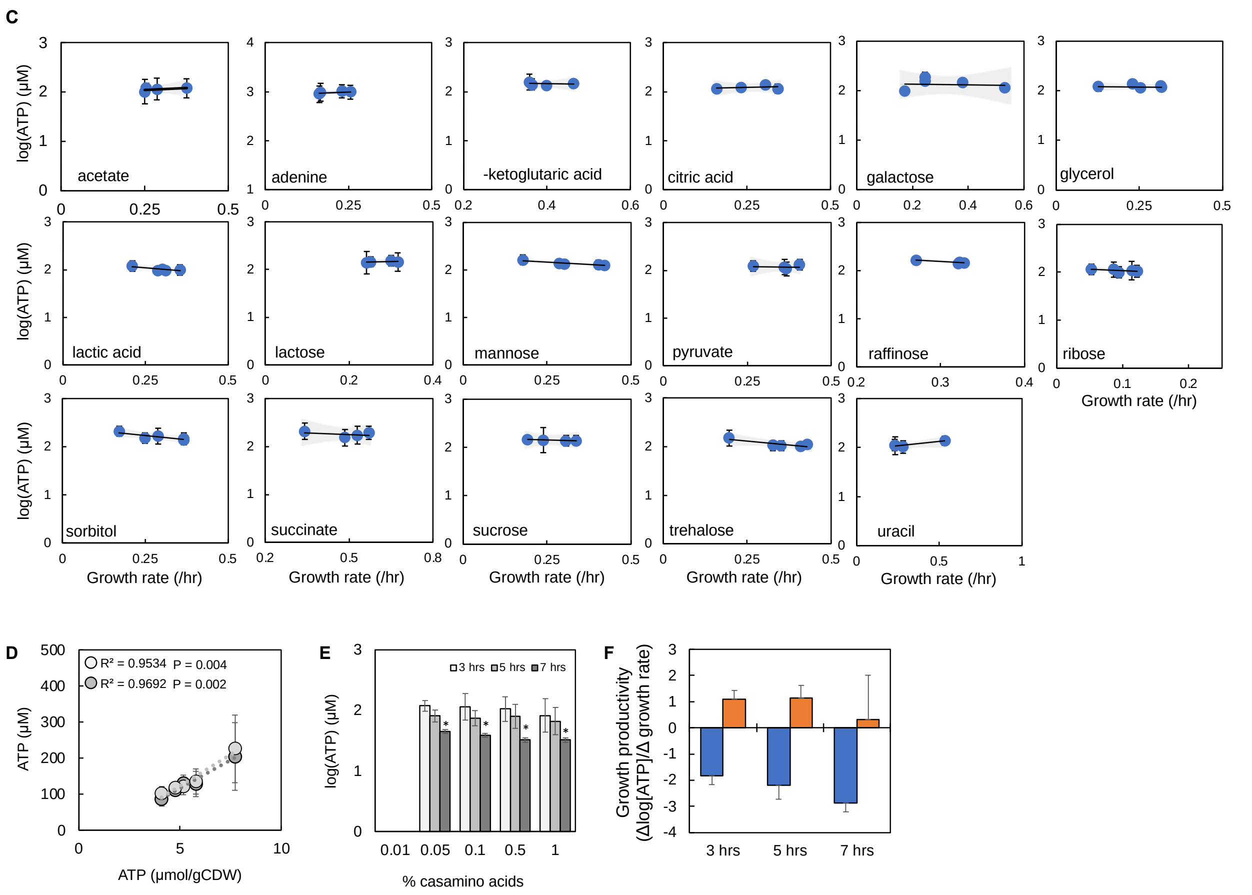


**Fig. S14. Growth productivity of *E. coli* grown in different carbon sources (raw data for Fig. 7 and 8).**

**A)** [ATP] as a function of maximum growth rate as determined using a logistic equation (Eq. S1). For panels A-C, the carbon source used in each growth medium is shown in each panel (0.04%). Standard deviation from > three biological replicates, each consisting of > two technical replicates. Growth curves and fit shown in Fig. S13. For panels A-C, shaded region indicates 95% confidence interval.

**B)** [ATP] as a function of maximum growth rate as determined using a modified Gompertz equation (Eq. S2).

**C)** [ATP] as a function of maximum growth rate as determined using a k-means clustering analysis.

**D)** The relationship between the concentration of ATP found in this study (y-axis) and in (*78*) using *E. coli* strain BW25113 grown in M9 medium. We found a strong and significant linear correlation between the concentrations of ATP (P values indicated on plot, from simple linear regression).

**E)** Measuring [ATP] at different time points during mid-log does not change the concentration of ATP produced when *E. coli* is grown in medium containing acetate. We measured the concentration of ATP in *E. coli* grown in medium with 0.04% acetate after 3 hours (standard procedure for measuring [ATP] in this manuscript), 5 hours and 7 hours. We did not observe a significant difference in ATP concentrations between 3 and 5 hours. However, when comparing 3 hours and 7 hours (* $P < 0.016$, two-tailed t-test), significant differences were observed. The reduction at this time point is likely due to entry into stationary phase.

**F)** Growth productivity when *E. coli* is grown in 0.04% glucose or 0.04% acetate when [ATP] is measured at 3 hours, 5 hours, and 7 hours. Growth productivity of glucose is always less than growth productivity for acetate. Error bars = standard error from linear regression used to determine growth productivity. Standard error at 7 hours is larger likely owing to entry into stationary phase; we currently do not understand how growth productivity, or our method of quantification, would be affected under these conditions.

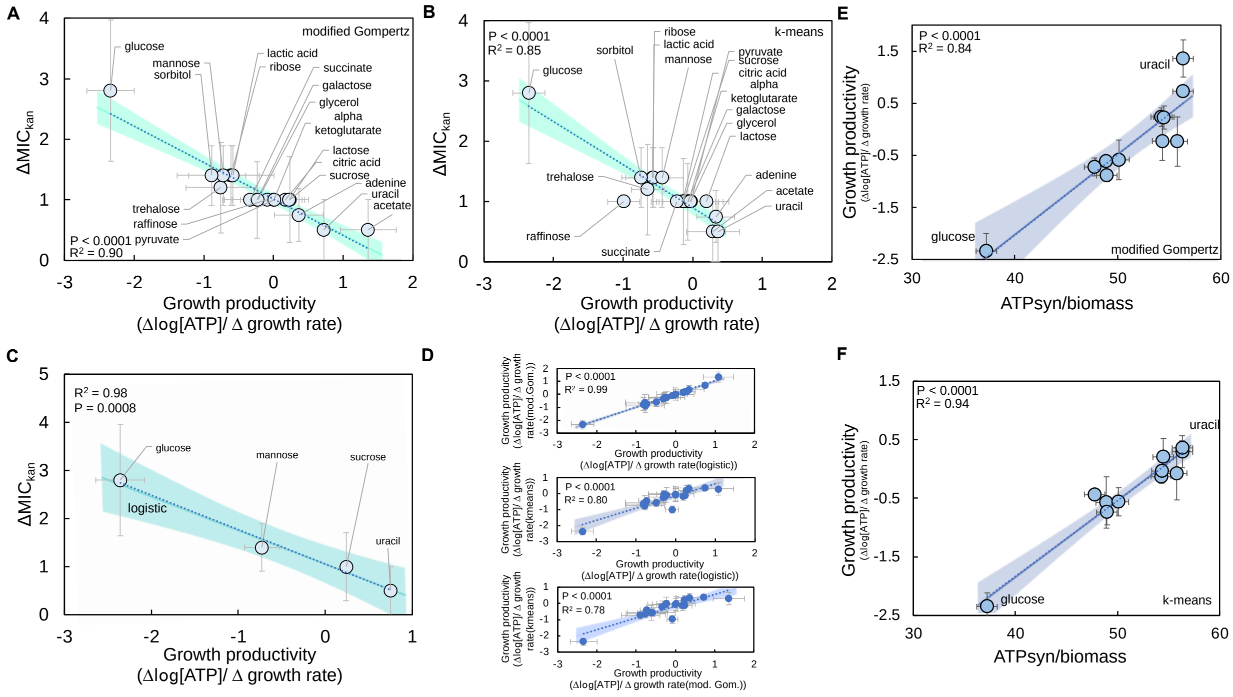


**Fig. S15. The relationship between $\Delta MIC_{kan}$ and growth productivity when maximum growth rate is determined using a modified Gompertz or k-means clustering analysis.**

**A)** The relationship between $\Delta MIC_{kan}$ and growth productivity when maximum growth rate is determined using a modified Gompertz equation. ΔMIC replotted from Fig. 7C. For all panels, shading indicates 95% confidence interval. P value and $R^2$ shown on plot are from a simple linear regression (see Table S9 for additional outputs from this regression). Deming regression: $P < 0.0001$ (see Table S10 for additional outputs from this regression).

**B)** The relationship between $\Delta MIC_{kan}$ and growth productivity when maximum growth rate is determined using a k-means clustering analysis. P value and $R^2$ shown on plot are from a simple linear regression (see Table S9 for additional outputs from this regression). Deming regression: $P < 0.0001$ (see Table S10 for additional outputs from this regression). WLS regression: $P < 0.0001$ (see Table S11 for additional outputs from this regression).

**C)** The relationship between $\Delta MIC_{kan}$ and growth productivity using only growth productivity values where the relationship between [ATP] and growth rate (logistic equation) was significant (Table S7). P value and $R^2$ shown on plot are from a simple linear regression (see Table S9 for additional outputs from this regression). Deming regression: P = 0.008 (Table S10). WLS regression: P = 0.027 (Table S11).

**D)** The relationship between growth productivity values when maximum growth rate is calculated using either a logistic equation, a modified Gompertz equation and a k-means clustering analysis. P value and $R^2$ shown on plot are from a simple linear regression. Deming regressions: logistic vs mod. Gom. P < 0.0001, k-means vs logistic P < 0.0001, k-means vs. mod Gom. P < 0.0001.

**E)** Correlation between FBA predicted values of ATP synthase activity/biomass and growth productivity when maximum growth rate is determined using a modified Gompertz. Simple linear regression, P = 0.0002. For panels E and F, data points corresponding to glucose and uracil are indicated to aid in comparison between FBA predicted and experimentally observed growth productivity values.

**F)** Correlation between FBA predicted values of ATP synthase activity/biomass and growth productivity when maximum growth rate is determined using a k-means clustering analysis. Simple linear regression, P < 0.0001.

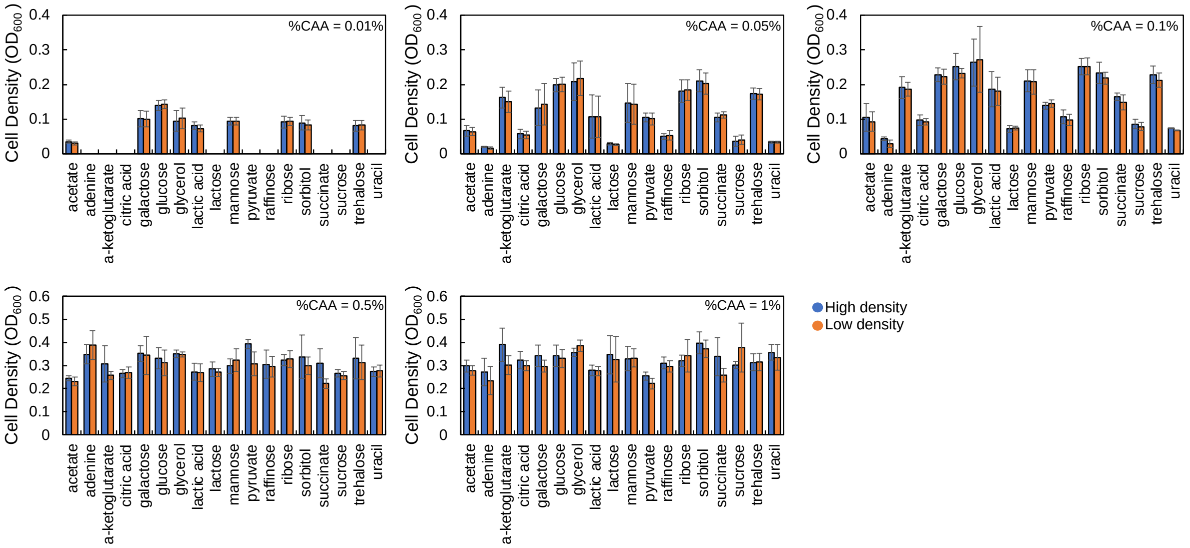


**Fig. S16. Growth of high and low initial density populations in the absence of antibiotics.** Carbon source indicated along the x-axis are provided in the growth medium at 0.04%. We observed that both high and low initial density populations reach approximately the same cell density ($OD_{600}$) after 24 hours of growth in our experimental setup to measure MIC. This indicates that the growth medium provides sufficient nutrients to allow growth of both the high- and low-density populations despite their difference in initial starting density. $P > 0.05$ (two-tailed t-test) for all comparisons with each carbon source and percentage of casamino acids (CAA) with the exception of galactose with 1% casamino acids ($P = 0.020$), pyruvate with 0.5% ($P = 0.013$) and 1% ($P = 0.022$) casamino acids, ribose with 1% casamino acids ($P = 0.015$) and succinate with 0.5% ($P =0.042$) casamino acids.

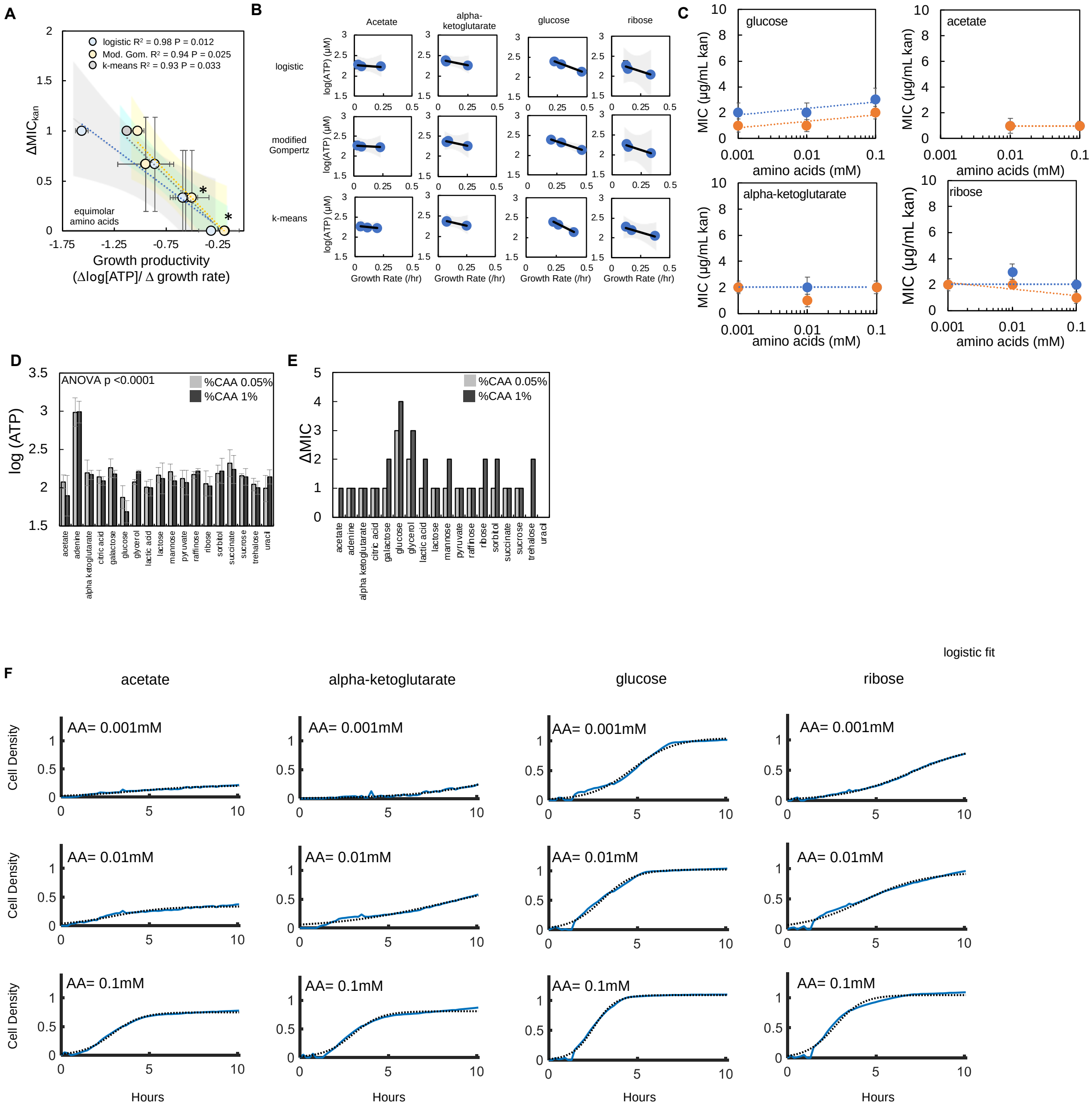
A
logistic $R^2$ = 0.98 P = 0.012
Mod. Gom. $R^2$ = 0.94 P = 0.025
k-means $R^2$ = 0.93 P = 0.033
equimolar amino acids
$\Delta MIC_{kan}$
Growth productivity
(Δlog[ATP]/ Δ growth rate)
B
Acetate
alpha-ketoglutarate
glucose
ribose
logistic
modified Gompertz
k-means
log(ATP) (µM)
Growth Rate (/hr)
C
glucose
acetate
alpha-ketoglutarate
ribose
MIC (µg/mL kan)
amino acids (mM)
D
ANOVA p <0.0001
%CAA 0.05%
%CAA 1%
log (ATP)
acetate
adenine
alpha ketoglutarate
citric acid
galactose
glucose
glycerol
lactic acid
lactose
mannose
pyruvate
raffinose
ribose
sorbitol
succinate
sucrose
trehalose
uracil
E
%CAA 0.05%
%CAA 1%
ΔMIC
F
logistic fit
acetate
alpha-ketoglutarate
glucose
ribose
AA= 0.001mM
AA= 0.01mM
AA= 0.1mM
Cell Density
Hours

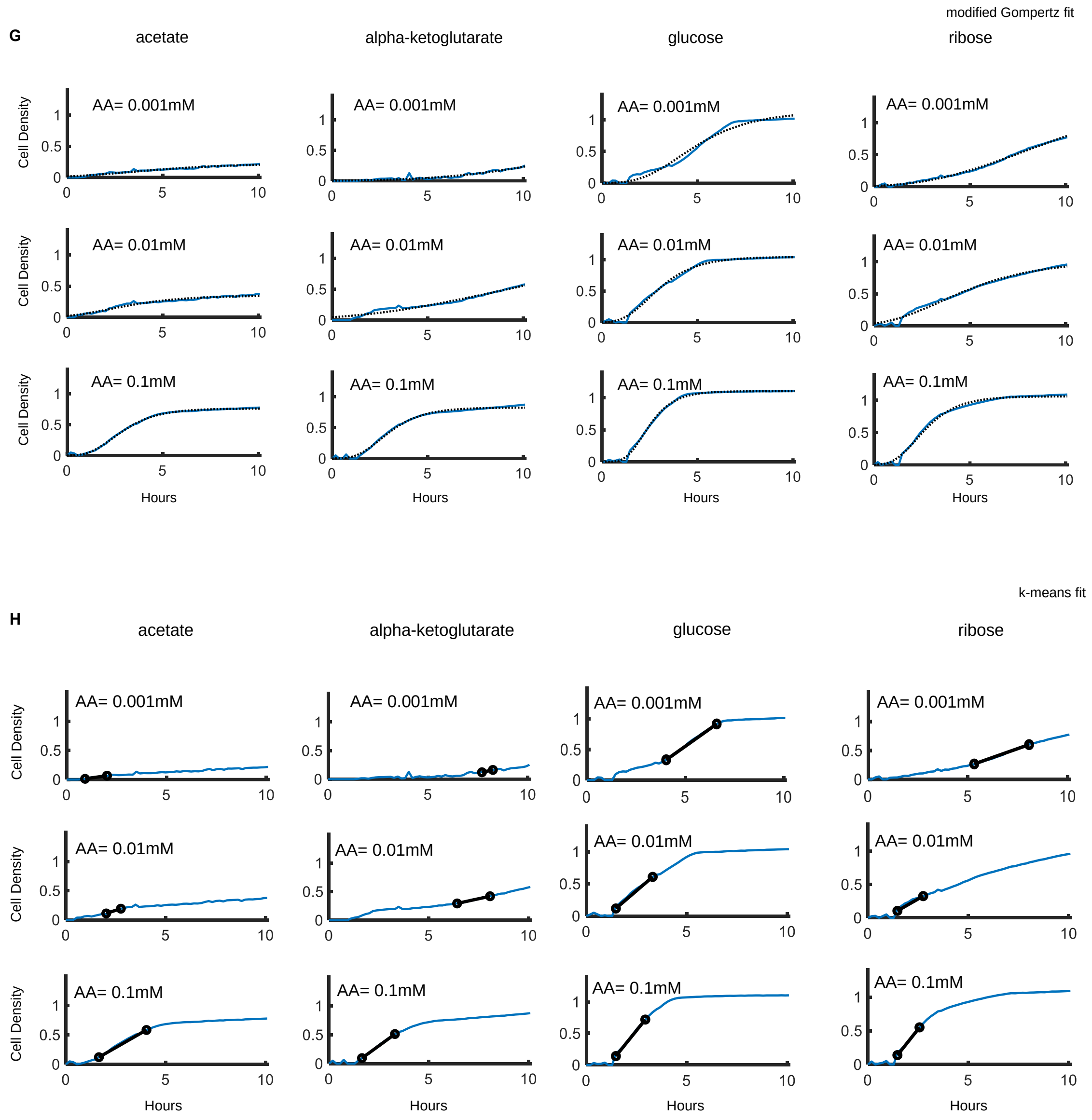


**Figure S17. Using equimolar concentrations of amino acids produces qualitatively similar trends between ΔMIC$_{kan}$ and growth productivity.**

**A)** The relationship between ΔMIC$_{kan}$ and growth productivity of *E. coli* when using equimolar concentrations of amino acids (carbon source = 0.04%). Standard deviation from a minimum of two different concentrations of amino acids, each consisting of six biological replicates. * indicates that ΔMIC is no different than zero ($P = 0.22$ and $P = 1$ for alpha-ketoglutarate and acetate, respectively, one-tailed t-tests). Maximum growth rate calculated using a logistic

equation (blue), a modified Gompertz equation (yellow) or a k-means clustering analysis (grey). $R^2$ and P values from a simple linear regression included on the plot (additional outputs from this test in Table S9). Deming regression: P = 0.004, 0.015 and 0.01 for growth productivity determined using a logistic equation, modified Gompertz equation and k-means clustering analysis (additional outputs from Deming regression in Table S10). WLS regression: P = 0.004 and 0.005 for growth productivity determined using a logistic equation and k-means clustering analysis (additional outputs from WLS in Table S11). Error bars: x-axis = standard error from linear regression used to determine growth productivity, y-axis = standard deviation.

**B)** The concentration of ATP plotted as a function of maximum growth rate. [ATP] measured from three biological replicates each consisting of two technical replicates. Maximum growth rate measured from the average of six biological replicates. Error bars = standard deviation. Shading = 95% confidence interval.

**C)** Raw MIC data for *E. coli* grown in equimolar concentration of amino acids. Standard deviation from a minimum of three biological replicates.

**D)** The concentration of ATP as a function of the percentage of casamino acids (CAA) for each carbon source. Standard deviation from a minimum of three biological replicates, each consisting of a minimum of two technical replicates.

**E)** ΔMIC as a function of the percentage of casamino acids (CAA) for each carbon source. $\Delta MIC_{kan}$ determined from the average $OD_{600}$ values of a minimum of three biological replicates.

**F)** Growth curves fit using a logistic equation (Eq. S1). For all panels solid line = experimental data and dotted lined = fit using logistic equation. To determine cell density, $OD_{600}$ values were log-transformed and normalized to the initial minimum density. Average plotted from a minimum of three biological replicates. Average residual values for each growth curve fit with

a logistic equation and modified Gompertz equation are found in Table S3. AA = amino acids. For all sub panels y = cell density, x = time in hours.

**G)** Growth curves fit using a modified Gompertz equation (Eq. S2).

**H)** Growth curves fit using a k-means clustering analysis. Solid black line indicates longest region of fastest growth. Black circles indicate beginning and end point.

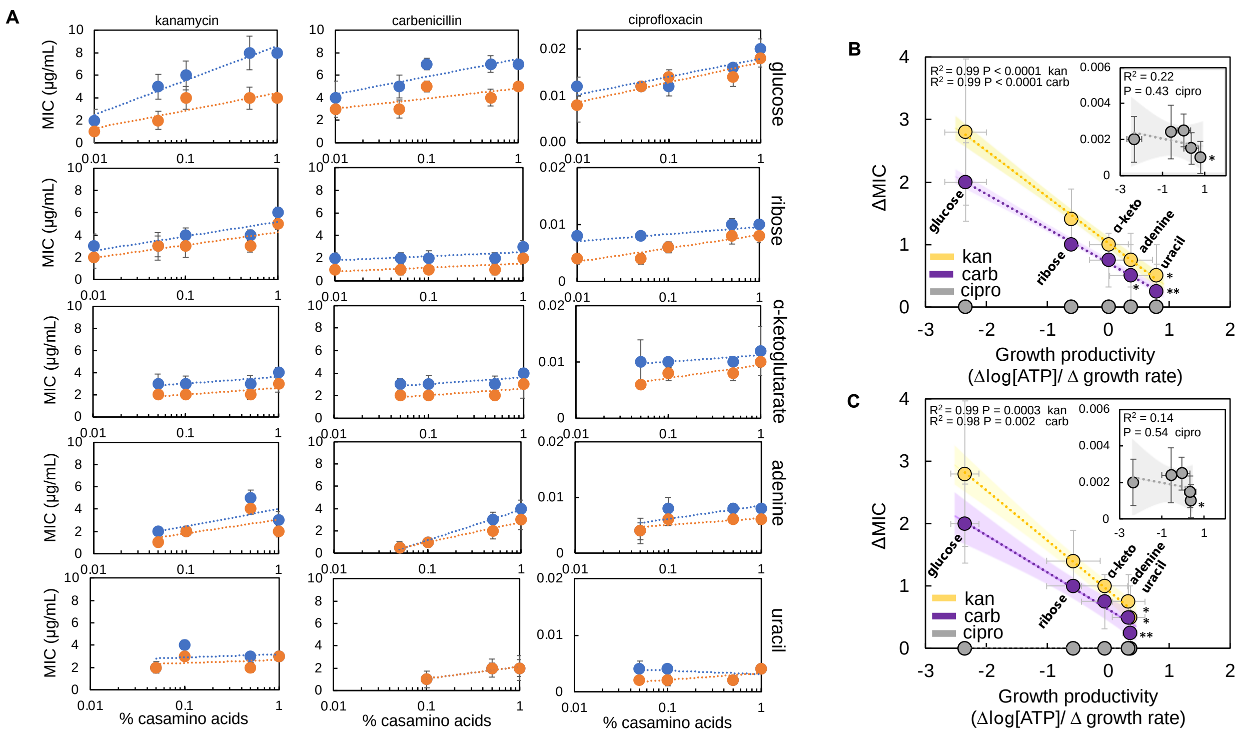

**Fig. S18. Raw MIC data for Fig. 8 and alternative plots of ΔMIC vs. growth productivity for carbenicillin and ciprofloxacin.**

- **A)** MIC of *E. coli* grown in kanamycin (kan), carbenicillin (carb) and ciprofloxacin (cipro) with glucose (0.04%), ribose (0.04%), α-ketoglutarate (0.04%), adenine (0.04%), and uracil (0.04%). Standard deviation from a minimum of three biological replicates. Lines indicate general trend in the data. Blue circles = high density; orange circles = low density.
- **B)** The relationship between ΔMIC and growth productivity when a modified Gompertz equation (Eq. S2) is used to determine maximum growth rate. ΔMIC data replotted from Fig. 8. P values and $R^2$ from simple linear regression indicated on the plot. In panels B and C, shading indicates 95% confidence interval. * indicates ΔMIC is not greater than zero (* P = 0.091, ** P = 0.21, one-tailed t-test). Deming regression: P < 0.0001, P < 0.0001, and P = 0.43 for kan, carb and cipro, respectively (additional outputs in Table S10).

**C)** The relationship between ΔMIC and growth productivity when a k-means clustering analysis is used to determine maximum growth rate. ΔMIC data replotted from Fig. 8. P values and $R^2$ from simple linear regression indicated on the plot. Deming regression: P = 0.001, P = 0.004, and P = 0.63 for kan, carb and cipro, respectively (additional outputs in Table S10). WLS regression: P = 0.0004, P = 0.0012, P = 0.31 for kan, carb and cipro, respectively (additional outputs in Table S11).

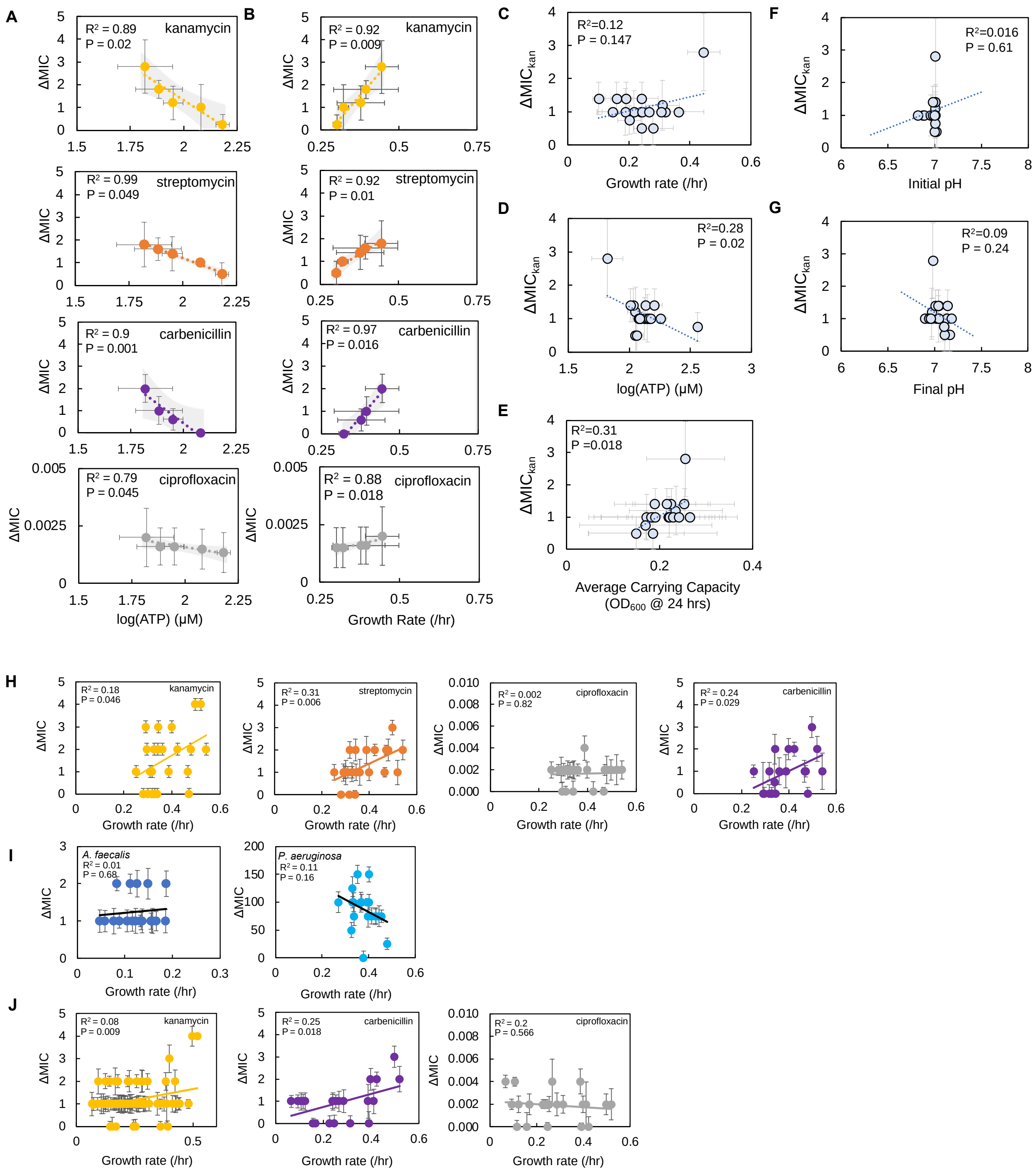
A
B
C
D
E
F
G
H
I
J
R² = 0.89
P = 0.02
kanamycin
R² = 0.92
P = 0.009
R²=0.12
P = 0.147
R²=0.016
P = 0.61
R² = 0.99
P = 0.049
streptomycin
R² = 0.92
P = 0.01
R²=0.28
P = 0.02
R²=0.09
P = 0.24
R² = 0.9
P = 0.001
carbenicillin
R² = 0.97
P = 0.016
R²=0.31
P =0.018
R² = 0.79
P = 0.045
ciprofloxacin
R² = 0.88
P = 0.018
ΔMIC
ΔMICkan
log(ATP) (μM)
Growth Rate (/hr)
Growth rate (/hr)
Initial pH
Final pH
Average Carrying Capacity
(OD600 @ 24 hrs)
R² = 0.18
P = 0.046
R² = 0.31
P = 0.006
R² = 0.002
P = 0.82
R² = 0.24
P = 0.029
A. faecalis
R² = 0.01
P = 0.68
P. aeruginosa
R² = 0.11
P = 0.16
R² = 0.08
P = 0.009
R² = 0.25
P = 0.018
R² = 0.2
P = 0.566

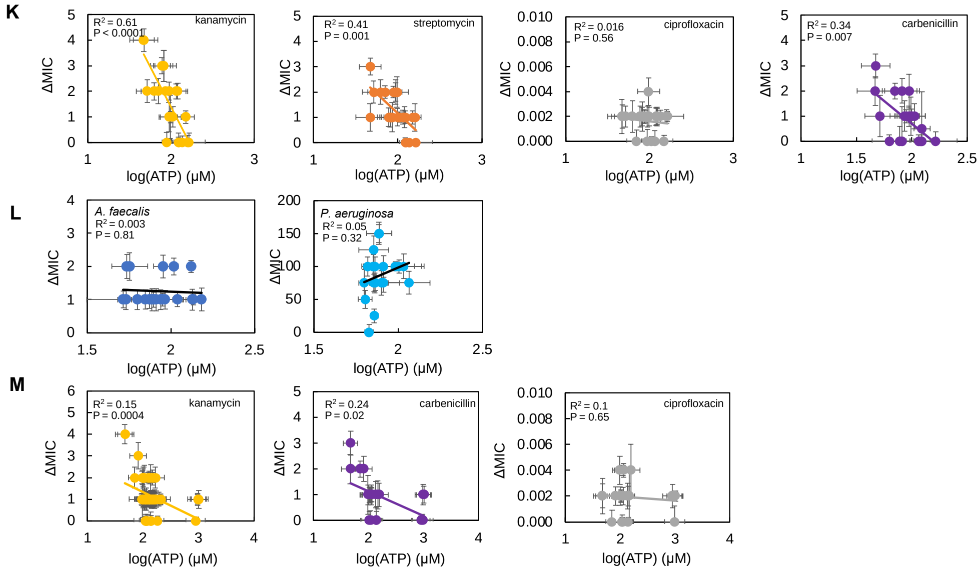


**Fig. S19. Exploring the relationship between ΔMIC and variables other than growth productivity.**

**A)** ΔMIC for kanamycin, carbenicillin, ciprofloxacin, and streptomycin plotted as a function of average ATP concentration across percentages of casamino acids (glucose: 0.04%). For all panels in this figure, P values and $R^2$ values from a simple linear regression indicated on the plots. Standard deviation from a minimum of three biological replicates. For panels A and B, shading indicates 95% confidence interval.

**B)** ΔMIC plotted as a function of maximum growth rate (determined using a logistic curve). Maximum growth rates averaged across percentages of casamino acids (glucose: 0.04%). Standard deviation from a minimum of three biological replicates.

**C)** $\Delta MIC_{kan}$ plotted as a function of average maximum growth rate for each carbon source tested in Fig. 7C. Maximum growth rates averaged across a minimum of four concentrations of casamino acids. Standard deviation from > three biological replicates (for both ΔMIC and growth rate).

**D)** $\Delta MIC_{kan}$ plotted as a function of ATP concentration for each carbon source tested in Fig. 7C. ATP concentration averaged across a minimum of four concentrations of casamino acids. Standard deviation from > three biological replicates. ATP measurements also consisted of ≥ two technical replicates.

**E)** $\Delta MIC_{kan}$ plotted as a function of carrying capacity for each carbon source. Carrying capacity measured without antibiotics and averaged across a minimum of four concentrations of casamino acids. Standard deviation from > three biological replicates.

**F)** $\Delta MIC_{kan}$ plotted as a function of initial pH for each carbon source. Standard deviation from three biological replicates.

**G)** $\Delta MIC_{kan}$ plotted as a function of final pH for each carbon source. Standard deviation from three biological replicates.

**H)** ΔMIC of kanamycin, streptomycin, carbenicillin and ciprofloxacin plotted as a function of maximum growth rate (logistic equation) for each percentage of glucose and casamino acids measured. ΔMIC values replotted from Fig. 2 and Fig. S4. For panels H-M, $R^2$ and P value on plot are from a standard linear regression. $P = 0.02$, $R^2 = 0.24$ when only carbon sources and kanamycin from Fig. 7C are used.

**I)** $\Delta MIC_{kan}$ for *A. faecalis* and *P. aeruginosa* plotted as a function of maximum growth rate (logistic equation) for each percentage of glucose and casamino acids measured. ΔMIC values replotted from Fig. S10.

**J)** ΔMIC of kanamycin, carbenicillin and ciprofloxacin plotted as a function of maximum growth rate (logistic equation) for carbon source (0.04%) and casamino acids measured. $\Delta MIC_{kan}$ values from Fig. S18.

**K)** ΔMIC of kanamycin, streptomycin, carbenicillin and ciprofloxacin plotted as a function of [ATP] for each percentage of glucose and casamino acids measured. ΔMIC values replotted from Fig. 2. $P = 0.012$, $R^2 = 0.27$ when only carbon sources and kanamycin from Fig. 7C are used.

**L)** ΔMIC of kanamycin for *A. faecalis* and *P. aeruginosa* plotted as a function of [ATP] for each percentage of glucose and casamino acids measured. ΔMIC values replotted from Fig. 6.

**M)** ΔMIC of kanamycin, carbenicillin and ciprofloxacin plotted as a function of [ATP] for carbon source (0.04%) and casamino acids measured. ΔMIC values from Fig. S18.

**SM Tables**

**Table S1.** Select case reports and clinical studies where IE has been suspected of causing failure of antibiotic treatment.

| Source | Finding | Ref |
|---|---|---|
| Patients with bacteremia | Treatment failure and persistent *S. aureus* infections associated with strains with IE. | (e.g., (*79*)) |
| Patients with endocarditis | Treatment with linezolid did not resolve infection despite *S. aureus* cultures showing standard sensitivity. Linezolid has been shown to produce IE. | (e.g., (*80*)) |
| Multiple patients with a variety of infections | Treatment failure with vancomycin despite showing vancomycin susceptibility in *in vitro* testing. IE + multi-focal infections suggested as the cause. | (*81*) |
| Neutropenic patients | Fecal anaerobes from patients showed IE with norfloxacin. Suggests that IE results in failure of norfloxacin to selectively decontaminate the bowel. | (*82*) |
| Patients with bacteremia and endocarditis | *In vitro* study of *S. aureus* isolates showed IE with cephalosporins. IE suggested as possible treatment failure. | (*83*) |
| Patients with endocarditis and septic arthritis | Group G strep. isolated from patients showed IE to penicillin-G and tolerance to additional antibiotics. IE suggested as reason for treatment failure. | (*84*) |
| Patients with various infections | Carbapenemase-negative *K. pneumoniae* and *E. coli* showed IE for ceftazidime-avibactam and aztreonam-avibactam. IE suggested as reason for treatment failure. | (*85*) |

**Table S2.** The highest density of bacteria recovered from patients from select studies. Fold increase over performance standard for antimicrobial testing is indicated in brackets.

| Source | Bacteria | Density in CFU/mL (fold increase) | Ref |
|---|---|---|---|
| Performance Standard for Antimicrobial Testing | Multiple species | $5 \times 10^5$ (1) | (*31*) |
| Cerebrospinal fluid specimens | Multiple species | $4 \times 10^9$ (8000 X) | (*86*) |
| Intra-abdominal and soft tissue | Multiple species | $2 \times 10^8$ (400 X) | (*87*) |
| Cholesteatoma samples | Multiple species | $> 10^6$ (> 2 X) | (*88*) |
| CF sputum samples | *Haemophilus influenzae* | $7.7 \times 10^8$ (1540 X) | (*89*) |
| | *Pseudomonas aeruginosa* | $8.0 \times 10^8$ (1600 X) | |
| | *Staphylococcus aureus* | $7.1 \times 10^8$ (1420 X) | |
| | *Branhamella catarrhalis* | $1.07 \times 10^9$ (2140 X) | |
| Chronic wounds | Multiple species | $5 \times 10^8$ (1000 X) | (*90*) |

**Table S3. Average residual values for growth curve fitting.**

| Bacteria | Carbon Source | % | logistic fit | | | | | modified Gompertz fit | | | | |
|---|---|---|---|---|---|---|---|---|---|---|---|---|
| % casamino acids | | | 0.01 | 0.05 | 0.1 | 0.5 | 1 | 0.01 | 0.05 | 0.1 | 0.5 | 1 |
| *E. coli* | Glucose | 0.4 | $9.8 \times 10^{-3}$ | $7.1 \times 10^{-3}$ | $7.5 \times 10^{-3}$ | $8.0 \times 10^{-3}$ | $7.7 \times 10^{-3}$ | $4.4 \times 10^{-3}$ | $8.4 \times 10^{-4}$ | $1.7 \times 10^{-3}$ | $1.8 \times 10^{-3}$ | $6.3 \times 10^{-4}$ |
| | | 0.04 | $4.5 \times 10^{-3}$ | $2.5 \times 10^{-3}$ | $3.6 \times 10^{-3}$ | $5.1 \times 10^{-3}$ | $2.6 \times 10^{-3}$ | $-1.3 \times 10^{-5}$ | $-2.4 \times 10^{-3}$ | $-7.5 \times 10^{-4}$ | $2.1 \times 10^{-4}$ | $-2.1 \times 10^{-3}$ |
| | | 0.004 | $2.6 \times 10^{-3}$ | $6.4 \times 10^{-3}$ | $6.5 \times 10^{-3}$ | $8.0 \times 10^{-3}$ | $7.1 \times 10^{-3}$ | $5.4 \times 10^{-4}$ | $2.3 \times 10^{-3}$ | $2.3 \times 10^{-3}$ | $3.6 \times 10^{-3}$ | $3.9 \times 10^{-3}$ |
| | | 0.0004 | | $2.3 \times 10^{-3}$ | $8.2 \times 10^{-3}$ | $8.6 \times 10^{-3}$ | $7.4 \times 10^{-3}$ | | $-1.7 \times 10^{-3}$ | $3.2 \times 10^{-3}$ | $4.1 \times 10^{-3}$ | $2.8 \times 10^{-3}$ |
| | | 0.00004 | | $1.5 \times 10^{-3}$ | $6.4 \times 10^{-3}$ | $7.1 \times 10^{-3}$ | $6.1 \times 10^{-3}$ | | $-1.9 \times 10^{-3}$ | $1.2 \times 10^{-3}$ | $1.9 \times 10^{-3}$ | $1.5 \times 10^{-3}$ |
| *E. coli* + 42ºC | | 0.04 | $8.4 \times 10^{-4}$ | $3.5 \times 10^{-3}$ | $5.7 \times 10^{-3}$ | $5.5 \times 10^{-3}$ | $5.1 \times 10^{-3}$ | $-6.2 \times 10^{-4}$ | $1.8 \times 10^{-3}$ | $2.4 \times 10^{-3}$ | $2.3 \times 10^{-3}$ | $1.8 \times 10^{-3}$ |
| *E. coli* + inhibitor | | | $-4.8 \times 10^{-3}$ | $6.6 \times 10^{-3}$ | $5.5 \times 10^{-3}$ | $5.1 \times 10^{-3}$ | $5.1 \times 10^{-3}$ | $-1.1 \times 10^{-2}$ | $2.7 \times 10^{-3}$ | $1.6 \times 10^{-3}$ | $1.2 \times 10^{-3}$ | $1.5 \times 10^{-3}$ |
| Δg*ltA* | | | | $5.8 \times 10^{-3}$ | $7.5 \times 10^{-3}$ | $8.8 \times 10^{-3}$ | $7.0 \times 10^{-3}$ | | $9.9 \times 10^{-4}$ | $2.2 \times 10^{-3}$ | $3.9 \times 10^{-3}$ | $2.3 \times 10^{-3}$ |
| Δ*cydB* | | | | $4.3 \times 10^{-3}$ | $7.3 \times 10^{-3}$ | $8.0 \times 10^{-3}$ | $1.0 \times 10^{-2}$ | | $1.5 \times 10^{-3}$ | $2.3 \times 10^{-3}$ | $3.2 \times 10^{-3}$ | $4.6 \times 10^{-3}$ |
| | | | | | | | | | | | | |
| *A. faecalis* | Glucose | 0.4 | | $8.5 \times 10^{-4}$ | $3.4 \times 10^{-3}$ | $3.6 \times 10^{-3}$ | $4.7 \times 10^{-3}$ | | $1.3 \times 10^{-3}$ | 2.0E $\times 10^{-3}$ | $2.0 \times 10^{-3}$ | $3.1 \times 10^{-3}$ |
| | | 0.04 | | $2.4 \times 10^{-3}$ | $3.0 \times 10^{-3}$ | $3.7 \times 10^{-3}$ | $3.6 \times 10^{-3}$ | | $1.0 \times 10^{-3}$ | $1.7 \times 10^{-3}$ | $1.9 \times 10^{-3}$ | $1.5 \times 10^{-3}$ |
| | | 0.004 | | $2.3 \times 10^{-3}$ | $3.6 \times 10^{-3}$ | $5.1 \times 10^{-3}$ | $6.0 \times 10^{-3}$ | | 8.5E-04 | $1.6 \times 10^{-3}$ | $2.9 \times 10^{-3}$ | $3.0 \times 10^{-3}$ |
| | | 0.0004 | | $1.8 \times 10^{-3}$ | $3.6 \times 10^{-3}$ | $4.7 \times 10^{-3}$ | $7.0 \times 10^{-3}$ | | -2.9E-04 | $1.4 \times 10^{-3}$ | $1.9 \times 10^{-3}$ | $3.7 \times 10^{-3}$ |
| | | 0.00004 | | $1.6 \times 10^{-3}$ | $2.6 \times 10^{-3}$ | $4.8 \times 10^{-3}$ | $6.1 \times 10^{-3}$ | | $-2.4 \times 10^{-3}$ | $1.3 \times 10^{-4}$ | $1.8 \times 10^{-3}$ | $2.8 \times 10^{-3}$ |
| *P. aeruginosa* | | 0.4 | | $3.6 \times 10^{-3}$ | $4.9 \times 10^{-3}$ | $8.1 \times 10^{-3}$ | $7.9 \times 10^{-3}$ | | 3.9E-04 | $1.1 \times 10^{-3}$ | $3.6 \times 10^{-3}$ | $3.3 \times 10^{-3}$ |
| | | 0.04 | | $3.1 \times 10^{-3}$ | $5.7 \times 10^{-3}$ | $7.5 \times 10^{-3}$ | $8.3 \times 10^{-3}$ | | -7.9E-04 | $1.6 \times 10^{-3}$ | $3.4 \times 10^{-3}$ | $4.2 \times 10^{-3}$ |
| | | 0.004 | | $5.1 \times 10^{-3}$ | $5.4 \times 10^{-3}$ | $9.1 \times 10^{-3}$ | $1.1 \times 10^{-2}$ | | $1.5 \times 10^{-3}$ | $9.1 \times 10^{-4}$ | $4.4 \times 10^{-3}$ | $5.7 \times 10^{-3}$ |
| | | 0.0004 | | $5.9 \times 10^{-3}$ | $6.3 \times 10^{-3}$ | $9.6 \times 10^{-3}$ | $9.1 \times 10^{-3}$ | | $2.3 \times 10^{-3}$ | $2.6 \times 10^{-3}$ | $4.9 \times 10^{-3}$ | $4.6 \times 10^{-3}$ |
| | | 0.00004 | | $6.7 \times 10^{-3}$ | $5.6 \times 10^{-3}$ | $1.1 \times 10^{-2}$ | $1.2 \times 10^{-3}$ | | $2.5 \times 10^{-3}$ | $1.2 \times 10^{-3}$ | $5.8 \times 10^{-3}$ | $6.4 \times 10^{-3}$ |
| *K. pneumoniae* | | 0.04 | $2.2 \times 10^{-3}$ | | $2.2 \times 10^{-3}$ | | $4.5 \times 10^{-3}$ | $-1.9 \times 10^{-4}$ | | $-6.0 \times 10^{-4}$ | | $2.1 \times 10^{-3}$ |
| *A. baumannii* | | | $1.1 \times 10^{-3}$ | | $8.1 \times 10^{-3}$ | | $8.1 \times 10^{-3}$ | $2.9 \times 10^{-3}$ | | $4.0 \times 10^{-3}$ | | $5.6 \times 10^{-3}$ |

| | | | | | | | | | | | | |
|---|---|---|---|---|---|---|---|---|---|---|---|---|
| *P. aeruginosa* | acetate | 0.004 | | 6.2 x $10^{-3}$ | 7.8 x $10^{-3}$ | 9.9 x $10^{-3}$ | | | | | | |
| | | | | | | | | | | | | |
| *E. coli* | Acetate | 0.04 | | 3.6 x $10^{-3}$ | 5.1 x $10^{-3}$ | 6.5 x $10^{-3}$ | 6.5 x $10^{-3}$ | | 7.1 x $10^{-4}$ | 1.7 x $10^{-3}$ | 3.9 x $10^{-3}$ | 3.8 x $10^{-3}$ |
| | Adenine | | | 3.3 x $10^{-3}$ | 2.2 x $10^{-3}$ | 6.1 x $10^{-3}$ | 5.8 x $10^{-3}$ | | 2.6 x $10^{-3}$ | 1.1 x $10^{-3}$ | 1.2 x $10^{-3}$ | 1.1 x $10^{-3}$ |
| | α-keto glutarate | | | 6.5 x $10^{-3}$ | 7.9 x $10^{-3}$ | 8.0 x $10^{-3}$ | 8.2 x $10^{-3}$ | | 3.8 x $10^{-3}$ | 4.6 x $10^{-3}$ | 4.9 x $10^{-3}$ | 4.5 x $10^{-3}$ |
| | Citric acid | | 2.2 x $10^{-4}$ | 1.1 x $10^{-3}$ | 3.2 x $10^{-3}$ | 7.9 x $10^{-3}$ | 5.0 x $10^{-3}$ | 1.8 x $10^{-4}$ | -1.4 x $10^{-4}$ | 1.3 x $10^{-3}$ | 4.8 x $10^{-3}$ | 3.3 x $10^{-3}$ |
| | Galactose | | 6.3 x $10^{-3}$ | 8.2 x $10^{-3}$ | 4.6 x $10^{-3}$ | 9.5 x $10^{-3}$ | 6.0 x $10^{-3}$ | 3.5 x $10^{-3}$ | 3.7 x $10^{-3}$ | 2.7 x $10^{-3}$ | 6.1 x $10^{-3}$ | 3.8 x $10^{-3}$ |
| | Glycerol | | 4.8 x $10^{-3}$ | 6.0 x $10^{-3}$ | 3.7 x $10^{-3}$ | 9.0 x $10^{-3}$ | 7.4 x $10^{-3}$ | 3.0 x $10^{-3}$ | 3.6 x $10^{-3}$ | 2.2 x $10^{-3}$ | 5.9 x $10^{-3}$ | 4.3 x $10^{-3}$ |
| | Lactic acid | | -1.2 x $10^{-3}$ | 6.0 x $10^{-3}$ | 5.9 x $10^{-3}$ | 8.0 x $10^{-3}$ | 8.1 x $10^{-3}$ | 2.7 x $10^{-3}$ | 3.8 x $10^{-3}$ | 3.9 x $10^{-3}$ | 5.1 x $10^{-3}$ | 5.1 x $10^{-3}$ |
| | Lactose | | 3.0 x $10^{-4}$ | -1.3 x $10^{-3}$ | 5.7 x $10^{-3}$ | 6.1 x $10^{-3}$ | 5.9 x $10^{-3}$ | 2.4 x $10^{-4}$ | -3.5 x $10^{-3}$ | 2.4 x $10^{-3}$ | 3.4 x $10^{-3}$ | 3.1 x $10^{-3}$ |
| | Mannose | | 3.7 x $10^{-3}$ | 5.3 x $10^{-3}$ | 5.7 x $10^{-3}$ | 8.3 x $10^{-3}$ | 7.0 x $10^{-3}$ | 2.2 x $10^{-3}$ | 3.0 x $10^{-3}$ | 3.4 x $10^{-3}$ | 4.7 x $10^{-3}$ | 4.1 x $10^{-3}$ |
| | Pyruvate | | | 5.3 x $10^{-3}$ | 5.7 x $10^{-3}$ | 7.5 x $10^{-3}$ | 7.6 x $10^{-3}$ | | 1.1 x $10^{-3}$ | 2.6 x $10^{-3}$ | 3.4 x $10^{-3}$ | 4.8 x $10^{-3}$ |
| | Raffinose | | 1.6 x $10^{-4}$ | 1.7 x $10^{-3}$ | 5.0 x $10^{-3}$ | 7.7 x $10^{-3}$ | 6.3 x $10^{-3}$ | 1.2 x $10^{-4}$ | 2.1 x $10^{-5}$ | 2.3 x $10^{-3}$ | 4.7 x $10^{-3}$ | 3.4 x $10^{-3}$ |
| | Ribose | | -7.5 x $10^{-4}$ | 4.0 x $10^{-4}$ | 2.6 x $10^{-3}$ | 9.1 x $10^{-4}$ | 3.1 x $10^{-3}$ | -2.3 x $10^{-3}$ | -1.0 x $10^{-3}$ | 3.9 x $10^{-4}$ | -1.7x $10^{-3}$ | 1.5 x $10^{-3}$ |
| | Sorbitol | | 3.6 x $10^{-3}$ | 3.3 x $10^{-3}$ | 6.3 x $10^{-3}$ | 7.8 x $10^{-3}$ | 5.0 x $10^{-3}$ | 1.5 x $10^{-3}$ | 1.2 x $10^{-3}$ | 3.4 x $10^{-3}$ | 4.4 x $10^{-3}$ | 2.1 x $10^{-3}$ |
| | Succinate | | | 7.0 x $10^{-3}$ | 1.0E x $10^{-2}$ | 7.9 x $10^{-3}$ | 7.3 x $10^{-3}$ | | 4.2 x $10^{-3}$ | 5.9 x $10^{-3}$ | 4.3 x $10^{-3}$ | 3.3 x $10^{-3}$ |
| | Sucrose | | 6.0 x $10^{-4}$ | -2.1 x $10^{-4}$ | 5.4 x $10^{-3}$ | 5.3 x $10^{-3}$ | 7.0 x $10^{-3}$ | 4.8 x $10^{-4}$ | -2.2 x $10^{-3}$ | 3.4 x $10^{-3}$ | 2.3 x $10^{-3}$ | 4.3 x $10^{-3}$ |
| | Trehalose | | 2.7 x $10^{-3}$ | 5.4 x $10^{-3}$ | 5.3 x $10^{-3}$ | 6.2 x $10^{-3}$ | 6.5 x $10^{-3}$ | -2.8 x $10^{-4}$ | 2.3 x $10^{-3}$ | 2.1 x $10^{-3}$ | 3.1 x $10^{-3}$ | 3.7 x $10^{-3}$ |
| | Uracil | | | -1.1 x $10^{-3}$ | 4.1 x $10^{-3}$ | 5.2 x $10^{-3}$ | 8.6 x $10^{-3}$ | | -2.9 x $10^{-3}$ | -8.1 x $10^{-4}$ | 4.2 x $10^{-4}$ | 4.6 x $10^{-3}$ |
| | | | | | | | | | | | | |
| Equimolar [amino acids] | | | 0.001mM | 0.01mM | 0.1mM | | | 0.001mM | 0.01mM | 0.1mM | | |
| *E. coli* | Acetate | 0.04% | 1.1 x $10^{-3}$ | 1.93 x $10^{-3}$ | 2.32 x $10^{-3}$ | | | 7.65 x $10^{-4}$ | 1.07 x $10^{-3}$ | -9.01x $10^{-4}$ | | |
| | α-keto glutarate | | 6.9 x $10^{-5}$ | 2.2 x $10^{-3}$ | 3.9 x $10^{-3}$ | | | -8.7 x $10^{-4}$ | 1.7 x $10^{-3}$ | 1.7 x $10^{-4}$ | | |
| | Glucose | | -1.9 x $10^{-3}$ | 4.6 x $10^{-3}$ | 3.6 x $10^{-3}$ | | | 5.2 x $10^{-3}$ | 5.9 x $10^{-4}$ | 4.7 x $10^{-4}$ | | |
| | Ribose | | 9.6 x $10^{-4}$ | 5.01 x $10^{-3}$ | 5.9 x $10^{-3}$ | | | -2.9 x $10^{-4}$ | 2.8 x $10^{-3}$ | 1.5 x $10^{-3}$ | | |

**Table S4. Base parameters used in our mathematical model (Eq. 1, Methods, main text).** Changes to these parameter values are noted in the figure legend of the corresponding simulation results.

| Parameter | Value | Reference |
|---|---|---|
| Growth rate ($\mu$) | 0.6/hr | Estimated from growth rate data as described in "*Parameter estimation.*" |
| Metabolism ($\varepsilon$) | .055 mmol/g/hr | Estimated as described in "*Parameter estimation*" and from (*70*). |
| Antibiotic-specific death rate (*b*) | .1/hr | Estimated from (*21*). |
| Half maximal killing rate of the antibiotic (*K*) | .1 | Estimated |
| Cell density (*N)* | $N_{high}$ =0.05<br>$N_{low}$ = 0.0001 | Scaled to carrying capacity of M9 medium as described in "*Parameter estimation*". |
| Antibiotic concentration (*A*) | 0-1 (unitless) | As indicate in each panel |
| Time (*t*) | 24 hr | Experimental conditions |
| Carrying capacity ($N_m$) | 1 (unitless) | Normalized to N |

**Table S5. Upper and lower bound flux values of carbon sources and additional nutrients used in our flux balance analysis.**

| Reaction | Lower bound | Standard Deviation | Upper bound | Ref |
|---|---|---|---|---|
| **Core reactions** | | | | |
| Glycine | -0.8835861 | 0.181034596 | 1000 | (*73*) |
| Alanine | -1.682609333 | 0.072240506 | 1000 | (*73*) |
| Arginine | -1.6722119 | 0.065289227 | 1000 | (*73*) |
| Asparagine | -2.396529233 | 0.030236646 | 1000 | (*73*) |
| Aspartate | 0.895297803 | 1.25699554 | 1000 | (*73*) |
| Cysteine | -1.4220841 | 2.011130621 | 1000 | (*73*) |
| Glutamate | -1.5974812 | 0.542145573 | 1000 | (*73*) |
| Glutamine | -1.987637733 | 0.079369847 | 1000 | (*73*) |
| Histidine | -3.3186743 | 0.201010114 | 1000 | (*73*) |
| Isoleucine | -2.090270167 | 0.15705341 | 1000 | (*73*) |
| Leucine | -2.090270167 | 0.15705341 | 1000 | (*73*) |
| Lysine | -2.177075267 | 0.370693518 | 1000 | (*73*) |
| Methionine | -2.431328733 | 0.081043021 | 1000 | (*73*) |
| Phenylalanine | -2.422301767 | 0.206049329 | 1000 | (*73*) |
| Proline | -2.3791969 | 0.322196029 | 1000 | (*73*) |
| Serine | -0.403842533 | 0.058898371 | 1000 | (*73*) |
| Threonine | -1.907954967 | 0.402042504 | 1000 | (*73*) |
| Tryptophan | -3.014569033 | 0.205088312 | 1000 | (*73*) |
| Tyrosine | -3.524060967 | 0.499361877 | 1000 | (*73*) |
| Valine | -2.266623633 | 0.167116189 | 1000 | (*73*) |
| Thiamine | -0.000001 | | 1000 | (*71*) |
| Oxygen | -20 | | 1000 | (*74*) |
| **Carbon sources** | | | | |
| Glucose | -12 | | 1000 | (*91*) |
| Acetate | -2.77 | | 1000 | (*92*) |
| Lactic acid (lactate) | -13.7 | | 1000 | (*93*) |
| Glycerol | -2.01 | | 1000 | (*94*) |
| Lactose | -0.36 | | 1000 | (*95*) |
| Galactose | -0.207 | | 1000 | (*96*) |
| Sucrose | -0.4157 | | 1000 | (*97*) |
| Mannose | -7 | | 1000 | (*98*) |
| Sorbitol | -4.68 | | 1000 | (*99*) |
| Ribose | 8.45 | | 1000 | (*100*) |
| Uracil | -4.61538 x $10^{6}$ | | 1000 | (*101*) |

**Table S6. Antibiotic concentrations gradients used in MIC experiments (5,000-fold - 50,000-fold).** For each bacteria and antibiotic, we chose the smallest gradient that would lead to an observable difference between high density and low density populations. In some instances, not all antibiotic concentrations were tested for each experiment. For example, if an MIC was established for both high and low density populations (e.g., both populations do not grow at concentrations greater than 8 μg/mL of kanamycin), higher concentrations of antibiotics (e.g., 12 μg/mL and 15 μg/mL of kanamycin) were no longer tested. Similarly, if it was clear that both high and low density populations were both growing at low concentrations of antibiotics (e.g., 2 μg/mL of kanamycin), lower concentrations (0.5 μg/mL and 1 μg/mL of kanamycin) were not tested.

| Carbon source | Antibiotic | Stock concentration (μg/mL) | Range of antibiotic concentrations tested (μg/mL) |
|---|---|---|---|
| *E. coli* | | | |
| Glucose and all other metabolites | kanamycin | 500 | 0, 0.5, 1, 2, 3,4, 5, 6,7, 8, 10, 12, 15 |
| | carbenicillin | 1000 | 0, 0.5, 1, 2, 3, 4, 5, 6, 7, 8, 9, 10 |
| | ciprofloxacin | 5 and 0.5 | 0, 0.002, 0.004, 0.006, 0.008, 0.01, 0.012, 0.014, 0.016, 0.01, 0.02, 0.022, 0.025 |
| | streptomycin | 500 | 0, 1, 2, 3, 4, 5, 6, 7, 8 |
| *P. aeruginosa* | | | |
| glucose | kanamycin | 5,000 | 0, 75, 100, 125 , 150, 175, 200 , 225, 250, 275, 300, 325 |
| acetate | | | |
| *A. faecalis* | | | |
| glucose | kanamycin | 500 | 0, 2, 3, 4, 5, 6, 7, 8, 9, 10, 11, 12 |
| *K. pneumoniae* | | | |
| glucose | kanamycin | 50,000 | 0, 100, 125, 150, 175 200, 225, 250, 275, 300, 325, 350, 375, 400, 425, 450, 475, 500, 525, 550, 575, 600 |
| *A. baumannii* | | | |
| glucose | kanamycin | 50,000 | 0, 400, 500, 600, 700, 800, 900, 1000, 1100, 1200, 1300, 1400, 1500, 1600, 1700, 1800 |

**Table S7.** Summary statistics from simple linear regression performed between log(ATP) and growth rate throughout the manuscript.

| | Figure | Logistic | | | | | Figure | ModGom | | | | | Figure | K-means | | | | |
|---|---|---|---|---|---|---|---|---|---|---|---|---|---|---|---|---|---|---|
| | | Slope | Std Error | P value | Lower 95% | Upper 95% | | Slope | Std Error | P value | Lower 95% | Upper 95% | | Slope | Std Error | P value | Lower 95% | Upper 95% |
| **Metabolite** | | | | | | | | | | | | | | | | | | |
| **Alpha - ketoglutarate** | Fig. S14 | -0.01 | 0.30 | 0.988 | -1.30 | 1.29 | Fig. S14 | 0.01 | 0.32 | 0.986 | -1.37 | 1.38 | Fig. S14 | -0.03 | 0.38 | 0.93 | -1.65 | 1.58 |
| **Acetate** | Fig. S14 | 1.08 | 0.38 | 0.10 | -0.53 | 2.7 | Fig. S14 | 1.36 | 0.40 | 0.078 | -0.37 | 3.09 | Fig. S14 | 0.29 | 0.38 | 0.52 | -1.35 | 1.94 |
| **Adenine** | Fig. S14 | 0.33 | 0.39 | 0.485 | -1.34 | 1.99 | Fig. S14 | 0.37 | 0.35 | 0.398 | -1.13 | 1.87 | Fig. S14 | 0.32 | 0.27 | 0.36 | -0.84 | 1.49 |
| **Citric acid** | Fig. S14 | 0.19 | 0.07 | 0.125 | -0.13 | 0.50 | Fig. S14 | 0.18 | 0.07 | 0.120 | -0.11 | 0.46 | Fig. S14 | 0.14 | 0.29 | 0.67 | -1.11 | 1.39 |
| **Galactose** | Fig. S14 | -0.23 | 0.46 | 0.658 | -1.70 | 1.25 | Fig. S14 | -0.23 | 0.47 | 0.650 | -1.72 | 1.25 | Fig. S14 | -0.07 | 0.48 | 0.88 | -1.5 | 1.35 |
| **Glucose** | | | | | | | | | | | | | | | | | | |
| **Glucose 0.00004** | Fig. 1C | 2.00 | 0.83 | 0.14 | -1.58 | 5.59 | Fig. S5 | 1.68 | 0.67 | 0.13 | -1.24 | 4.59 | Fig. S5 | -0.27 | 0.46 | 0.61 | -2.24 | 1.69 |
| **Glucose 0.0004** | Fig. 1C | 0.67 | 0.15 | 0.047 | 0.02 | 1.32 | Fig. S5 | 0.57 | 0.18 | 0.083 | -0.19 | 1.33 | Fig. S5 | -0.20 | 0.10 | 0.186 | -0.65 | 0.24 |
| **Glucose 0.004** | Fig. 1C | 0.35 | 0.29 | 0.32 | -0.59 | 1.28 | Fig. S5 | 0.33 | 0.26 | 0.29 | -0.49 | 1.16 | Fig. S5 | -0.33 | 0.43 | 0.5 | -1.69 | 1.03 |
| **Glucose 0.04** | Fig. 1C | -2.36 | 0.28 | 0.004 | -3.24 | -1.47 | Fig. S5 | -2.33 | 0.34 | 0.007 | -3.43 | -1.24 | Fig. S5 | -2.35 | 0.23 | 0.002 | -3.07 | -1.64 |
| **Glucose 0.4** | Fig. 1C | -1.03 | 0.14 | 0.005 | -1.48 | -0.59 | Fig. S5 | -1.01 | 0.13 | 0.005 | -1.44 | -0.58 | Fig. S5 | -1.49 | -0.19 | 0.005 | -2.11 | -0.87 |
| **Glucose + 42ºC** | Fig. S9 | -0.34 | 0.09 | 0.036 | -0.63 | -0.04 | Fig. S9 | -0.37 | 0.09 | 0.030 | -0.67 | -0.07 | | | | | | |
| **Glucose + PMSF** | Fig. S9 | -0.46 | 0.17 | 0.075 | -1.00 | 0.09 | Fig. S9 | -0.46 | 0.17 | 0.078 | -1.02 | 0.10 | | | | | | |
| **Glycerol** | Fig. S14 | -0.28 | 0.40 | 0.53 | -1.55 | 0.98 | Fig. S14 | -0.23 | 0.37 | 0.59 | -1.42 | 0.97 | Fig. S14 | -0.03 | 0.21 | 0.90 | -0.71 | 0.65 |
| **Lactic acid** | Fig. S14 | -0.49 | 0.24 | 0.131 | -1.25 | 0.27 | Fig. S14 | -0.59 | 0.38 | 0.225 | -1.81 | 0.64 | Fig. S14 | -0.56 | 0.24 | 0.10 | -1.32 | 0.19 |
| **Lactose** | Fig. S14 | 0.28 | 0.21 | 0.31 | -0.62 | 1.18 | Fig. S14 | 0.23 | 0.2 | 0.37 | -0.63 | 1.09 | Fig. S14 | 0.2 | 0.32 | 0.59 | -1.17 | 1.58 |
| **Mannose** | Fig. S14 | -0.73 | 0.20 | 0.035 | -1.36 | -0.10 | Fig. S14 | -0.72 | 0.17 | 0.024 | -1.26 | -0.18 | Fig. S14 | -0.44 | 0.08 | 0.01 | -0.68 | -0.20 |
| **Pyruvate** | Fig. S14 | 0.00 | 0.47 | 0.995 | -2.04 | 2.04 | Fig. S14 | 0.02 | 0.49 | 0.976 | -2.10 | 2.13 | Fig. S14 | -0.02 | 0.46 | 0.96 | -1.98 | 1.94 |
| **Raffinose** | Fig. S14 | -0.09 | 0.16 | 0.630 | -0.76 | 0.59 | Fig. S14 | -0.08 | 0.14 | 0.621 | -0.69 | 0.52 | Fig. S14 | -0.99 | 0.23 | 0.049 | -1.97 | -0.02 |
| **Ribose** | Fig. S14 | -0.78 | 0.49 | 0.209 | -2.35 | 0.78 | Fig. S14 | -0.61 | 0.6 | 0.39 | -2.54 | 1.32 | Fig. S14 | -0.57 | 0.44 | 0.28 | -1.98 | 0.83 |
| **Sorbitol** | Fig. S14 | -0.77 | 0.49 | 0.213 | -2.33 | 0.78 | Fig. S14 | -0.89 | 0.49 | 0.17 | -2.44 | 0.67 | Fig. S14 | -0.74 | 0.22 | 0.046 | -1.46 | 0.03 |
| **Succinate** | Fig. S14 | -0.31 | 0.37 | 0.489 | -1.89 | 1.27 | Fig. S14 | -0.33 | 0.40 | 0.491 | -2.03 | 1.37 | Fig. S14 | -0.23 | 0.37 | 0.60 | -1.83 | 1.38 |
| **Sucrose** | Fig. S14 | 0.28 | 0.05 | 0.04 | 0.04 | 0.44 | Fig. S14 | 0.23 | 0.04 | 0.03 | 0.05 | 0.42 | Fig. S14 | -0.08 | 1.25 | 0.95 | -5.49 | 5.32 |
| **Trehalose** | Fig. S14 | -0.81 | 0.46 | 0.18 | -2.27 | 0.65 | Fig. S14 | -0.75 | 0.5 | 0.23 | -2.34 | 0.83 | Fig. S14 | -0.65 | 0.24 | 0.07 | -1.4 | 0.11 |
| **Uracil** | Fig. S14 | 0.75 | 0.07 | 0.01 | 0.43 | 1.07 | Fig. S14 | 0.73 | 0.06 | 0.01 | 0.47 | 0.99 | Fig. S14 | 0.36 | 0.12 | 0.1 | -0.15 | 0.87 |

| | | | | | | | | | | | | | | | | | | |
|---|---|---|---|---|---|---|---|---|---|---|---|---|---|---|---|---|---|---|
| ***∆gltA*** | App Fig. S6 | 0.11 | 0.42 | 0.82 | -1.70 | 1.91 | Fig. S9 | 0.16 | 0.31 | 0.65 | -1.17 | 1.49 | | | | | | |
| ***∆cydB*** | Fig. S9 | -1.03 | 0.15 | 0.022 | -1.69 | -0.37 | Fig. S9 | 0.78 | 0.15 | 0.036 | 0.13 | 1.44 | | | | | | |
| | | | | | | | | | | | | | | | | | | |
| ***P. aeruginosa*** | | | | | | | | | | | | | | | | | | |
| **Glucose** | | | | | | | | | | | | | | | | | | |
| **Glucose 0.00004** | Fig. 6 | 0.42 | 0.42 | 0.423 | -1.37 | 2.20 | | | | | | | | | | | | |
| **Glucose 0.0004** | Fig. 6 | -0.35 | 0.40 | 0.47 | -2.07 | 1.37 | | | | | | | | | | | | |
| **Glucose 0.004** | Fig. 6 | -1.66 | 0.81 | 0.18 | -5.17 | 1.84 | | | | | | | | | | | | |
| **Glucose 0.04** | Fig. 6 | -0.01 | 0.48 | 0.98 | -2.07 | 2.05 | | | | | | | | | | | | |
| **Glucose 0.4** | Fig. 6 | -0.72 | 0.59 | 0.34 | -3.25 | 1.81 | | | | | | | | | | | | |
| **Acetate 0.004** | Fig. S10 | -0.12 | 0.09 | 0.39 | -1.23 | 0.99 | | | | | | | | | | | | |
| | | | | | | | | | | | | | | | | | | |
| ***K. pneumoniae*** | | | | | | | | | | | | | | | | | | |
| **Glucose 0.04** | Fig. S10 | -0.36 | 0.39 | 0.52 | -5.31 | 4.58 | | | | | | | | | | | | |
| | | | | | | | | | | | | | | | | | | |
| ***A. faecalis*** | | | | | | | | | | | | | | | | | | |
| **Glucose 0.00004** | Fig. 6 | -1.26 | 0.60 | 0.17 | -3.85 | 1.34 | | | | | | | | | | | | |
| **Glucose 0.0004** | Fig. 6 | -3.41 | 0.33 | 0.009 | -4.84 | -1.99 | | | | | | | | | | | | |
| **Glucose 0.004** | Fig. 6 | -3.62 | 1.44 | 0.13 | -9.82 | 2.58 | | | | | | | | | | | | |
| **Glucose 0.04** | Fig. 6 | -5.35 | 0.68 | 0.016 | -8.29 | -2.4 | | | | | | | | | | | | |
| **Glucose 0.4** | Fig. 6 | -3.43 | 1.02 | 0.08 | -7.8 | 0.95 | | | | | | | | | | | | |
| | | | | | | | | | | | | | | | | | | |
| ***A. baumannii*** | | | | | | | | | | | | | | | | | | |
| **Glucose 0.04** | Fig. S10 | -0.63 | 0.07 | 0.068 | -1.49 | 0.23 | | | | | | | | | | | | |
| | | | | | | | | | | | | | | | | | | |
| ***E. coli* (with equimolar amino acids)** | | | | | | | | | | | | | | | | | | |
| **Acetate** | Fig. S17 | -0.18 | 0.14 | 0.42 | -1.97 | 1.6 | Fig. S17 | -0.19 | 0.14 | 0.41 | -1.95 | 1.58 | Fig. S17 | -0.31 | 0.12 | 0.23 | -1.82 | 1.19 |
| **Alpha-ketoglutarate** | Fig. S17 | -0.56 | 0.12 | 0.13 | -2.15 | 0.97 | Fig. S17 | -0.58 | 0.16 | 0.17 | -2.64 | 1.49 | Fig. S17 | -0.59 | 0.12 | 0.13 | -2.15 | 0.97 |
| **Glucose** | Fig. S17 | -1.13 | 0.02 | 0.01 | -1.36 | -0.89 | Fig. S17 | -1.02 | 0.06 | 0.04 | -1.77 | -0.27 | Fig. S17 | -1.56 | 0.06 | 0.026 | -2.36 | -0.76 |

| | | | | | | | | | | | | | | | | | |
|---|---|---|---|---|---|---|---|---|---|---|---|---|---|---|---|---|---|
| **Ribose** | Fig. S17 | -0.94 | 0.27 | 0.18 | -4.41 | 2.52 | Fig. S17 | -0.94 | 0.27 | 0.18 | -4.41 | 2.53 | Fig. S17 | -0.86 | 0.14 | 0.10 | -2.66 | 0.94 |

**Table S8.** Summary statistics from simple linear regression performed between log(luminescence of $NAD^+$/NADH) and growth rate.

| % Glucose | Figure | Logistic | | | | |
|---|---|---|---|---|---|---|
| | | Slope | Std Error | P value | Lower 95% | Upper 95% |
| 0.00004 | Fig. S6B | 17.37 | 0.54 | 0.001 | 15.06 | 19.69 |
| 0.0004 | | 11.8 | 1.26 | 0.011 | 6.33 | 17.19 |
| 0.004 | | 5.96 | 0.46 | 0.001 | 4.48 | 7.42 |
| 0.04 | | -6.97 | 0.13 | <0.0001 | -7.38 | -6.55 |
| 0.4 | | -0.55 | 0.12 | 0.02 | -0.93 | -0.16 |

**Table S9.** Summary statistics from a simple linear regression performed between ΔMIC and growth productivity throughout the manuscript.

| Mechanism to calculate maximum growth rate | Figure | Antibiotic | Estimate | Std Error | $R^2$ | P value | Lower 95% | Upper 95% |
|---|---|---|---|---|---|---|---|---|
| | | | | | | | | |
| Logistic | Fig. 2C | Kanamycin | -0.55 | 0.07 | 0.95 | 0.005 | -0.79 | -0.32 |
| | | Streptomycin | -0.34 | 0.02 | 0.99 | 0.0003 | -0.4 | -0.28 |
| | | Carbenicillin | -0.59 | 0.11 | 0.94 | 0.028 | -1.02 | -0.15 |
| | | Ciprofloxacin | -0.0001 | 0.00003 | 0.88 | 0.018 | -0.00023 | -4.53E-5 |
| Modified Gompertz | Fig. S5 | Kanamycin | -0.59 | 0.09 | 0.94 | 0.007 | -0.87 | 0.31 |
| | | Streptomycin | -0.37 | 0.03 | 0.98 | 0.001 | -0.46 | -0.27 |
| | | Carbenicillin | -0.6 | 0.11 | 0.94 | 0.031 | -1.08 | -0.13 |
| | | Ciprofloxacin | -0.0001 | 3.0E-5 | 0.89 | 0.016 | -0.0002 | -5.2E-5 |
| k-means clustering | Fig. S5 | Kanamycin | -0.85 | 0.2 | 0.85 | 0.026 | -1.5 | -0.19 |
| | | Streptomycin | -0.53 | 0.1 | 0.91 | 0.012 | -0.83 | -0.22 |
| | | Carbenicillin | -0.79 | 0.18 | 0.91 | 0.045 | -1.53 | -0.04 |
| | | Ciprofloxacin | -0.0002 | 5.7E-5 | 0.83 | 0.033 | -0.0004 | -3.3E-6 |
| | | | | | | | | |
| Logistic* | Fig. 7C | Kanamycin | -0.63 | 0.05 | 0.90 | <0.0001 | -0.74 | -0.52 |
| modified Gompertz# | Fig. S15 | Kanamycin | -0.60 | 0.05 | 0.89 | <0.0001 | -0.72 | -0.49 |
| k-means clustering^ | Fig. S15 | Kanamycin | -0.71 | 0.08 | 0.85 | <0.0001 | -0.88 | -0.55 |
| | | | | | | | | |
| Logistic (equimolar) | Fig. S17 | Kanamycin | -1.01 | 0.11 | 0.98 | 0.012 | -1.49 | -0.54 |
| modified Gompertz (equimolar) | Fig. S17 | Kanamycin | -1.06 | 0.19 | 0.94 | 0.029 | -1.86 | -0.27 |
| k-means clustering (equimolar) | Fig. S17 | Kanamycin | -0.78 | 0.15 | 0.93 | 0.033 | -1.40 | -0.15 |
| | | | | | | | | |
| Logistic | Fig. 6B (*A. faecalis*) | Kanamycin | -0.12 | 0.006 | 0.99 | 0.0003 | -0.14 | -0.10 |
| Logistic | Fig. 6B (*P. aeruginosa*) | Kanamycin | -33.04 | 6.98 | 0.88 | 0.018 | -55.25 | -10.83 |
| | | | | | | | | |

| | | | | | | | | |
|---|---|---|---|---|---|---|---|---|
| Logistic (P < 0.05 only) | Fig. S15 | Kanamycin | -0.72 | 0.07 | 0.98 | 0.008 | -1.0 | -0.44 |
| | | | | | | | | |
| Logistic | Fig. 8 | Kanamycin | -0.72 | 0.05 | 0.99 | 0.0008 | -0.89 | -0.56 |
| Logistic | Fig. 8 | Carbenicillin | -0.54 | 0.03 | 0.99 | 0.0004 | -0.64 | -0.44 |
| Logistic | Fig. 8 | Ciprofloxacin | -0.0003 | 0.00025 | 0.25 | 0.39 | -0.001 | 0.001 |
| | | | | | | | | |
| modified Gompertz | Fig. S18 | Kanamycin | -0.74 | 0.026 | 0.996 | < 0.0001 | -0.82 | -0.66 |
| modified Gompertz | Fig. S18 | Carbenicillin | -0.55 | 0.015 | 0.997 | <0.0001 | -0.6 | -0.5 |
| modified Gompertz | Fig. S18 | Ciprofloxacin | -0.0002 | 0.0003 | 0.22 | 0.43 | -0.001 | 0.0005 |
| | | | | | | | | |
| k-means clustering | Fig. S18 | Kanamycin | -0.80 | 0.04 | 0.99 | 0.0003 | -0.94 | -0.67 |
| k-means clustering | Fig. S18 | Carbenicillin | -0.59 | 0.05 | 0.98 | 0.0015 | -0.76 | -0.48 |
| k-means clustering | Fig. S18 | Ciprofloxacin | -0.0002 | 0.0003 | 0.14 | 0.54 | -0.001 | 0.0008 |

*Bootstrapping analysis using a linear regression: Significant ($t_{1,2499}$= -287.66, p<0.001, bootstrapped CIs -0.738<slope<-0.401)

#Bootstrapping analysis using a linear regression: Significant ($t_{1,2499}$= -263.09, p<0.001, bootstrapped CIs -0.747<slope<-0.387)

^Bootstrapping analysis using a linear regression: Significant ($t_{1,2499}$= -233.32, p<0.001, bootstrapped CIs -0.834<slope<-0.262)

**Table S10.** Summary statistics from Deming (Model II) linear regression performed between ΔMIC and growth productivity throughout the manuscript. We note that Deming regressions do not report $R^2$ values.

| Mechanism to calculate maximum growth rate | Figure | Antibiotic | Estimate | Std Error | P value | Lower 95% | Upper 95% |
|---|---|---|---|---|---|---|---|
| Logistic | Fig. 2C | Kanamycin | -0.57 | 0.03 | 0.005 | --0.68 | -0.46 |
| | | Streptomycin | -0.34 | 0.03 | 0.0003 | -0.44 | -0.25 |
| | | Carbenicillin | -0.61 | 0.08 | 0.028 | -0.97 | -0.25 |
| | | Ciprofloxacin | -0.0001 | 4.6E-5 | 0.018 | -0.0003 | -7.39E-7 |
| Modified Gompertz | Fig. S5 | Kanamycin | -0.61 | 0.06 | 0.007 | -0.8 | -0.42 |
| | | Streptomycin | -0.37 | 0.05 | 0.0012 | -0.54 | -0.20 |
| | | Carbenicillin | -0.63 | 0.09 | 0.03 | -1.02 | -0.23 |
| | | Ciprofloxacin | -0.0002 | 4.3E-5 | 0.02 | -0.0003 | 2.2E-5 |
| k-means clustering | Fig. S5 | Kanamycin | -0.92 | 0.2 | 0.026 | -1.6 | -0.27 |
| | | Streptomycin | -0.55 | 0.15 | 0.012 | -1.02 | -0.08 |
| | | Carbenicillin | -0.82 | 0.18 | 0.045 | -1.6 | -0.04 |
| | | Ciprofloxacin | -0.0002 | 6.6E-5 | 0.033 | -0.0004 | -3.1E-6 |
| | | | | | | | |
| Logistic | Fig. 7C | Kanamycin | -0.66 | 0.16 | <.0001 | -1.00 | -0.32 |
| modified Gompertz | Fig. S15 | Kanamycin | -0.64 | 0.18 | <.0001 | -1.02 | -0.26 |
| k-means clustering | Fig. S15 | Kanamycin | -0.78 | 0.11 | <0.001 | -1.02 | -0.55 |
| | | | | | | | |
| Logistic (equimolar) | Fig. S17 | Kanamycin | -1.02 | 0.18 | 0.012 | -1.81 | -0.24 |
| modified Gompertz (equimolar) | Fig. S17 | Kanamycin | -1.1 | 0.23 | 0.029 | -2.08 | -0.11 |
| k-means clustering (equimolar) | Fig. S17 | Kanamycin | -0.81 | 0.37 | 0.033 | -2.41 | 0.8 |
| | | | | | | | |
| Logistic | Fig. 6B (*A. faecalis*) | Kanamycin | -0.12 | 0.012 | 0.0003 | -0.16 | -0.08 |
| Logistic | Fig. 6B (*P. aeruginosa*) | Kanamycin | -35.2 | 15.9 | 0.018 | -85.82 | 15.44 |

| | | | | | | | |
|---|---|---|---|---|---|---|---|
| | | | | | | | |
| Logistic (P< 0.05 only) | Fig. S15 | Kanamycin | -0.72 | 0.10 | 0.008 | -1.16 | -0.29 |
| | | | | | | | |
| Logistic | Fig. 8 | Kanamycin | -0.73 | 0.14 | 0.0008 | -1.12 | -0.27 |
| Logistic | Fig. 8 | Carbenicillin | -0.54 | 0.06 | 0.0004 | -0.73 | -0.35 |
| Logistic | Fig. 8 | Ciprofloxacin | -0.0005 | 0.0007 | 0.39 | 0.00 | 0.00 |
| | | | | | | | |
| modified Gompertz | Fig. S18 | Kanamycin | -0.74 | 0.08 | <0.0001 | -1.00 | -0.48 |
| modified Gompertz | Fig. S18 | Carbenicillin | -0.55 | 0.009 | <0.0001 | -0.58 | -0.52 |
| modified Gompertz | Fig. S18 | Ciprofloxacin | -0.0005 | 0.0008 | 0.43 | -0.003 | 0.002 |
| | | | | | | | |
| k-means clustering | Fig. S18 | Kanamycin | -0.81 | 0.07 | 0.0003 | -0.10 | -0.58 |
| k-means clustering | Fig. S18 | Carbenicillin | -0.0005 | 0.001 | 0.54 | -0.005 | 0.004 |
| k-means clustering | Fig. S18 | Ciprofloxacin | -0.6 | 0.12 | 0.002 | -0.96 | -0.23 |

**Table S11.** Summary statistics from WLS regression performed between ΔMIC and growth productivity throughout the manuscript. Maximum growth rates determined using a logistic equation or k-means clustering analysis are shown. We chose to perform this analysis when maximum growth rate was estimated using a logistic equation and a k-means clustering analysis as they represent the two most divergent methods to determine maximum growth rate.

| Mechanism to calculate maximum growth rate | Figure | Antibiotic | Estimate | Std Error | $R^2$ | P value | Lower 95% | Upper 95% |
|---|---|---|---|---|---|---|---|---|
| Logistic | Fig. 2C | Kanamycin | -0.54 | 0.09 | 0.92 | 0.01 | -0.83 | -0.25 |
| | | Streptomycin | -0.33 | 0.019 | 0.99 | 0.0004 | -0.39 | -0.27 |
| | | Carbenicillin | -0.61 | 0.1 | 0.95 | 0.027 | -1.05 | -0.17 |
| | | Ciprofloxacin | -0.0001 | 0.00003 | 0.86 | 0.023 | -0.0003 | -3.82E-5 |
| k-means clustering | Fig. S5 | Kanamycin | -0.76 | 0.12 | 0.93 | 0.009 | -1.15 | -0.37 |
| | | Streptomycin | -0.47 | 0.04 | 0.98 | 0.001 | -0.60 | -0.34 |
| | | Carbenicillin | -0.86 | 0.12 | 0.96 | 0.019 | -1.38 | -0.34 |
| | | Ciprofloxacin | -0.0002 | 0.00006 | 0.77 | 0.052 | -0.0003 | 3.1E-6 |
| | | | | | | | | |
| Logistic | Fig. 6B (*A. faecalis*) | Kanamycin | -0.12 | 0.0004 | 0.997 | <0.0001 | -0.13 | -0.11 |
| | Fig. 6B (*P. aeruginosa*) | Kanamycin | -38.96 | 9.59 | 0.85 | 0.027 | -69.47 | -8.44 |
| | | | | | | | | |
| Logistic | Fig. 7C | Kanamycin | -0.63 | 0.06 | 0.89 | <0.0001 | -0.75 | -0.51 |
| K-means clustering | Fig. S15 | Kanamycin | -0.76 | 0.09 | 0.83 | <0.0001 | -0.95 | -0.58 |
| | | | | | | | | |
| Logistic (equimolar) | Fig. S17 | Kanamycin | -1.1 | 0.06 | 0.99 | 0.003 | -1.35 | -0.86 |
| K-means clustering (equimolar) | Fig. S17 | Kanamycin | -0.74 | 0.09 | 0.97 | 0.014 | -1.12 | -0.36 |
| | | | | | | | | |
| Logistic (P < 0.05 only) | Fig. S15 | Kanamycin | -0.71 | 0.12 | 0.95 | 0.027 | -1.21 | -0.2 |
| | | | | | | | | |
| Logistic | Fig. 8 | Kanamycin | -0.7 | 0.03 | 0.99 | 0.0002 | -0.81 | -0.59 |
| Logistic | Fig. 8 | Carbenicillin | -0.54 | 0.016 | 0.997 | <0.0001 | -0.6 | -0.49 |
| Logistic | Fig. 8 | Ciprofloxacin | -0.0004 | 0.0002 | 0.52 | 0.17 | -0.001 | 0.0003 |

| | | | | | | | | |
|---|---|---|---|---|---|---|---|---|
| k-means clustering | Fig. S18 | Kanamycin | -0.83 | 0.05 | 0.99 | 0.0004 | -.97 | -0.69 |
| k-means clustering | Fig. S18 | Carbenicillin | -0.63 | 0.05 | 0.98 | 0.0014 | -0.8 | -0.46 |
| k-means clustering | Fig. S18 | Ciprofloxacin | -0.0003 | 0.0002 | 0.38 | 0.27 | -0.001 | 0.0004 |

**Table S12.** Summary statistics from plots where $NAD^+$/NADH was used to determine growth productivity.

| Mechanism to calculate maximum growth rate | Figure | Type of Regression | Antibiotic | Estimate | Std Error | $R^2$ | P value | Lower 95% | Upper 95% |
|---|---|---|---|---|---|---|---|---|---|
| Logistic | Fig. S6A | Simple linear regression | Kanamycin | -0.1 | 0.011 | 0.96 | 0.003 | -0.13 | -0.06 |
| | | | Streptomycin | -0.06 | 0.005 | 0.98 | 0.0016 | -0.08 | -0.04 |
| | | | Carbenicillin | -0.1 | 0.013 | 0.97 | 0.016 | -0.16 | -0.05 |
| | | | Ciprofloxacin | -2.4E-5 | 5.5E-6 | 0.86 | 0.023 | -4.1 E-5 | -6.2 E-6 |
| Logistic | Fig. S6A | Deming regression | Kanamycin | -0.1 | 0.01002 | | 0.003 | -0.1302 | -0.06648 |
| | | | Streptomycin | -0.08 | 0.01541 | | <0.0001 | -0.1194 | -0.04831 |
| | | | Carbenicillin | -0.11 | 0.02742 | | 0.0043 | -0.1701 | -0.04046 |
| | | | Ciprofloxacin | -0.25 | 0.3311 | | 0.208 | -1.018 | 0.5092 |
| Logistic | Fig. S6A | WLS | Kanamycin | -0.12 | 0.02 | 0.92 | 0.01 | -0.18 | -0.05 |
| | | | Streptomycin | -0.13 | 0.02 | 0.95 | 0.024 | -0.23 | -0.04 |
| | | | Carbenicillin | -3.71E-5 | 1.2E-5 | 0.77 | 0.049 | -7.42E-5 | 1.98E-8 |
| | | | Ciprofloxacin | -0.06 | 0.004 | 0.99 | 0.0006 | -0.07 | -0.047 |

**Table S13.** Summary statistics from plots where a higher initial density of bacteria (500-fold dilution) was used to examine the relationship between growth productivity and ΔMIC.

| Mechanism to calculate maximum growth rate | Regression | Figure | Antibiotic | Estimate | Std Error | $R^2$ | P value | Lower 95% | Upper 95% |
|---|---|---|---|---|---|---|---|---|---|
| Logistic | Simple linear regression | Fig. S6E | Kanamycin | -1.41 | 0.24 | 0.94 | 0.028 | -2.45 | -0.37 |
| Modified Gompertz | | | | -1.54 | 0.23 | 0.96 | 0.022 | -2.55 | -0.53 |
| k-means clustering | | | | -2.33 | 0.45 | 0.93 | 0.036 | -4.29 | -0.39 |
| Logistic | WLS | | | -1.36 | 0.21 | 0.96 | 0.023 | -2.24 | -0.47 |
| Modified Gompertz | | | | -1.58 | 0.15 | 0.98 | 0.008 | -2.21 | -0.95 |
| k-means clustering | | | | -2.48 | 0.33 | 0.97 | 0.017 | -3.9 | -1.05 |
| Logistic | Deming regression | | | -1.452 | 0.3947 | | 0.028 | -3.15 | 0.25 |
| Modified Gompertz | | | | -1.574 | 0.3656 | | 0.022 | -3.15 | -0.00075 |
| k-means clustering | | | | -2.421 | 0.5374 | | 0.036 | -4.73 | -0.01086 |

## REFERENCES AND NOTES


1. S. B. Levy, B. Marshall, Antibacterial resistance worldwide: Causes, challenges and responses. *Nat. Med.* **10**, S122–S129 (2004).

2. Centers for Disease Control and Prevention, *Antibiotic Resistance Threats in the United States, 2019* (US Department of Health and Human Services, Centres for Disease Control and Prevention, 2019), p. 3.

3. K. E. Thorpe, P. Joski, K. J. Johnston, Antibiotic-resistant infection treatment costs have doubled since 2002, now exceeding $2 billion annually. *Health Aff.* **37**, 662–669 (2018).

4. B. Plackett, Why big pharma has abandoned antibiotics. *Nature* **586**, S50–S52 (2020).

5. J. M. Blair, M. A. Webber, A. J. Baylay, D. O. Ogbolu, L. J. Piddock, Molecular mechanisms of antibiotic resistance. *Nat. Rev. Microbiol.* **13**, 42–51 (2015).

6. M. J. Bottery, J. W. Pitchford, V.-P. Friman, Ecology and evolution of antimicrobial resistance in bacterial communities. *ISME J.* **15**, 939–948 (2021).

7. A. J. Lopatkin, S. Huang, R. P. Smith, J. K. Srimani, T. A. Sysoeva, S. Bewick, D. K. Karig, L. You, Antibiotics as a selective driver for conjugation dynamics. *Nat. Microbiol.* **1**, 16044 (2016).

8. Y. Tanouchi, A. Pai, N. E. Buchler, L. You, Programming stress-induced altruistic death in engineered bacteria. *Mol. Syst. Biol.* **8,** 626 (2012).

9. R. A. Sorg, L. Lin, G. S. Van Doorn, M. Sorg, J. Olson, V. Nizet, J. W. Veening, Collective resistance in microbial communities by intracellular antibiotic deactivation. *PLOS Biol.* **14**, e2000631 (2016).

10. H. R. Meredith, J. K. Srimani, A. J. Lee, A. J. Lopatkin, L. You, Collective antibiotic tolerance: Mechanisms, dynamics and intervention. *Nat. Chem. Biol.* **11**, 182–188 (2015).

11. I. Brook, Inoculum effect. *Rev. Infect. Dis.* **11**, 361–368 (1989).

12. D. Greenwood, Differentiation of mechanisms responsible for inoculum effects in the response of *Escherichia coli* to a variety of antibiotics. *J. Antimicrob. Chemother.* **2**, 87–95 (1976).

13. K. P. Smith, J. E. Kirby, The inoculum effect in the era of multidrug resistance: Minor differences in inoculum have dramatic effect on MIC determination. *Antimicrob. Agents Chemother.* **62**, e00433-18 (2018).

14. C. Tan, R. P. Smith, J. K. Srimani, K. A. Riccione, S. Prasada, M. Kuehn, L. You, The inoculum effect and band-pass bacterial response to periodic antibiotic treatment. *Mol. Syst. Biol.* **8**, 617 (2012).

15. J. Marchant, When antibiotics turn toxic. *Nature* **555**, 431–433 (2018).

16. J. Martinez, F. Baquero, Mutation frequencies and antibiotic resistance. *Antimicrob. Agents Chemother.* **44**, 1771–1777 (2000).

17. J.-C. Augustin, A. Brouillaud-Delattre, L. Rosso, V. Carlier, Significance of inoculum size in the lag time oflisteria monocytogenes. *Appl. Environ. Microbiol.* **66**, 1706–1710 (2000).

18. K. I. Udekwu, N. Parrish, P. Ankomah, F. Baquero, B. R. Levin, Functional relationship between bacterial cell density and the efficacy of antibiotics. *J. Antimicrob. Chemother.* **63**, 745–757 (2009).

19. A. Gutierrez, S. Jain, P. Bhargava, M. Hamblin, M. A. Lobritz, J. J. Collins, Understanding and sensitizing density-dependent persistence to quinolone antibiotics. *Mol. Cell* **68**, 1147–1154.e3 (2017).

20. J. R. Lenhard, Z. P. Bulman, Inoculum effect of β-lactam antibiotics. *J. Antimicrob. Chemother.* **74**, 2825–2843 (2019).

21. A. J. Lopatkin, J. M. Stokes, E. J. Zheng, J. H. Yang, M. K. Takahashi, L. You, J. J. Collins, Bacterial metabolic state more accurately predicts antibiotic lethality than growth rate. *Nat. Microbiol.* **4**, 2109–2117 (2019).

22. R. Mempin, H. Tran, C. Chen, H. Gong, K. Kim Ho, S. Lu, Release of extracellular ATP by bacteria during growth. *BMC Microbiol.* **13**, 1–13 (2013).

23. Y. Deng, D. R. Beahm, S. Ionov, R. Sarpeshkar, Measuring and modeling energy and power consumption in living microbial cells with a synthetic ATP reporter. *BMC Biol.* **19**, 1–21 (2021).

24. P. Pletnev, I. Osterman, P. Sergiev, A. Bogdanov, O. Dontsova, Survival guide: *Escherichia coli* in the stationary phase. *Acta Naturae* **7**, 22–33 (2015).

25. C. P. Kempes, S. Dutkiewicz, M. J. Follows, Growth, metabolic partitioning, and the size of microorganisms. *Proc. Natl. Acad. Sci. U.S.A.* **109**, 495–500 (2012).

26. M. Szenk, K. A. Dill, A. M. de Graff, Why do fast-growing bacteria enter overflow metabolism? Testing the membrane real estate hypothesis. *Cell Systems* **5**, 95–104 (2017).

27. W. P. Hempfling, S. E. Mainzer, Effects of varying the carbon source limiting growth on yield and maintenance characteristics of *Escherichia coli* in continuous culture. *J. Bacteriol.* **123**, 1076–1087 (1975).

28. A. G. Marr, Growth rate of *Escherichia coli*. *Microbiol. Rev.* **55**, 316–333 (1991).

29. M. Basan, S. Hui, H. Okano, Z. Zhang, Y. Shen, J. R. Williamson, T. Hwa, Overflow metabolism in *Escherichia coli* results from efficient proteome allocation. *Nature* **528**, 99–104 (2015).

30. A. Bren, J. O. Park, B. D. Towbin, E. Dekel, J. D. Rabinowitz, U. Alon, Glucose becomes one of the worst carbon sources for *E. coli* on poor nitrogen sources due to suboptimal levels of cAMP. *Sci. Rep.* **6**, 1–10 (2016).

31. M. A. Wikler, *Performance Standards for Antimicrobial Susceptibility Testing, Sixteenth Informational Supplement, M100-S16* (Clinical and Laboratory Standards Institute, 2006), vol. 26.

32. A. Dowling, J. O'Dwyer, C. Adley, Antibiotics: Mode of action and mechanisms of resistance. *Antimicrob. Res.* **1**, 536–545 (2017).

33. P. Van Bodegom, Microbial maintenance: A critical review on its quantification. *Microbial. Ecol.* **53**, 513–523 (2007).

34. J. Soini, C. Falschlehner, C. Mayer, D. Böhm, S. Weinel, J. Panula, A. Vasala, P. Neubauer, Transient increase of ATP as a response to temperature up-shift in *Escherichia coli*. *Microb. Cell Fact.* **4**, 9 (2005).

35. Y. Shechter, D. Rafaeli-Eshkol, A. Hershko, Influence of protease inhibitors and energy metabolism on intracellular protein breakdown in starving *Escherichia coli*. *Biochem. Biophys. Res. Commun.* **54**, 1518–1524 (1973).

36. C. A. Mason, J. Dünner, P. Indra, T. Colangelo, Heat-induced expression and chemically induced expression of the *Escherichia coli* stress protein HtpG are affected by the growth environment. *Appl. Environ. Microbiol.* **65**, 3433–3440 (1999).

37. M. A. Lobritz, P. Belenky, C. B. Porter, A. Gutierrez, J. H. Yang, E. G. Schwarz, D. J. Dwyer, A. S. Khalil, J. J. Collins, Antibiotic efficacy is linked to bacterial cellular respiration. *Proc. Natl. Acad. Sci. U.S.A.* **112**, 8173–8180 (2015).

38. F. Rojo, Carbon catabolite repression in *Pseudomonas*: Optimizing metabolic versatility and interactions with the environment. *FEMS Microbiol. Rev.* **34**, 658–684 (2010).

39. S. Waschina, G. D'Souza, C. Kost, C. Kaleta, Metabolic network architecture and carbon source determine metabolite production costs. *FEBS J.* **283**, 2149–2163 (2016).

40. O. Paliy, T. S. Gunasekera, Growth of *E. coli* BL21 in minimal media with different gluconeogenic carbon sources and salt contents. *Appl. Microbiol. Biotechnol.* **73**, 1169–1172 (2007).

41. W. A. Craig, S. M. Bhavnani, P. G. Ambrose, The inoculum effect: Fact or artifact?. *Diagn. Microbiol. Infect. Dis.* **50**, 229–230 (2004).

42. S. Meylan, C. B. Porter, J. H. Yang, P. Belenky, A. Gutierrez, M. A. Lobritz, J. Park, S. H. Kim, S. M. Moskowitz, J. J. Collins, Carbon sources tune antibiotic susceptibility in *Pseudomonas aeruginosa* via tricarboxylic acid cycle control. *Cell chemical biology* **24**, 195–206 (2017).

43. J. H. Yang, S. N. Wright, M. Hamblin, D. McCloskey, M. A. Alcantar, L. Schrübbers, A. J. Lopatkin, S. Satish, A. Nili, B. O. Palsson, G. C. Walker, A white-box machine learning approach for revealing antibiotic mechanisms of action. *Cell* **177**, 1649–1661.e9 (2019).

44. M. A. Kohanski, D. J. Dwyer, B. Hayete, C. A. Lawrence, J. J. Collins, A common mechanism of cellular death induced by bactericidal antibiotics. *Cell* **130**, 797–810 (2007).

45. K. R. Allison, M. P. Brynildsen, J. J. Collins, Metabolite-enabled eradication of bacterial persisters by aminoglycosides. *Nature* **473**, 216–220 (2011).

46. J. M. Stokes, A. J. Lopatkin, M. A. Lobritz, J. J. Collins, Bacterial metabolism and antibiotic efficacy. *Cell Metab.* **30**, 251–259 (2019).

47. S. I. Polianciuc, A. E. Gurzău, B. Kiss, M. G. Ştefan, F. Loghin, Antibiotics in the environment: Causes and consequences. *Med. Pharm. Rep.* **93**, 231–240 (2020).

48. S. A. Kraemer, A. Ramachandran, G. G. Perron, Antibiotic pollution in the environment: From microbial ecology to public policy. *Microorganisms* **7**, 180 (2019).

49. T. Baba, T. Ara, M. Hasegawa, Y. Takai, Y. Okumura, M. Baba, K. A. Datsenko, M. Tomita, B. L. Wanner, H. Mori, Construction of *Escherichia coli* K-12 in-frame, single-gene knockout mutants: The Keio collection. *Mol. Syst. Biol.* **2**, 2006–0008 (2006.0008).

50. H. Prensky, A. Gomez-Simmonds, A. C. Uhlemann, A. J. Lopatkin, Conjugation dynamics depend on both the plasmid acquisition cost and the fitness cost. *Mol. Syst. Biol.* **17**, e9913 (2021).

51. L. Heirendt, S. Arreckx, T. Pfau, S. N. Mendoza, A. Richelle, A. Heinken, H. S. Haraldsdóttir, J. Wachowiak, S. M. Keating, V. Vlasov, S. Magnusdóttir, Creation and

analysis of biochemical constraint-based models using the COBRA Toolbox v. 3.0. *Nat. Protoc.* **14**, 639–702 (2019).

52. J. D. Orth, T. M. Conrad, J. Na, J. A. Lerman, H. Nam, A. M. Feist, B. Ø. Palsson, A comprehensive genome-scale reconstruction of *Escherichia coli* metabolism—2011. *Mol. Syst. Biol.* **7**, 535 (2011).

53. W. E. Deming, *Statistical Adjustment of Data* (John Wiley & Sons, 1943; reprinted by Dover Publications Inc., 1943).

54. T. Strutz, *Data Fitting and Uncertainty: A Practical Introduction to Weighted Least Squares and Beyond* (Springer, 2011).

55. F. Soriano, C. Ponte, M. Santamaria, M. Jimenez-Arriero, Relevance of the inoculum effect of antibiotics in the outcome of experimental infections caused by *Escherichia coli*. *J. Antimicrob. Chemother.* **25**, 621–627 (1990).

56. F. Soriano, R. Edwards, D. Greenwood, Effect of inoculum size on bacteriolytic activity of cefminox and four other beta-lactam antibiotics against *Escherichia coli*. *Antimicrob. Agents Chemother.* **36**, 223–226 (1992).

57. B. J. McGrath, S. L. Kang, G. W. Kaatz, M. J. Rybak, Bactericidal activities of teicoplanin, vancomycin, and gentamicin alone and in combination against *Staphylococcus aureus* in an in vitro pharmacodynamic model of endocarditis. *Antimicrob. Agents Chemother.* **38**, 2034–2040 (1994).

58. N. Cotroneo, R. Harris, N. Perlmutter, T. Beveridge, J. A. Silverman, Daptomycin exerts bactericidal activity without lysis of *Staphylococcus aureus*. *Antimicrob. Agents Chemother.* **52**, 2223–2225 (2008).

59. E. Bidlas, T. Du, R. J. Lambert, An explanation for the effect of inoculum size on MIC and the growth/no growth interface. *Int. J. Food Microbiol.* **126**, 140–152 (2008).

60. D. Molenaar, R. Van Berlo, D. De Ridder, B. Teusink, Shifts in growth strategies reflect tradeoffs in cellular economics. *Mol. Syst. Biol.* **5**, 323 (2009).

61. S. Manzoni, P. Taylor, A. Richter, A. Porporato, G. I. Ågren, Environmental and stoichiometric controls on microbial carbon-use efficiency in soils. *New Phytol.* **196**, 79–91 (2012).

62. H. Akashi, T. Gojobori, Metabolic efficiency and amino acid composition in the proteomes of *Escherichia coli* and *Bacillus subtilis*. *Proc. Natl. Acad. Sci. U.S.A.* **99**, 3695–3700 (2002).

63. A. Gschaedler, J. Boudrant, Amino acid utilization during batch and continuous cultures of *Escherichia coli* on a semi-synthetic medium. *J. Biotechnol.* **37**, 235–251 (1994).

64. P. J. Lahtvee, K. Adamberg, L. Arike, R. Nahku, K. Aller R. Vilu, Multi-omics approach to study the growth efficiency and amino acid metabolism in *Lactococcus lactis* at various specific growth rates. *Microb. Cell Fact.* **10**, 1–12 (2011).

65. K. Piir, A. Paier, A. Liiv, T. Tenson, Ü. Maiväli, Ribosome degradation in growing bacteria. *EMBO Rep.* **12**, 458–462 (2011).

66. C. Petibon, M. Malik Ghulam, M. Catala, S. Abou Elela, Regulation of ribosomal protein genes: An ordered anarchy*: Wiley Interdiscip. Rev. RNA* **12**, e1632 (2021).

67. M. Nomura, J. L. Yates, D. Dean, L. E. Post, Feedback regulation of ribosomal protein gene expression in *Escherichia coli*: Structural homology of ribosomal RNA and ribosomal protein MRNA. *Proc. Natl. Acad. Sci. U.S.A.* **77**, 7084–7088 (1980).

68. A. Crabbe, L. Ostyn, S. Staelens, C. Rigauts, M. Risseeuw, M. Dhaenens, S. Daled, H. Van Acker, D. Deforce, S. Van Calenbergh, T. Coenye, Host metabolites stimulate the bacterial proton motive force to enhance the activity of aminoglycoside antibiotics. *PLOS Pathog.* **15**, e1007697 (2019).

69. A. J. Lee, S. Wang, H. R. Meredith, B. Zhuang, Z. Dai, L. You, Robust, linear correlations between growth rates and β-lactam–mediated lysis rates. *Proc. Natl. Acad. Sci. U.S.A.* **115**, 4069–4074 (2018).

70. R. J. Wallace, W. H. Holms, Maintenance coefficients and rates of turnover of cell material in *Escherichia coli* ML308 at different growth temperatures. *FEMS Microbiol. Lett.* **37**, 317–320 (1986).

71. A. D. Hollenbach, K. A. Dickson, M. W. Washabaugh, Thiamin transport in *Escherichia coli*: The mechanism of inhibition by the sulfhydryl-specific modifier N-ethylmaleimide. *Biochim. Biophys. Acta Biomembr.* **1564**, 421–428 (2002).

72. J. Glazyrina, E. M. Materne, T. Dreher, D. Storm, S. Junne, T. Adams, G. Greller, P. Neubauer, High cell density cultivation and recombinant protein production with *Escherichia coli* in a rocking-motion-type bioreactor. *Microb. Cell Fact.* **9**, 1–11 (2010).

73. M. Zampieri, M. Hörl, F. Hotz, N. F. Müller, U. Sauer, Regulatory mechanisms underlying coordination of amino acid and glucose catabolism in *Escherichia coli*. *Nat. Commun.* **10**, 1–13 (2019).

74. K. B. Andersen, K. von Meyenburg, Are growth rates of *Escherichia coli* in batch cultures limited by respiration? *J. Bacteriol.* **144**, 114–123 (1980).

75. Y. Liu, K. Yang, Y. Jia, J. Shi, Z. Tong, Z. Wang,Cysteine potentiates bactericidal antibiotics activity against gram-negative bacterial persisters. *Infect. Drug Resist.* **13**, 2593–2599 (2020).

76. R. P. Smith, A. Doiron, R. Muzquiz, M. C. Fortoul, M. Haas, T. Abraham, R. J. Quinn, I. Barraza, K. Chowdhury, L. R. Nemzer, The public and private benefit of an impure public good determines the sensitivity of bacteria to population collapse in a snowdrift game. *Environ. Microbiol.* **21**, 4330–4342 (2019).

77. J. Coates, B. R. Park, D. Le, E. Şimşek, W. Chaudhry, M. Kim, Antibiotic-induced population fluctuations and stochastic clearance of bacteria. *eLife* **7**, e32976 (2018).

78. L. Gerosa, B. R. H. van Rijsewijk, D. Christodoulou, K. Kochanowski, T. S. Schmidt, E. Noor, U. Sauer, Pseudo-transition analysis identifies the key regulators of dynamic metabolic adaptations from steady-state data. *Cell Systems* **1**, 270–282 (2015).

79. S. Lee, K. T. Kwon, H. I. Kim, H. H. Chang, J. M. Lee, P. G. Choe, W. B. Park, N. J. Kim, M. D. Oh, D. Y. Song, S. W. Kim, Clinical implications of cefazolin inoculum effect and β-lactamase type on methicillin-susceptible *Staphylococcus aureus* bacteremia. *Microbial. Drug Resist.* **20**, 568–574 (2014).

80. M. E. Ruiz, I. C. Guerrero, C. U. Tuazon, Endocarditis caused by methicillin-resistant *Staphylococcus aureus*: Treatment failure with linezolid. *Clin. Infect. Dis.* **35**, 1018–1020 (2002).

81. J. P. Hornak, S. Anjum, D. Reynoso, Adjunctive ceftaroline in combination with daptomycin or vancomycin for complicated methicillin-resistant *Staphylococcus aureus* bacteremia after monotherapy failure. *Ther. Adv. in Infect. Dis.* **6**, 1–10 (2019).

82. E. J. Goldstein, D. M. Citron, M. L. Corrado, Effect of inoculum size on in vitro activity of norfloxacin against fecal anaerobic bacteria: Rationale for selective decontamination of the digestive tract. *Am. J. Med.* **82**, 84–87 (1987).

83. M. T. Reymann, H. P. Holley Jr., C. G. Cobbs, Persistent bacteremia in staphylococcal endocarditis. *Am. J. Med.* **65**, 729–737 (1978).

84. K. Lam, A. S. Bayer, Serious infections due to group G streptoccocci: Report of 15 cases with in vitro-in vivo correlations. *Am. J. Medicine* **75**, 561–570 (1983).

85. T. Kim, S. C. Lee, M. Bae, H. Sung, M. N. Kim, J. Jung, M. J. Kim, S. H. Kim, S. O. Lee, S. H. Choi, and Y. S. Kim, In vitro activities and inoculum effects of ceftazidime-avibactam and aztreonam-avibactam against carbapenem-resistant enterobacterales isolates from South Korea. *Antibiotics* **9**, 912 (2020).

86. E. Bingen, N. Lambert-Zechovsky, P. Mariani-Kurkdjian, C. Doit, Y. Aujard, F. Fournerie, H. Mathieu, Bacterial counts in cerebrospinal fluid of children with meningitis. *Eur. J. Clin. Microbiol. Infect. Dis.* **9**, 278–281 (1990).

87. C. König, H. Simmen, J. Blaser, Bacterial concentrations in pus and infected peritoneal fluid—Implications for bactericidal activity of antibiotics. *J. Antimicrob. Chemother.* **42**, 227–232 (1998).

88. I. Brook, Aerobic and anaerobic bacteriology of cholesteatoma. *Laryngoscope* **91**, 250–253 (1981).

89. D. Bilton, A. Pye, M. M. Johnson, J. L. Mitchell, M. Dodd, A. K. Webb, R. A. Stockley, S. L. Hill, The isolation and characterization of non-typeable *Haemophilus influenzae* from the sputum of adult cystic fibrosis patients. *Eur. Respir. J.* **8**, 948–953 (1995).

90. S. J. Morgan, S. I. Lippman, G. E. Bautista, J. J. Harrison, C. L. Harding, L. A. Gallagher, A. C. Cheng, R. Siehnel, S. Ravishankar, M. L. Usui, J. E. Olerud, Bacterial fitness in chronic wounds appears to be mediated by the capacity for high-density growth, not virulence or biofilm functions. *PLOS Pathog.* **15**, e1007511 (2019).

91. R. Jain, R. Srivastava, Metabolic investigation of host/pathogen interaction using MS2-infected *Escherichia coli*. *BMC Syst. Biol.* **3**, 1–11 (2009).

92. S. Pinhal, D. Ropers, J. Geiselmann, H. D. Jong, W. W. Metcalf, Acetate metabolism and the inhibition of bacterial growth by acetate. *J. Bacteriol.* **201**, e00147-19 (2019).

93. Q. Hua, A. R. Joyce, B. Ø. Palsson, S. S. Fong, Metabolic characterization of *Escherichia coli* strains adapted to growth on lactate. *Appl. Environ. Microbiol.* **73**, 4639–4647 (2007).

94. R. Yao, D. Xiong, H. Hu, M. Wakayama, W. Yu, X. Zhang, K. Shimizu, Elucidation of the co-metabolism of glycerol and glucose in *Escherichia coli* by genetic engineering, transcription profiling, and 13 C metabolic flux analysis. *Biotechnol. Biofuels* **9**, 1–14 (2016).

95. B. M. Hogema, J. C. Arents, R. Bader, P. W. Postma, Autoregulation of lactose uptake through the LacYpermease by enzyme IIAGlc of the PTS in *Escherichia coli* K-12. *Mol. Microbiol.* **31**, 1825–1833 (1999).

96. H. J. Seong, J. E. Woo, Y.-S. Jang, Control of the galactose-to-glucose consumption ratio in co-fermentation using engineered *Escherichia coli* strains. *Sci. Rep.* **10**, 1–8 (2020).

97. K. Olavarria, A. Fina, M. I. Velasco, M. C. van Loosdrecht, S. A. Wahl, Metabolism of sucrose in a non-fermentative *Escherichia coli* under oxygen limitation. *Appl. Microbiol. Biotechnol.* **103**, 6245–6256 (2019).

98. H. G. Lawford, J. D. Rousseau, Relative rates of sugar utilization by an ethanologenic recombinant *Escherichia coli* using mixtures of glucose, mannose, and xylose. *Appl. Biochem. Biotechnol.* **45-46**, 367–381 (1994).

99. J. Lengeler, E. Lin, Reversal of the mannitol-sorbitol diauxie in *Escherichia coli*. *J. Bacteriol.* **112**, 840–848 (1972).

100. U. Lendenmann, M. Snozzi, T. Egli, Kinetics of the simultaneous utilization of sugar mixtures by *Escherichia coli* in continuous culture. *Appl. Environ. Microbiol.* **62**, 1493–1499 (1996).

101. P. S. Andersen, D. Frees, R. Fast, B. Mygind, Uracil uptake in *Escherichia coli* K-12: Isolation of uraA mutants and cloning of the gene. *J. Bacteriol.* **177**, 2008–2013 (1995).
